# iLAP: a workflow-driven software for experimental protocol development, data acquisition and analysis

**DOI:** 10.1186/1471-2105-10-390

**Published:** 2009-11-26

**Authors:** Gernot Stocker, Maria Fischer, Dietmar Rieder, Gabriela Bindea, Simon Kainz, Michael Oberstolz, James G McNally, Zlatko Trajanoski

**Affiliations:** 1 Institute for Genomics and Bioinformatics, Graz University of Technology, Petersgasse 14, 8010 Graz, Austria; 2 Center for Cancer Research Core Imaging Facility, Laboratory of Receptor Biology and Gene Expression, National Cancer Institute, National Institutes of Health, 41 Library Drive, Bethesda, MD 20892, USA

## Abstract

**Background:**

In recent years, the genome biology community has expended considerable effort to confront the challenges of managing heterogeneous data in a structured and organized way and developed laboratory information management systems (LIMS) for both raw and processed data. On the other hand, electronic notebooks were developed to record and manage scientific data, and facilitate data-sharing. Software which enables both, management of large datasets and digital recording of laboratory procedures would serve a real need in laboratories using medium and high-throughput techniques.

**Results:**

We have developed iLAP (Laboratory data management, Analysis, and Protocol development), a workflow-driven information management system specifically designed to create and manage experimental protocols, and to analyze and share laboratory data. The system combines experimental protocol development, wizard-based data acquisition, and high-throughput data analysis into a single, integrated system. We demonstrate the power and the flexibility of the platform using a microscopy case study based on a combinatorial multiple fluorescence in situ hybridization (m-FISH) protocol and 3D-image reconstruction. iLAP is freely available under the open source license AGPL from http://genome.tugraz.at/iLAP/.

**Conclusion:**

iLAP is a flexible and versatile information management system, which has the potential to close the gap between electronic notebooks and LIMS and can therefore be of great value for a broad scientific community.

## Background

The development of novel large-scale technologies has considerably changed the way biologists perform experiments. Genome biology experiments do not only generate a wealth of data, but they often rely on sophisticated laboratory protocols comprising hundreds of individual steps. For example, the protocol for chromatin immunoprecipitation on a microarray (Chip-chip) has 90 steps, uses over 30 reagents and 10 different devices [1]. Even adopting an established protocol for large-scale studies represents a daunting challenge for the majority of the labs. The development of novel laboratory protocols and/or the optimization of existing ones is still more distressing, since this requires systematic changes of many parameters, conditions, and reagents. Such changes are becoming increasingly difficult to trace using paper lab books. A further complication for most protocols is that many laboratory instruments are used, which generate electronic data stored in an unstructured way at disparate locations. Therefore, protocol data files are seldom or never linked to notes in lab books and can be barely shared within or across labs. Finally, once the experimental large-scale data have been generated, they must be analyzed using various software tools, then stored and made available for other users. Thus, it is apparent that software support for current biological research - be it genomic or performed in a more traditional way - is urgently needed and inevitable.

In recent years, the genome biology community has expended considerable effort to confront the challenges of managing heterogeneous data in a structured and organized way and as a result developed information management systems for both raw and processed data. Laboratory information management systems (LIMS) have been implemented for handling data entry from robotic systems and tracking samples [2,3] as well as data management systems for processed data including microarrays [4,5], proteomics data [6-8], and microscopy data [9]. The latter systems support community standards like FUGE[10,11], MIAME [12], MIAPE [13], or MISFISHIE [14] and have proven invaluable in a state-of-the-art laboratory. In general, these sophisticated systems are able to manage and analyze data generated for only a single type or a limited number of instruments, and were designed for only a specific type of molecule.

On the other hand, commercial as well as open source electronic notebooks [15-19] were developed to record and manage scientific data, and facilitate data-sharing. The influences encouraging the use of electronic notebooks are twofold [16]. First, much of the data that needs to be recorded in a laboratory notebook is generated electronically. Transcribing data manually into a paper notebook is error-prone, and in many cases, for example, analytical data (spectra, chromatograms, photographs, etc.), transcription of the data is not possible. Second, the incorporation of high-throughput technologies into the research process has resulted in an increased volume of electronic data that need to be transcribed. As opposed to LIMS, which captures highly structured data through rigid user interfaces with standard report formats, electronic notebooks contain unstructured data and have flexible user interfaces.

Software which enables both, management of large datasets and recording of laboratory procedures, would serve a real need in laboratories using medium and high-throughput techniques. To the best of our knowledge, there is no software system available, which supports tedious protocol development in an intuitive way, links the plethora of generated files to the appropriate laboratory steps and integrates further analysis tools. We have therefore developed iLAP, a workflow-driven information management system for protocol development and data management. The system combines experimental protocol development, wizard-based data acquisition, and high-throughput data analysis into a single, integrated system. We demonstrate the power and the flexibility of the platform using a microscopy case study based on combinatorial multiple fluorescence in situ hybridization (m-FISH) protocol and 3D-image reconstruction.

## Implementation

### Workflow-driven software design

**Figure 1:**
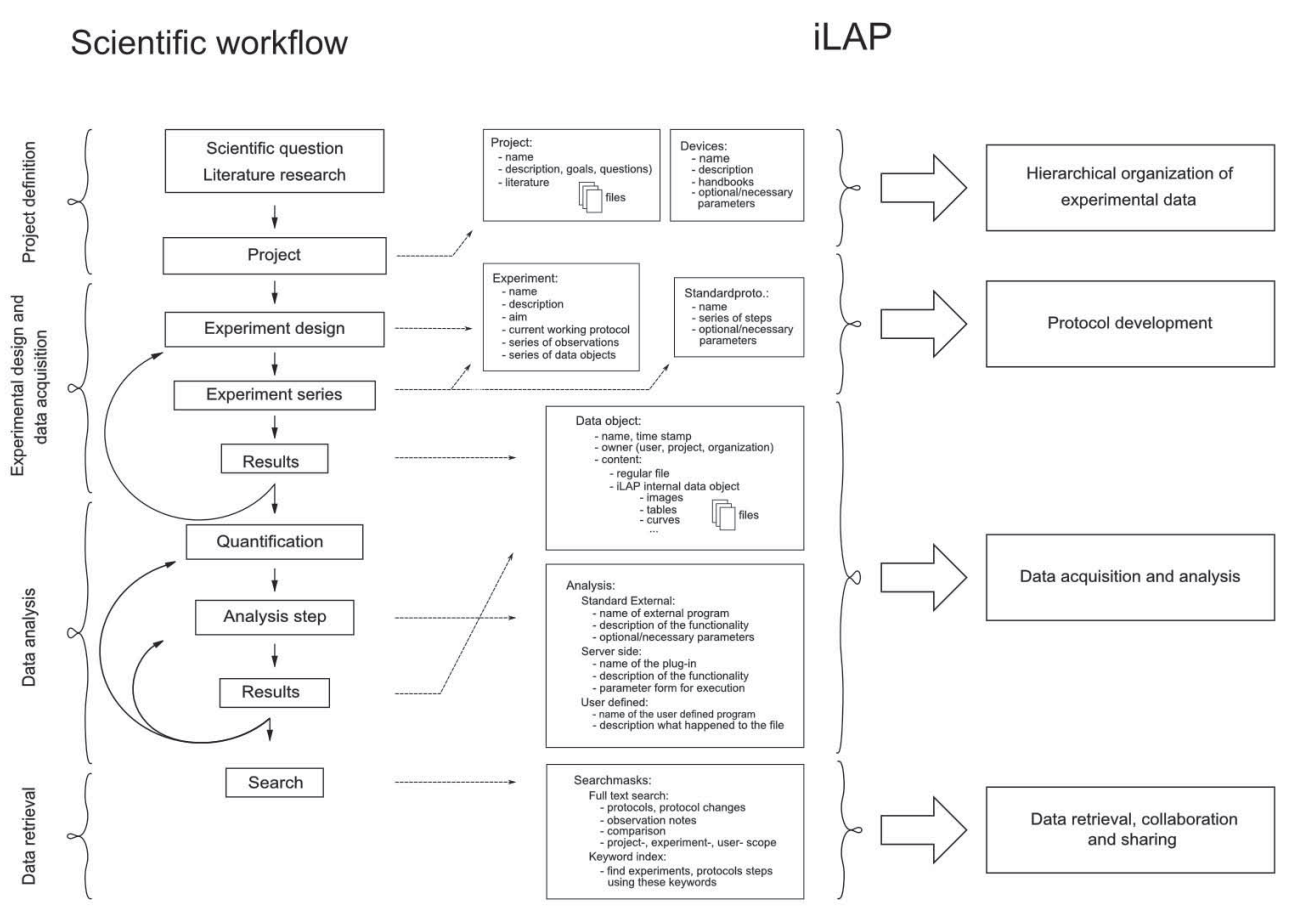
**Mapping of the laboratory workflow onto iLAP features**. The software design of iLAP is inspired by a typical laboratory workflow in life sciences and offers software assistance during the process. The figure illustrates on the left panel the scientific workflow separated into four phases: project definition, data acquisition and analysis, and data retrieval. The right panel shows the main functionalities offered by iLAP.

The design of a software platform that supports the development of protocols and data management in an experimental context has to be based on and directed by the laboratory workflow. The laboratory workflow can be divided into four principal steps: 1) project definition phase, 2) experimental design and data acquisition phase, 3) data analysis and processing phase and 4) data retrieval phase (Figure 1).

#### Project definition phase

A scientific project starts with a hypothesis and the choice of methods required to address a specific biological question. Already during this initial phase it is crucial to define the question as specifically as possible and to capture the information in a digital form. Documents collected during the literature research should be collated with the evolving project definition for later review or for sharing with other researchers. All files collected in this period should be attached to the defined projects and experiments in the software.

#### Experimental design and data acquisition

Following the establishment of a hypothesis and based on preliminary experiments, the detailed design of the biological experiments is then initiated. Usually, the experimental work follows already established standard operating procedures, which have to be modified and optimized for the specific biological experiment. These protocols are defined as a sequence of protocol steps. However, well-established protocols must be kept flexible in a way that particular conditions can be changed. The typically changing parameters of standard protocol steps (e.g. fixation times, temperature changes etc.) are important to record as they are used to improve the experimental reproducibility.

Equipped with a collection of standard operating procedures, an experiment can be initiated and the data generated. In general, data acquisition comprises not only files but also observations of interest, which might be relevant for the interpretation of the results. Most often these observations disappear in paper notebooks and are not accessible in a digital form. Hence, these experimental notes should be stored and attached to the originating protocol step, experiment or project.

#### Data analysis and processing

After storing the raw result files, additional analysis and post-processing steps must be performed to obtain processed data for subsequent analysis. In order to extract information and to combine it in a statistically meaningful manner, multiple data sets have to be acquired. The software workflow should enable also the inclusion of external analytical steps, so that files resulting from external analysis software can be assigned to their original raw data files. Finally, the data files generated at the analysis stage should be connected to the raw data, allowing connection of the data files with the originating experimental context.

#### Data retrieval

By following the experimental workflow, all experimental data e.g. different files, protocols, notes etc. should be organized in a chronological and project-oriented way and continuously registered during their acquisition. An additional advantage should be the ability to search and retrieve the data. Researchers frequently have to search through notebooks to find previously uninterpretable observations. Subsequently, as the project develops, the researchers gain a different perspective and recognize that prior observations could lead to new discoveries. Therefore, the software should offer easy to use interfaces that allow searches through observation notes, projects- and experiment descriptions.

### Software Architecture

**Figure 2:**
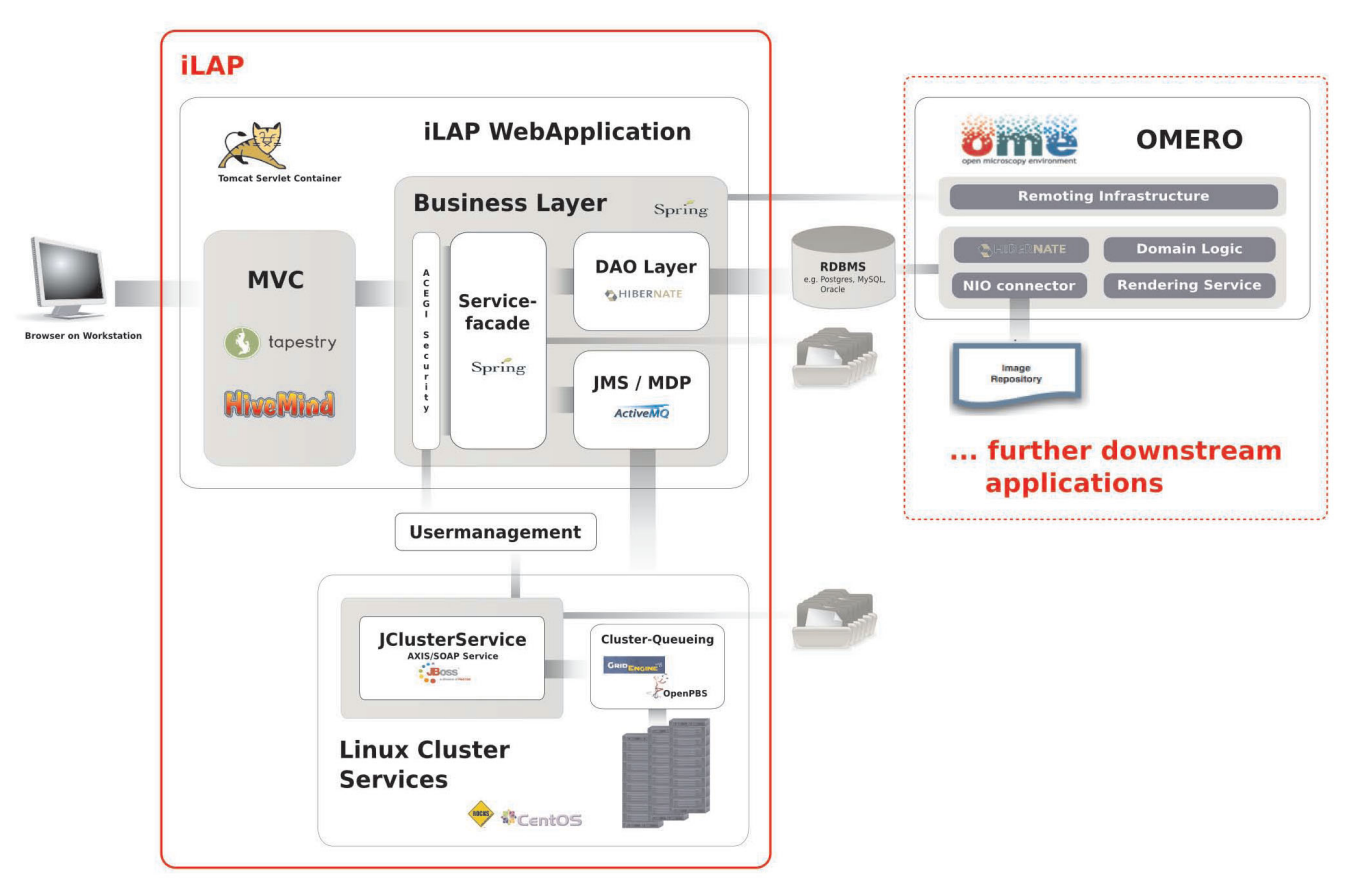
**Software Architecture**. iLAP features a typical three-tier architecture and can hence be divided into a presentation tier, business tier and a persistence tier (from left to right). The presentation tier is formed by a graphical user interface, accessed using a web browser. The following business layer is protected by a security layer, which enforces user authentication and authorization. After access is granted, the security layer passes the user requests to the business layer, which is mainly responsible for guiding the user through the laboratory workflow. This layer also coordinates all background tasks like automatic surveying of analysis jobs on a computing cluster or synchronizing/exchanging data with further downstream applications. (e.g. OMERO (open microscopy environment) image server). Finally, the persistence layer interacts with the relational database.

iLAP is a multi-tier client-server application and can be subdivided into different functional modules which interact as self-contained units according to their defined responsibilities (see Figure 2).

#### Presentation tier

The presentation tier within iLAP is formed by a Web interface, using Tapestry [20] as the model view controller and an Axis Web service [21], which allows programming access to parts of the application logic. Thus, on the client side, a user requires an Internet connection and a recent Web browser with Java Applet support, available for almost every platform. In order to provide a simple, consistent but also attractive Web interface, iLAP follows usability guidelines described in [22,23] and uses Web 2.0 technologies for dynamic content generation.

#### Business tier and runtime environment

The business tier is realized as view-independent application logic, which stores and retrieves datasets by communicating with the persistence layer. The internal management of files is also handled from a central service component, which persists the meta-information for acquired files to the database, and stores the file content in a file-system-based data hierarchy. The business layer also holds asynchronous services for application-internal JMS messaging and for integration of external computing resources like high-performance computing clusters. All services of this layer are implemented as Spring [24] beans, for which the Spring-internal interceptor classes provide transactional integrity.

The business tier and the persistence tier are bound by the Spring J2EE lightweight container, which manages the component-object life cycle. Furthermore, the Spring context is transparently integrated into the Servlet context of Tapestry using the HiveMind [25] container backend. This is realized by using the automatic dependency injection functionality of HiveMind which avoids integrative glue code for lookups into the Spring container. Since iLAP uses Spring instead of EJB related components, the deployment of the application only requires a standard conformed Servlet container. Therefore, the Servlet container Tomcat [26] is used, which offers not only Servlet functionality but J2EE infrastructure services [27] such as centrally configured data-sources and transaction management realized with the open source library JOTM [28]. This makes the deployment of iLAP on different servers easier, because machine-specific settings for different production environments are kept outside the application configuration.

#### External programming interfaces

The SOAP Web service interface for external programmatic access is realized by combining the Web service framework Axis with corresponding iLAP components. The Web service operates as an external access point for Java Applets within the Web application, as well as for external analysis and processing applications such as ImageJ.

#### Model driven development

In order to reduce coding and to increase the long term maintainability, the model driven development environment AndroMDA [29] is used to generate components of the persistence layer and recurrent parts from the above mentioned business layer. AndroMDA accomplishes this by translating an annotated UML-model into a JEE-platform-specific implementation using Hibernate and Spring as base technology. Due to the flexibility of AndroMDA, application external services, such as the user management system, have a clean integration in the model. Dependencies of internal service components on such externally defined services are cleanly managed by its build system.

By changing the build parameters in the AndroMDA configuration, it is also possible to support different relational database management systems. This is because platform specific code with the same functionality is generated for data retrieval. Furthermore, technology lock-in regarding the implementation of the service layers was also addressed by using AndroMDA, as the implementation of the service facade can be switched during the build process from Spring based components to distributed Enterprise Java Beans. At present, iLAP is operating on one local machine and, providing the usage scenarios do not demand it, this architectural configuration will remain. However, chosen technologies are known to work on Web server farms and crucial distribution of the application among server nodes is transparently performed by the chosen technologies.

#### Asynchronous data processing

The asynchronous handling of business processes is realized in iLAP with message-driven Plain Old Java Objects (POJOs). Hence, application tasks, such as the generation of image previews, can be performed asynchronously. If performed immediately, these would unnecessarily block the responsiveness of the Web front-end. iLAP delegates tasks via JMS messages to back-end services, which perform the necessary processing actions in the background.

These back-end services are also UML-modelled components and receive messages handled by the JMS provider ActiveMQ. If back-end tasks consume too many calculation resources, the separation of Web front-end and JMS message receiving services can be realized by copying the applications onto two different servers and changing the Spring JMS configuration.

For the smooth integration of external computing resources like the high-performance computing cluster or special compute nodes with limited software licenses the JClusterService is used. JClusterService is a separately developed J2EE application which enables a programmer to run generic applications on a remote execution host or high-performance computing cluster. Every application which offers a command line interface can be easily integrated by defining a service definition in XML format and accessing it via a SOAP-based programming interface from any Java-application. The execution of the integrated application is carried out either by using the internal JMS-queuing system for single host installations or by using the open source queuing systems like Sun Grid Engine (Sun Microsystems) or OpenPBS/Torque.

## Results

### Functional overview

The functionality offered by the iLAP web interface can be described by four components: 1) hierarchical organization of the experimental data, 2) protocol development, 3) data acquisition and analysis, and 4) data retrieval and data sharing (Figure 1). iLAP specific terms are summarized in Table 1.

**Table 1:** iLAP Terminology:

iLAP specific terms	Description
Project	Logical unit which can be structured hierarchically and holds experiments, notes and other files (e.g. derived from literature research).
Experiment	Logical unit which corresponds to one biological experiment and holds a current working protocol, experiment specific documentation files, parameter values, raw files, notes, and analysis steps.
Standard protocol	Frequently used and well established protocol template also known as standard operating procedures (SOP).
Current working protocol	Sequence of protocol steps for a specific experiment which holds raw files, notes and experiment specific parameter values.
Protocol step	One single step in a protocol which is defined by a name, description, and a list of definable parameters. A sequence of protocol steps defines a protocol.
Step group	Protocol step which groups multiple protocol steps to a logical unit. It can be used as a step container for sequentially executed protocol steps or within split steps.
Split step	Protocol step which can contain multiple (step groups) which have to be executed concurrently.
Protocol step parameter	Changing parameters which are associated with a step and can hold either textual or numerical values as well as a selection from a predefined value list (enumeration).
Note	Notes are textual descriptions which are intended to be used for documenting abnormal observations at almost anywhere within iLAP.
Raw file	Raw files are files which are produced by laboratory instruments and are not processed by any analysis step captured within iLAP.
Analysis step	Description of a processing step which manipulates, analyzes or processes a raw file, and generates processed files which are linked to the original raw file. Analysis steps can be either external e.g. using external software or internal using iLAP-internal analysis modules.
Analysis step parameter	Parameters and values used during the analysis step.

#### Hierarchical organization of experimental data

**Figure 3:**
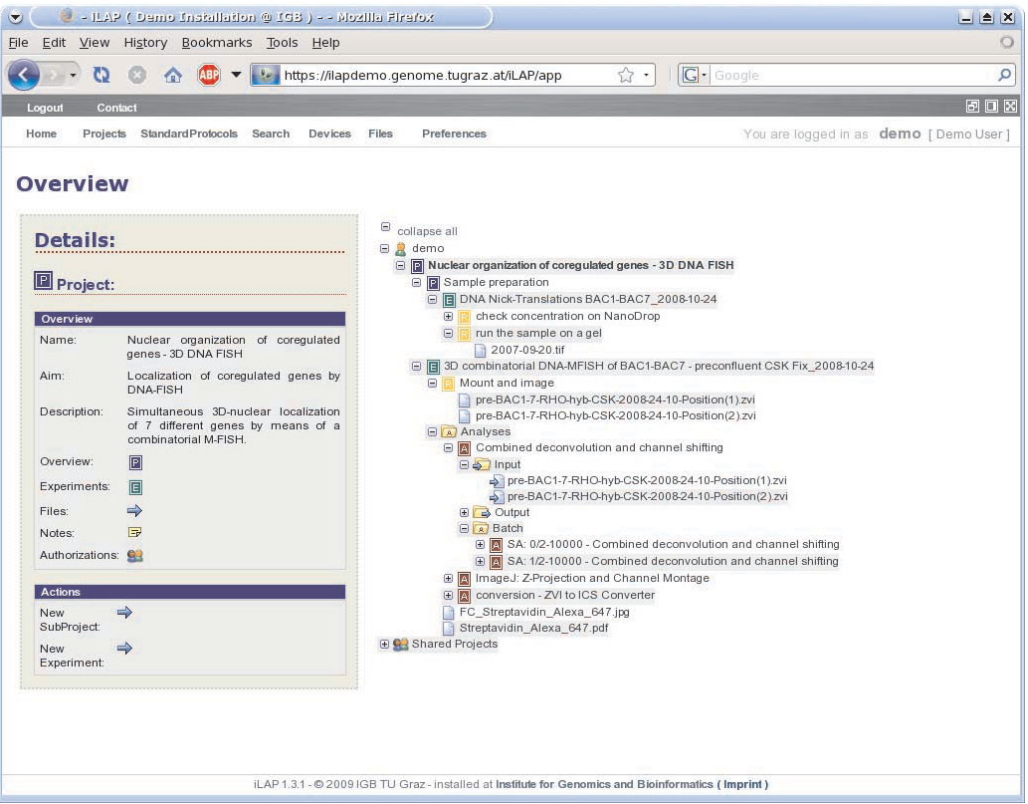
**Hierarchical organization of data in iLAP overview**. The continuous use of iLAP inherently leads to structured recording of experiments, conserving the complete experimental context of data records throughout the history of the research project. In doing so, a hierarchical structure with projects, sub-projects and experiments is created and can be displayed in this iLAP overview tree. The P-icons in the tree stand for projects and sub-projects, the E-icon for experiments and the A-icon for analysis steps. Files attached to protocol steps are considered as raw files and are therefore collected under the step container visualized with the R-icon. The consistent association of color schemes to logical units like projects, experiments, etc. can be directly recognized in this overview. By clicking on one of the tree icons on the left hand a detailed overview appears about the selected item. Also actions like creation of new projects etc. can be directly initiated using the quick-links in the "Actions" section of "Details".

This part of the user interface covers the project definition phase of the experimental workflow. The definition of projects and experiments consists solely in inserting the required descriptive parameters via a Web form. In doing so, a hierarchical structure with projects, sub-projects and experiments is created and displayed in the iLAP overview. The hierarchy (Figure 3) and other screen shots can be found in the iLAP user manual (Additional file 1). This overview is the starting point of iLAP, from which almost every activity can be initiated. By navigating through the tree, an information box appears alongside. This box details information about the current node in the tree and the operations which can be performed on the database managed object represented by the node. Already in this early stage, files derived from literature research can be uploaded to projects and experiments, and ongoing observations can be stored using the general note dialog. If multiple files must be associated with projects and experiments, a Java Applet can be used to upload the files to the generated project/experiment structure. iLAP can manage every file independent of their file type, and can thus be considered as a generic document management system. File types only need to be considered for subsequent processing and data extraction.

#### Protocol development

When starting experimental work, the iLAP facility manager should define commonly used standard protocols using the protocol development masks. Therefore, a sequence of steps must be defined which describes the typical ongoing experiment in detail. Dynamic protocol parameters, which may be adapted for protocol optimization during the experiment, can be associated with the pre-defined steps. These parameters can be either numerical values, descriptive text or predefined enumeration types, all of which can be preset by default values and marked with appropriate units. In order to force the acquisition of critical parameters in the data acquisition wizard, parameters can be marked as required. According to our experience and the experience of other users, it is helpful to define small and reusable standard protocol units, which can be used as building blocks during the experiment-specific protocol assembly. Automatic internal versioning takes care of changes in standard protocols so that dependent protocols used in previous experiments remain unaffected.

Equipped with a collection of standard protocols, an experiment can be initiated and should be defined at the beginning of the workflow. The name of each experiment, its general description and specific aims, must be provided in order to be able to distinguish between different experiments. The detailed experiment procedure is defined by its current working protocol which can be composed step by step or by reusing existing current working protocols from already performed experiments. If the experiment is following a standard protocol, the current working protocol should be created by simply copying the predefined standard protocol steps and parameter definitions. In order to consider also the concurrent nature of simultaneously executed steps the experimenter should be able to define different sub-branches (e.g. cells are treated with different drugs in order to study their response) named split steps. These split steps lead to different branches of the experimental workflow called step groups which are separately handled during the data acquisition phase.

Once the protocol design phase is completed and all necessary protocol steps with their parameters are defined the researchers should be able to generate a printout of the current working protocol with which the experiment can be performed at the lab bench.

#### Data acquisition and analysis

After having finished all experimental work and having created raw data files with different laboratory instruments the data acquisition within iLAP should be performed. By going through the early defined current working protocol steps, generated raw data files, used protocol parameter values and observation notes must be entered. Wizard-based input masks (wizard), which are derived from the defined current protocol steps, assist the experimenters during this work. On every step the user has to fill in the value fields for required parameters and can attach files and notes to each of the steps. During the creation of the working protocol, it is important to name those steps to which files are attached in a descriptive way. Files that are directly connected to experimental steps are considered as raw files and are protected against deletion. Note, files can be linked to the protocol steps anywhere in iLAP, i.e. also before and after the data acquisition.

For this data association, the iLAP workflow offers also the possibility to transfer all generated files to a central repository and associate automatically files with their generating protocol step at once, using a Java Applet. All the internal linkages to protocol steps, experiments or projects are performed automatically without the need of any user interference. As the files are attached to a protocol and an experiment, the overall context is preserved and the likelihood of reproducibility of the same conditions is increased. Within iLAP experimental notes are stored and attached to the originating protocol step, experiment or project and are retrievable using a keyword based search mask

#### Data analysis

The analysis steps are recorded in iLAP by either reusing existing analysis templates or describing new analysis steps applied to the previously uploaded raw data files. Additional analysis tools can be developed in Java as described in the iLAP user manual (Additional file 1). According to the file type, internally implemented analysis steps or the description of externally performed analysis steps are associated with the raw data files. Result files from analysis programs together with the used parameters can be easily attached to analysis definitions. As an example, a server analysis tool was implemented for deconvolving three dimensional image stacks, executed on a remote high-performance computing cluster using the JClusterService (see methods).

#### Integration of external programs

A proof of concept about external access of programs using the iLAP application programming interface was shown by the implementation of a plugin for the widely used image processing software ImageJ [30,31]. This Java plugin enables ImageJ to transfer the image files directly from iLAP to the client machine. This functionality appears as a regular dialog in the graphical user interface of ImageJ, and allows upload of result files back into iLAP in a transparent manner.

#### Automatic post processing tool chain

Background tasks like the generation of previews are performed using the internal post-processing tool chain which is started asynchronously as soon as the files are associated with the originating experiment in iLAP. According to the detected file type, multiple post-processor steps are executed and results are automatically stored back into the database. This flexible system approach is also used to automatically inform and synchronize further downstream applications like OMERO [9] image server from the Open Microscopy Environment project. Therefore, iLAP is able to transfer files - transparently for the user - to a server where a comparable project/dataset structure is created.

#### Data retrieval and information sharing

The use of the described data acquisition features inherently leads to structured recording of experiments, conserving the complete experimental context of data records throughout the history of research projects. It is often necessary to go back to already completed experiments and to search through old notes. Therefore, iLAP offers search masks which allow keyword based searching in the recorded projects, experiments and notes. These results are often discussed with collaboration partners to gain different opinions on the same raw data.

In order to allow direct collaboration between scientists iLAP is embedded into a central user management system [4] which offers multiple levels of access control to projects and their associated experimental data. The sharing of projects can be done on a per-user basis or on an institutional basis. For small or local single-user installations, the fully featured user management system can be replaced by a file-based user management which still offers the same functionalities from the sharing point of view, but lacks institute-wide functionalities (Additional file 2). This is only possible because iLAP keeps the source of user accounts separated from the internal access control to enable easy integration of additional local or institution wide user management systems.

Since sophisticated protocols are crucial for successful experiments iLAP-users can export their protocols not only in PDF format (Additional file 3) but also in an exchangeable XML format (Additional file 4 and 5). In that way scientists can directly pass over their optimized protocols to partners who do not share the data using iLAP internally but need to get the protocol information transferred. The same XML files can be also used on a broader basis for protocol exchange using central eScience platforms like MyExperiments [32]. This platform aims for an increased reuse and repurpose of commonly shared workflows achieving at the same time reduced time-to-experiment and avoiding reinvention. Ongoing standardization efforts regarding the XML format like FuGE [10,11] are currently not supported but could be integrated in future versions of iLAP.

### Case Study

**Figure 4:**
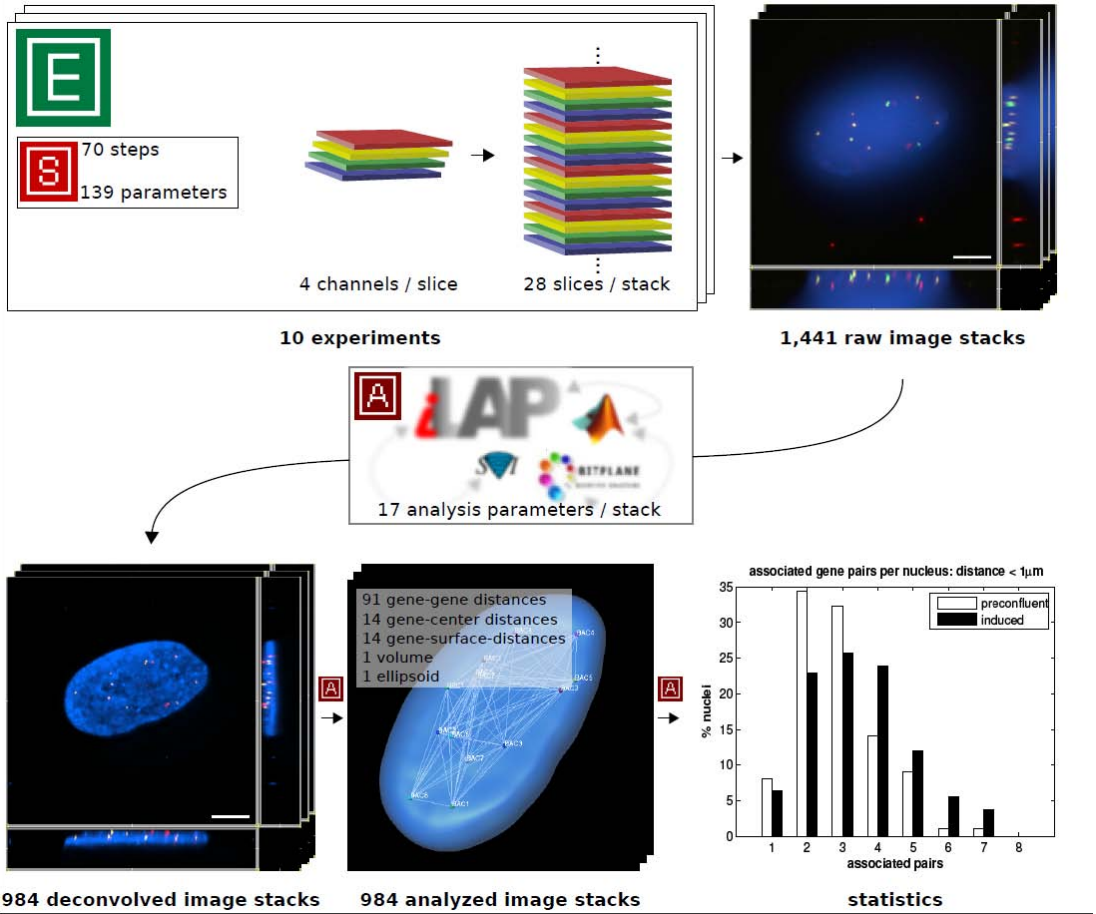
**Case study summary**. The functionality of iLAP was tested in a high-throughput microscopy study. The figure illustrates a summary of the data acquisition and data analysis performed. In 10 experiments a protocol consisting of 70 steps with 139 different parameters was used to generate three-dimensional multicolor image stacks. Each of the 1,441 raw image stacks consisted of 28 optical sections (slices) where each slice was recorded in 4 different channels. The raw image stacks were stored in the iLAP system and thereby connected with the corresponding experiments and protocols. By utilizing the integrated analysis functionality of iLAP the 984 raw images processed by the Huygens 3D-deconvolution package and analyzed by an external semiautomatic procedure implemented in Matlab and Imaris-XT. The analytical pipeline produced data for 121 different distance measurements of each single image. The resulting images and data were then stored in their experimental context within the iLAP system.

In order to test the functionality of the system, we used a high-throughput microscopy study. The focus of this study was on the three dimensional nuclear localization of a group of seven genes. This required the development of a combinatorial multicolor fluorescence in situ hybridization (m-FISH) protocol. This protocol enables simultaneous detection and visualization of all seven genes by using a combination of three different fluorescent labels. The elaboration and optimization of m-FISH required many different protocol steps and parameters. Thus it was crucial to keep a record of any parameter and procedure changes during the process of protocol development. These changes were directly connected with data produced in the lab (e.g. concentration of the FISH probes, probe labeling efficiencies etc.) and the resulting imaging data. In the final combinatorial m-FISH protocol, 70 steps and 139 different parameters were present. Using this protocol we conducted 10 experiments and produced 1,441 multicolor 3D-Image stacks of which 984 were subsequently corrected for color shifts and processed by 3D-deconvolution performing 100 iterations of the maximum likelihood estimation algorithm available with the Huygens Deconvolution Software (Scientific Volume Imaging - SVI http://www.svi.nl). These image processing steps were realized as batch analysis in iLAP, which delegated the compute intensive procedure to a high-performance computing cluster and then stored all processed image stacks in the analysis container of the corresponding experiments. Afterwards FISH signals were detected and analyzed using a custom image analysis procedure which was realized as a Matlab (MathWorks Inc.) extension of Imaris (Bitplane Inc.) using the Imaris-XT programming interface. This extension automatically recorded FISH signal coordinates, signal to signal distances, the nuclear volume and several additional parameters of each imaged nucleus. These externally generated data files were transferred back into iLAP and stored in the context of the corresponding experiment as an external analysis step. A summary of the data acquisition and analysis is shown in Figure 4.

During the course of the study we observed several clear advantages of the iLAP system over a lab-book in paper form, which was maintained in parallel. The first and most valuable feature of iLAP is the direct connection between protocol steps and data files which cannot be realized using a paper lab book. A second notable advantage of the iLAP system was that lab-tasks that were performed in parallel or in overlapping time-frames could also be stored as such, whereas in the traditional lab book all tasks performed in the lab were written sequentially which implied a break-up of connected protocols. A third advantage was that iLAP allowed for rapid searching and finding of experiments, protocols and desired terms, which required only a few mouse clicks as opposite to the cumbersome search using a paper notebook. Moreover, iLAP enabled easy collaboration functionality, data backup or parameter completeness checks.

## Conclusion

We have developed a unique information management system specifically designed to support the creation and management of experimental protocols, and to analyze and share laboratory data. The design of the software was guided by the laboratory workflow and resulted in four unified components accessible through a web interface. The first component allows the hierarchical organization of the experimental data, which is organized in a generic document management system. The second component focuses on protocol development using templates of standard operating procedures. Next, the data acquisition and analysis component offers the possibility to transfer the generated files to a central repository and to associate the files with the corresponding protocol steps. Additionally, external data analysis programs can be integrated and executed on a remote high-performance computing cluster. The last component enables collaboration and data sharing between scientists using iLAP on a user or institutional level as well as protocol transfer with external users.

Although designed in an experimental context for high-throughput protocols like microarray studies of gene expression, DNA-protein binding, proteomics experiments, or high-content image-based screening studies, iLAP has also proven to be valuable in low- and medium-throughput experiments. For example, protocols for qPCR analysis of gene expression using 96 and 384-well formats -a widely used technique- can be easily developed and can contribute significantly to establishment of robust assays. Moreover, since the workflow-oriented concept of iLAP offers the flexibility of a more general scientific data management system it is not limited to a special laboratory protocol, instrument, or type of molecule. For example, its application for next-generation sequencing is straightforward since similar requirements on the computational environment (increasing amount of data, integration of analysis tools, or use of high-performance computing infrastructure) have to be met.

In summary, we have developed a flexible and versatile information management system, which has the potential to close the gap between electronic notebooks and LIMS and can therefore be of great value for a broader community. Extensive tests in our and other labs have shown that the benefits of better information access and data sharing immediately result in reduced time spent managing information, increased productivity, better tracking and oversight of research, and enhanced data quality.

## Availability and requirements

In order to reach a broader audience of users we have implemented a Java-based installer application, which is guiding an inexperienced computer user through the installation process (see Additional file 2). The basic installer package of iLAP has been tested on most common operating systems for which a Java Virtual Machine Version 1.5 or higher is available, e.g. Unix-based systems (Linux, Solaris, etc.), MacOS and Windows and can be downloaded from http://genome.tugraz.at/iLAP/. In addition to the requirement of a Java VM, a PostgreSQL database must be either locally installed or accessible via network. PostgreSQL comes with an easy-to-use installation wizard, so the complete installation should not be a significant entry level barrier. For further information about installation, please read the installation instructions from the download web site and in case of problems please contact the developers under iLAP@genome.tugraz.at. For initial testing purposes, please see also our test environment http://ilapdemo.genome.tugraz.at.

Regarding hardware requirements, the most critical issue is disk space for large data files. These are stored in a directory hierarchy where the base directory must be specified during the installation process. The requirements regarding processor performance and memory depend on the user basis, but PC or server hardware with 2 GB of RAM should be sufficient for most installations.

The production environment for our central in-house installation consists of a 4-processor AMD-system X4600 from Sun Microsystems, with 16 GB of RAM which is connected to an 8TB SAN storage. For computational intensive tasks, iLAP delegates the calculations to a 48-node high-performance computing cluster using the JClusterService interface.

## Abbreviations

JavaEE: Java Enterprise Edition platform; MDA: Model Driven Architecture; OMERO: Open Microscopy Environment Remote Objects; SOAP: Simple Object Access Protocol; FuGE: Data standard for Functional Genomic Experiment.

## Authors' contributions

The conceptual idea for iLAP goes back to GS, JGM and ZT and was elaborated by GS in the course of the GENAU-mobility/NIH-visiting-scientist program. GS and MF performed the implementation of the main software modules including persistence-, business- and web tier. SK implemented the data retrieval functionality and worked also on the integration of OMERO. GB together with GS was responsible for the Java-Applet-based file transfer functionality which was additionally extended to work as an ImageJ-Plugin. Archiving functionality and the XML export for general experiment protocol sharing was implemented by MO. DR contributed with extremely useful comments about conceptual ideas, their practical application and their usability. DR's constant input derived from permanent testing under real work conditions lead to major improvements in functionality, usability and responsiveness. The project was coordinated by GS.

## Supplementary Material

Additional file 1 **iLAP user manual**. The iLAP user manual contains a detailed description of the user interface including screen shots. Click here for file

Additional file 2 **iLAP installation and administration manual**. The iLAP installation and administration manual contains a detailed description of the installation process for all supported platforms including screen shots. Click here for file

Additional file 3 **m-FISH protocol in PDF format**. This file contains the combinatorial multiple fluorescence in situ hybridization (m-FISH) protocol in PDF format used in the case study section. Click here for file

Additional file 4 **m-FISH protocol in XML format**. This file contains the combinatorial multiple fluorescence in situ hybridization (m-FISH) protocol in XML format used in the case study section for protocol exchange. Click here for file

Additional file 5 **Document Type Definition for the XML protocol format**. This file contains the Document Type Definition for the XML format used for protocol exchange created in collaboration with Will Moore from the OMERO.editor project. Click here for file

## References

[B1] Acevedo LG Iniguez AL Holster HL Zhang X Green R Farnham PJ Genome-scale ChIP-chip analysis using 10,000 human cells Biotechniques 2007 43 791 797 10.2144/000112625 18251256 PMC2268896

[B2] Piggee C LIMS and the art of MS proteomics Anal Chem 2008 80 4801 4806 10.1021/ac0861329 18609747

[B3] Haquin S Oeuillet E Pajon A Harris M Jones AT van Tilbeurgh H Data management in structural genomics: an overview Methods Mol Biol 2008 426 49 79 full_text 18542857 10.1007/978-1-60327-058-8_4

[B4] Maurer M Molidor R Sturn A Hartler J Hackl H Stocker G MARS: microarray analysis, retrieval, and storage system BMC Bioinformatics 2005 6 101 10.1186/1471-2105-6-101 15836795 PMC1090551

[B5] Saal LH Troein C Vallon-Christersson J Gruvberger S Borg A Peterson C BioArray Software Environment (BASE): a platform for comprehensive management and analysis of microarray data Genome Biol 2002 3 SOFTWARE0003 10.1186/gb-2002-3-8-software0003 12186655 PMC139402

[B6] Hartler J Thallinger GG Stocker G Sturn A Burkard TR Korner E MASPECTRAS: a platform for management and analysis of proteomics LC-MS/MS data BMC Bioinformatics 2007 8 197 10.1186/1471-2105-8-197 17567892 PMC1906842

[B7] Craig R Cortens JP Beavis RC Open source system for analyzing, validating, and storing protein identification data J Proteome Res 2004 3 1234 1242 10.1021/pr049882h 15595733

[B8] Rauch A Bellew M Eng J Fitzgibbon M Holzman T Hussey P Computational Proteomics Analysis System (CPAS): an extensible, open-source analytic system for evaluating and publishing proteomic data and high throughput biological experiments J Proteome Res 2006 5 112 121 10.1021/pr0503533 16396501

[B9] Moore J Allan C Burel JM Loranger B MacDonald D Monk J Open tools for storage and management of quantitative image data Methods Cell Biol 2008 85 555 570 full_text 18155479 10.1016/S0091-679X(08)85024-8

[B10] Jones AR Pizarro A Spellman P Miller M FuGE: Functional Genomics Experiment Object Model OMICS 2006 10 179 184 10.1089/omi.2006.10.179 16901224

[B11] Jones AR Miller M Aebersold R Apweiler R Ball CA Brazma A The Functional Genomics Experiment model (FuGE): an extensible framework for standards in functional genomics Nat Biotechnol 2007 25 1127 1133 10.1038/nbt1347 17921998

[B12] Brazma A Hingamp P Quackenbush J Sherlock G Spellman P Stoeckert C Minimum information about a microarray experiment (MIAME)-toward standards for microarray data Nat Genet 2001 29 365 371 10.1038/ng1201-365 11726920

[B13] Taylor CF Paton NW Lilley KS Binz PA Julian RK Jr Jones AR The minimum information about a proteomics experiment (MIAPE) Nat Biotechnol 2007 25 887 893 10.1038/nbt1329 17687369

[B14] Deutsch EW Ball CA Berman JJ Bova GS Brazma A Bumgarner RE Minimum information specification for in situ hybridization and immunohistochemistry experiments (MISFISHIE) Nat Biotechnol 2008 26 305 312 10.1038/nbt1391 18327244 PMC4367930

[B15] Drake DJ ELN implementation challenges Drug Discov Today 2007 12 647 649 10.1016/j.drudis.2007.06.010 17706546

[B16] Taylor KT The status of electronic laboratory notebooks for chemistry and biology Curr Opin Drug Discov Devel 2006 9 348 353 16729731

[B17] Butler D Electronic notebooks: a new leaf Nature 2005 436 20 21 10.1038/436020a 16001034

[B18] Kihlen M Electronic lab notebooks - do they work in reality? Drug Discov Today 2005 10 1205 1207 10.1016/S1359-6446(05)03576-2 16213407

[B19] Bradley J-C Samuel B SMIRP-A Systems Approach to Laboratory Automation Journal of the Association for Laboratory Automation 2004 5 48 53 10.1016/S1535-5535(04)00074-7

[B20] Apache Software Foundation Tapestry web frame work 2009 http://tapestry.apache.org/

[B21] Apache Software Foundation Java implementation of the SOAP ("Simple Object Access Protocol") 2009 http://ws.apache.org/axis/

[B22] Krug S Don't make me think! A Common Sense Apporach to Web Usability 2000 Indianapolis, Indiana, USA: New Riders Publishing

[B23] Johnson J Web Bloopers: 60 Common Web Design Mistakes and How to Avoid Them 2003 San Francisco, CA, USA: Morgan Kaufmann Publishers Inc

[B24] SpringSource Spring lightweight application container 2009 http://www.springsource.org/

[B25] Apache Software Foundation Services and configuration microkernel 2009 http://hivemind.apache.org/

[B26] Apache Software Foundation Apache servlet container 2009 http://tomcat.apache.org/

[B27] Johnson R Hoeller J Expert One-on-One J2EE Development without EJB. Wrox 2004

[B28] OW2 Consortium Java Open Transaction Manager (JOTM) 2009 http://jotm.ow2.org/xwiki/bin/view/Main/WebHome?

[B29] Bohlen M AndroMDA 2009 http://www.andromda.org/19041892

[B30] Rasband WS ImageJ 2009 http://rsb.info.nih.gov/ij/

[B31] Abramoff MD Magelhaes PJ Ram SJ Image Processing with ImageJ Biophotonics International 2004 11 36 42

[B32] Roure D Goble C Bhagat J Cruickshank D Goderis A Michaelides D myExperiment: Defining the Social Virtual Research Environment 182 189

