# Supplementary material for: iLAP: a workflow-driven software for experimental protocol development, data acquisition and analysis
Source: BMC Bioinformatics. 2009 Nov 26;10:390. doi: 10.1186/1471-2105-10-390 (PMC2789074; doi:10.1186/1471-2105-10-390)
Supplement: Additional file 1 — iLAP user manual. The iLAP user manual contains a detailed description of the user interface including screen shots. [file 1471-2105-10-390-S1.PDF]

LABORATORY DATA MANAGEMENT SYSTEM, ANALYSIS- AND PROTOCOL DEVELOPMENT

USER MANUAL

(Version 1.3.2)

Authors:

Gernot Stocker, gernot.stocker@tugraz.at

Maria Fischer, maria.fischer@tugraz.at

Simon Kainz, simon.kainz@tugraz.at

Graz University of Technology
Institute for Genomics and Bioinformatics
Petersgasse 14, 8010 Graz, Austria

Contents

1 Introduction . . . 1

1.1 Abstract . . . 2
1.2 Motivation . . . 3
1.2.1 Definition . . . 3
1.2.2 Data acquisistion . . . 4
1.2.3 Data analysis . . . 4
1.2.4 Data retrieval . . . 5

2 iLAP core modules . . . 6

2.1 Overview . . . 7
2.1.1 Header . . . 7
2.1.2 Quick navigation menu . . . 7
2.1.3 Main overview . . . 8
2.1.4 Color code and common symbols . . . 9
2.2 Digital lab book . . . 10
2.2.1 Basic layout . . . 10
2.2.2 Project . . . 10
2.2.3 Experiment . . . 11
2.2.4 Current Working Protocol (CWP) . . . 11
2.2.5 Standard Protocol (SOP) . . . 17
2.2.6 Sharing . . . 18
2.3 Data acquisition . . . 19
2.3.1 Notes . . . 19
2.3.2 Single file upload . . . 19
2.3.3 File upload via applet . . . 20
2.3.4 Experiment wizard . . . 21
2.4 Data analysis . . . 24
2.4.1 External analysis steps . . . 25
2.4.2 Internal analysis steps . . . 26
2.4.3 Details . . . 26
2.5 Data retrieval . . . 28
2.5.1 Project search . . . 28
2.5.2 Experiment search . . . 28

2.5.3 Note search 29

3 Software development 30

3.1 Analysis module development 31
3.2 SOAP interface for external access 33

A Bibliography 35

B Glossary 38

C Acknowledgments 41

Chapter 1

Introduction

1.1 Abstract

Both quantitative and high-throughput microscopy confront scientists with the daunting challenge of storing heterogeneous data in a structured and organized way. Among the available data management solutions there is no intuitively usable system which covers the complete work flow of microscopy experiments. Therefore we have developed the web application iLAP (Laboratory data management, Analysis and Protocol development)iLAP [2008] which combines experimental protocol development, wizard-based data acquisition and high-throughput data analysis into one single, integrated system.

A unique feature of iLAP is that it supports the experimental design phase. The working protocols that scientists develop for wet lab experiments can be stored and modified within iLAP. Eventually, these protocols lead to the acquisition of image data. iLAP then provides a data acquisition wizard to assist the scientist in associating the acquired image data with steps in the protocol. Standardization initiatives, such as MISFISHIE Deutsch et al. [2008] for DNA fluorescence in situ hybridization, can be supported by enforcing the input of required parameters during this data acquisition. Further processing and analysis of the raw image data can be performed either with analysis modules developed specifically within iLAP or with external third party programs. Interesting visual observations can be documented as notes at any place.

iLAP has been realized as a standard three tier business application using state-of-the-art JEE (Java Platform, Enterprise Edition 5) software technologies. Due to this flexible software architecture we were able to develop an intuitive web interface as well as a generic SOAP (Simple Object Access Protocol)- based programming interface. This platform independent programming interface allows external image processing applications like ImageJ Abramoff [2004] to access the files stored in iLAP. Computationally intensive tasks like three-dimensional deconvolution of image stacks McNally et al. [1999] are transparently distributed on high-performance computing infrastructure, and results are stored within the underlying relational database management system. These results are associated automatically with the original experiment data. To facilitate ease of use, this complexity of data management and IT infrastructure is completely hidden from the user.

iLAP is an open source project which is freely available for the microscopy community. It has been tested extensively in our in-house microscopy facility and continuous user feedback has led to improvements in functionality, usability and responsiveness. A distinctive virtue of iLAP is that it inherently leads to structured recording of experiments, conserving the complete experimental context of data records throughout the history of the research project.

1.2 Motivation

iLAP has been desinged to support scientists through all phases of experimental work. In order to define software requirements, the lab work and its different stages have been analyzed to establish a complete workflow oriented model. Observations suggest that the lab work can be divided into four principal steps:

1. project definition
2. data acquisition
3. data analysis and processing
4. data retrieval

These four steps explain how iLAP can be used to track data evolution in an experimental stage.

1.2.1 Definition

In general, scientists start by asking biological questions, to which the answers are of special interest but largely unknown. A detailed literature research should determine whether others have already satisfactorily solved the specific questions. If not, the next stage is to set up a hypotheses that can be tested and thus proven or disproven. Starting from such considerations, a new project should be defined and captured in digital form. It is necessary to describe the new project by: giving it a descriptive name; defining which questions should be answered and establishing what the solution strategy should look like. Documents collected during the literature research stage should be collated with the project definition for later review or for sharing with other researchers.

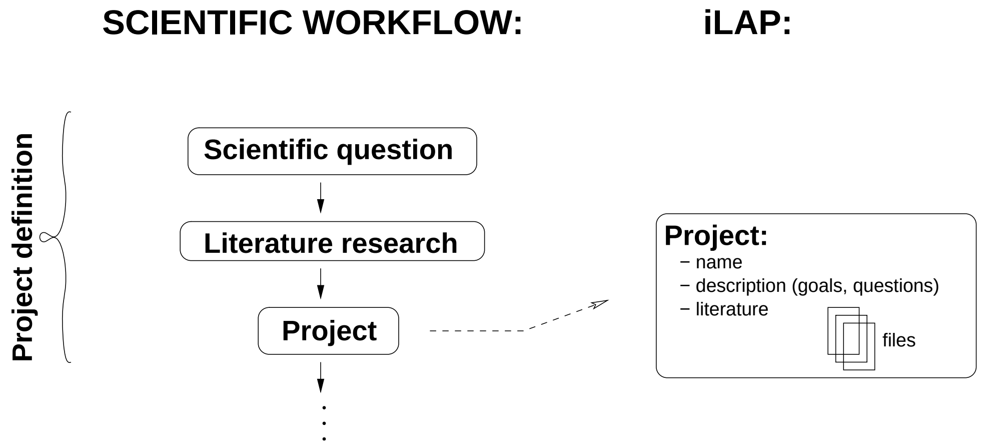

1.2.2 Data acquisistion

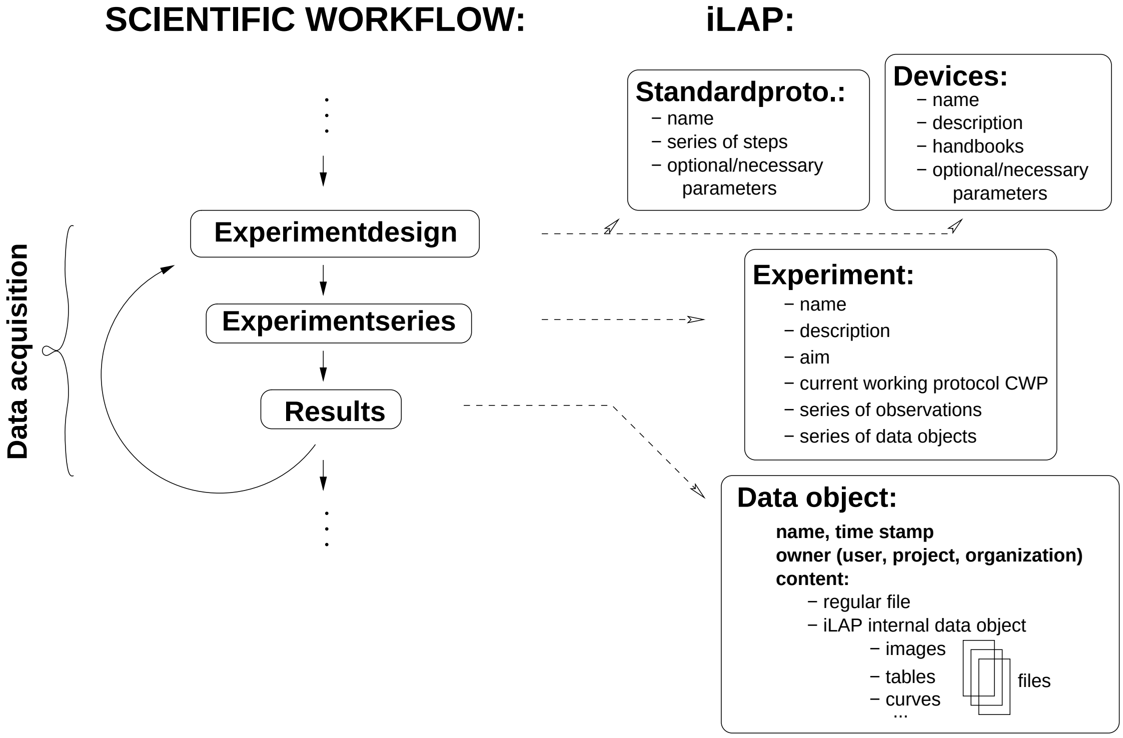

1.2.3 Data analysis

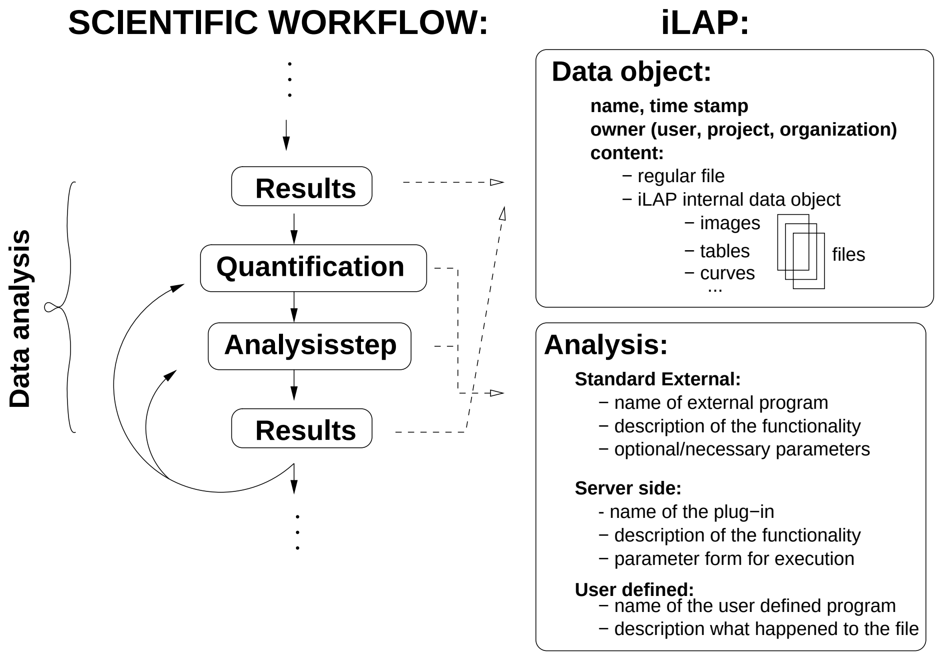

1.2.4 Data retrieval

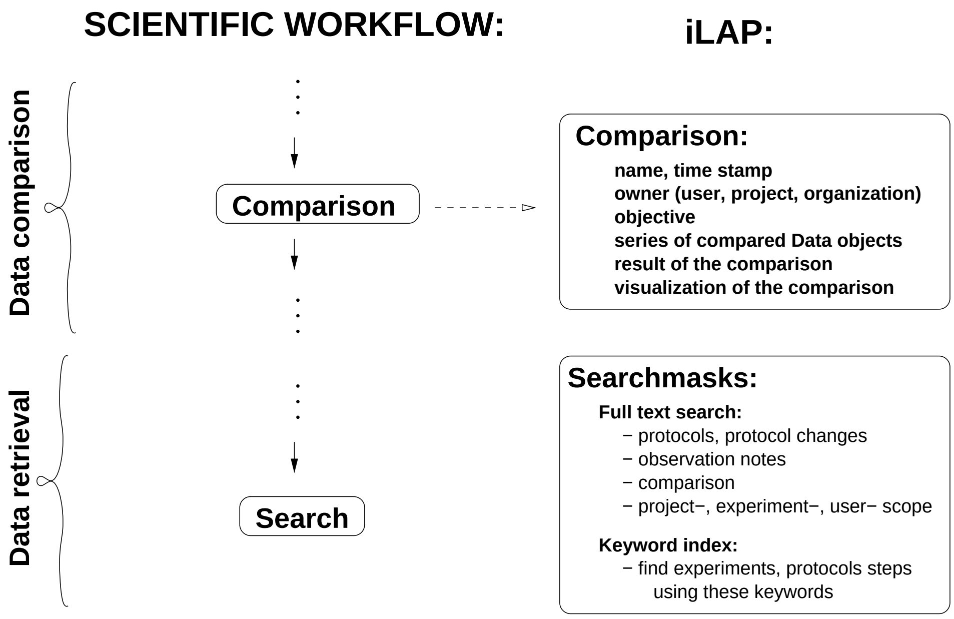

Chapter 2

iLAP core modules

2.1 Overview

This section provides information about iLAP's principal page layout including page structure, color and icon convention. After successfully logging in the user is redirected to the main overview shown in figure 2.2. iLAP's basic layout can be divided into two sections:

- header and quick navigation, and
- page specific content

2.1.1 Header

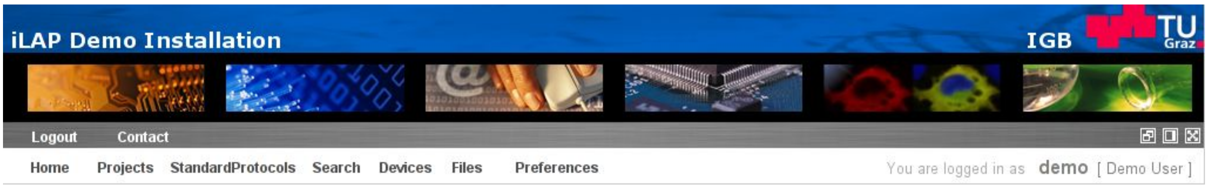

Figure 2.1: iLAP header and quick navigation

The header consists of three areas: some images on the top, a bar managing display settings and user authentication/authorization, and a bar containing the quick navigation menu and user information. Note that the quick navigation menu is only available for logged in users. The user can choose between three available display modes by clicking on the corresponding icons at the right side of the header:

- resizes the window to the default size (1024x768)
- resizes the window to the default size (1024x768) and the images at the header section are not shown
- stretches the window to the full width of the screen and the images at the header section are not shown

2.1.2 Quick navigation menu

The quick navigation menu at the top section offers the user to

- navigate to the main overview
- create a new project or display the user's accessable projects
- create a new standard protocol or display the user's accessable standard protocols

- search through projects, experiments, or notes
- create a new device or display the user's accessable devices
- start the multiple file uploader applet
- configure the user's preferences (including display settings)

2.1.3 Main overview

To facilitate the work within iLAP the user is redirected to the main overview after each login. The overview is divided into two components. The area at the right side holds a project tree displaying the user's owned and shared projects. The tree further lists a project's attached experiments, subprojects, analyses, and files in a hierarchical way. When clicking on tree elements the section on the left side, providing and enabling content specific information and actions, is updated.

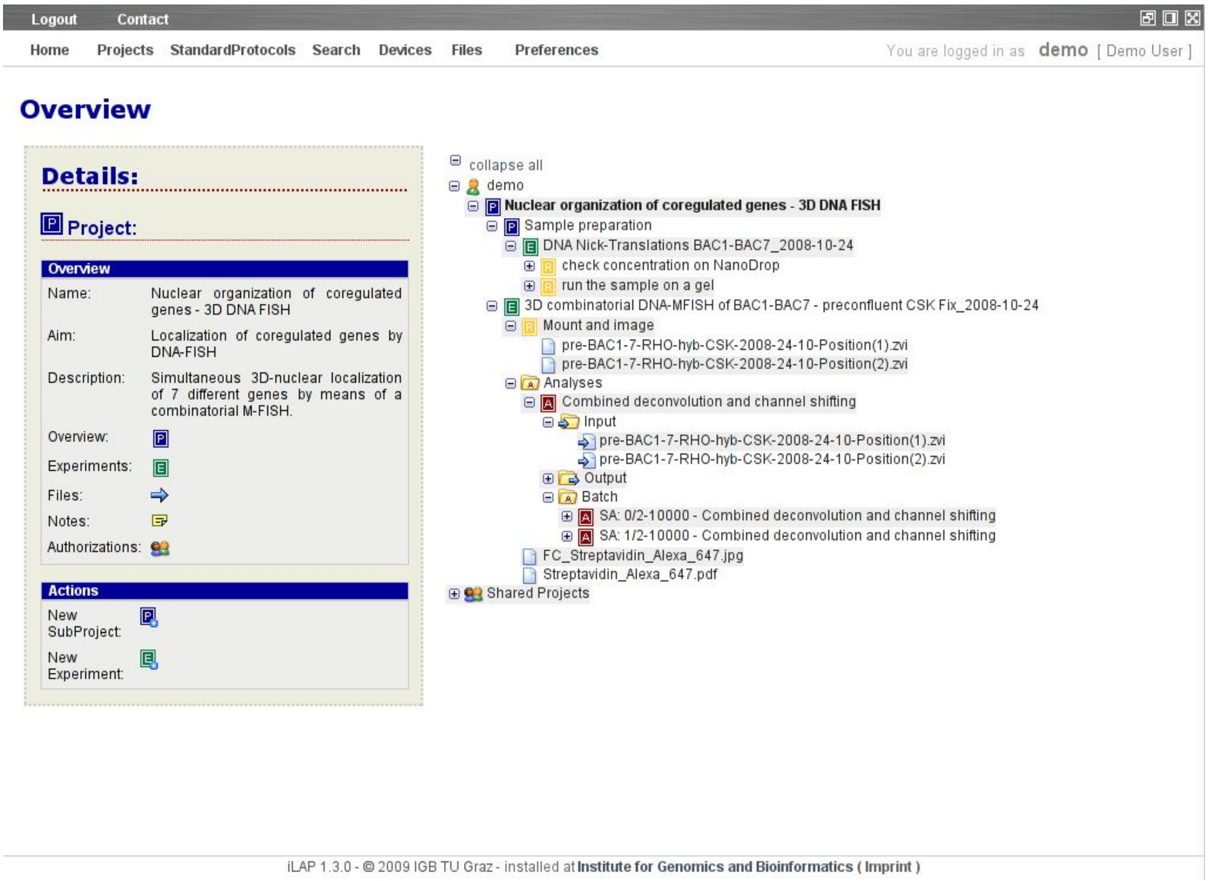

Figure 2.2: overview page showing a selected project's information

2.1.4 Color code and common symbols

iLAP's content is structured in and associated with four main themes: projects, experiments, standard protocols, and analyses. To organize the application colors and symbols are assigned to each theme:

theme	color	symbol
project	blue	P
experiment	green	E
standard protocol	red	S
analysis	dark red	A

2.2 Digital lab book

The digital lab book handles and supports data management which needs to be done during the experimental design phase. It helps scientists to retain structure in their daily lab work by organizing experiments in a project oriented way. This section describes the digital lab book's main functions in more detail.

2.2.1 Basic layout

To ensure a consistent design each page belonging to the digital lab book is structured in the following way: in the top area breadcrumbs and a headline indicating the category to which the page's content is related to are provided. For a better orientation and navigation the breadcrumbs denote the (sub-)project and experiment the user is currently working on. If useful a tab panel is provided in the mean area to further structure the selected information. By clicking on a tab the according page will be loaded.

2.2.2 Project

Projects are one of the basic organizational units and act as a container for subprojects and experiments. A project consists of name, short description, aim, description, owner, submitter, and date of creation. In order to define a project it is sufficient to declare the project's name - short description, aim, and description is seen as optional information whereas owner, submitter as well as date of creation are captured by the system automatically. Figure 2.3 shows a project's general overview which offers editing and deleting functionality. The other tabs provide the user with the following information/functionalities: attached subprojects, mapped experiments, attached files, attached notes and project authorizations.

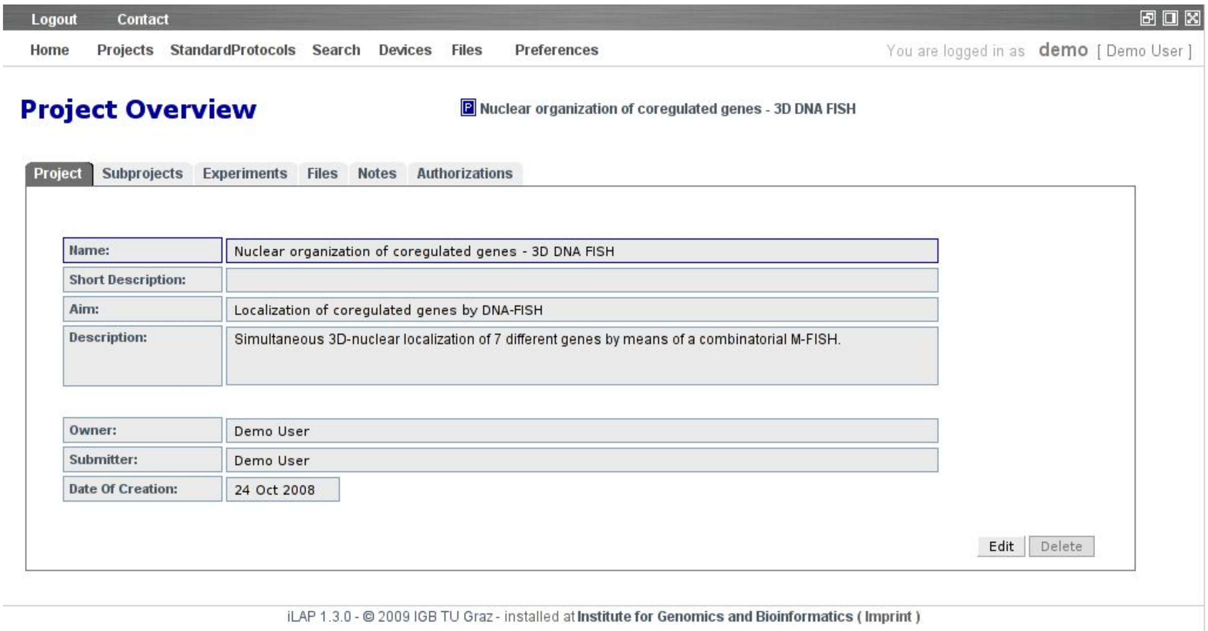

Figure 2.3: general project overview

2.2.3 Experiment

Experiments map real world experiments in iLAP. Therefore an experiment's name, question, description, owner, submitter, date of creation, and its current working protocol (for details see section 2.2.4) are stored. Again, in order to create an experiment the only attribute which must be defined is the experiment's name. Everything else (except the experiment's current working protocol) is either optional information or recorded automatically by iLAP.

Besides CWP information the user has access to an experiment's general overview (including deletion and edition functionality), attached files and notes, as well as its performed analyses (for detailed information about data analysis refer to 2.4). It is worth mentioning that experiments and respectively its owning projects may just be deleted as long as no additional information (in form of files) is attached to them.

2.2.4 Current Working Protocol (CWP)

A CWP symbolizes the working step sequence which has to be followed in order to conduct an experiment. To define this step sequence iLAP supports five different approaches:

- step and parameter creation from scratch
- copy and paste steps from other CWPs or SOPs
- import a SOP

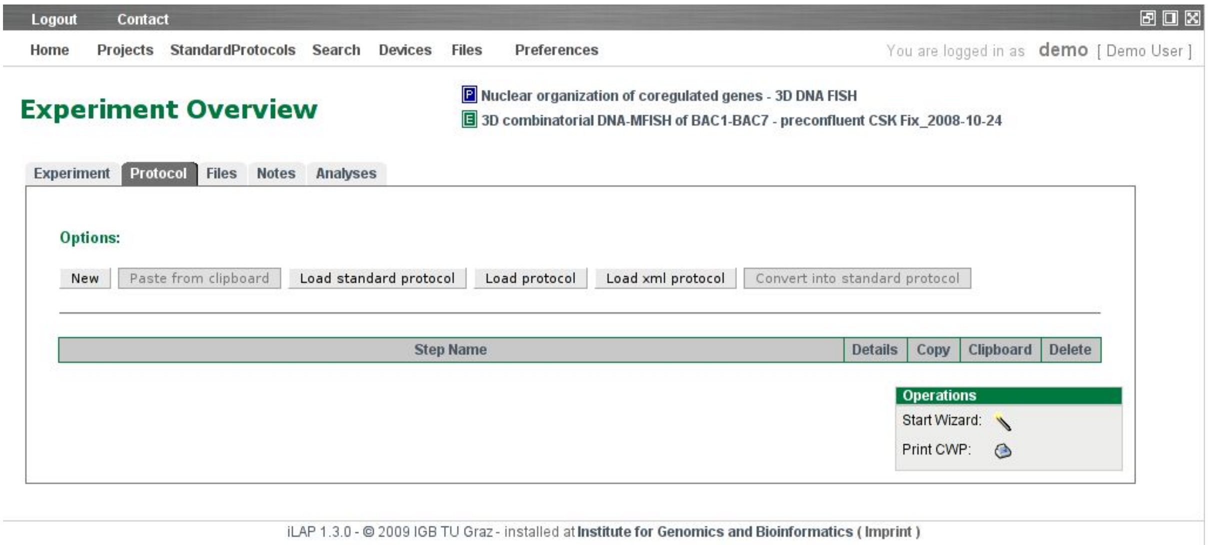

Figure 2.4: experiment: protocol tab showing an empty CWP

- copy another experiment's CWP
- load a previously exported CWP (in XML format)

It is natural that after loading steps or an entire protocol, the currently created CWP is adjusted to meet the experiment's needs. Figure 2.4 shows an empty CWP and its provided options (step creation, clipboard, standard protocol import, protocol loading, xml import). Notice that the conversion functionality is not available as no CWP steps are yet defined to be converted into a standard protocol. If no protocol steps have been added to the clipboard in the current session, the clipboard functionality is also disabled.

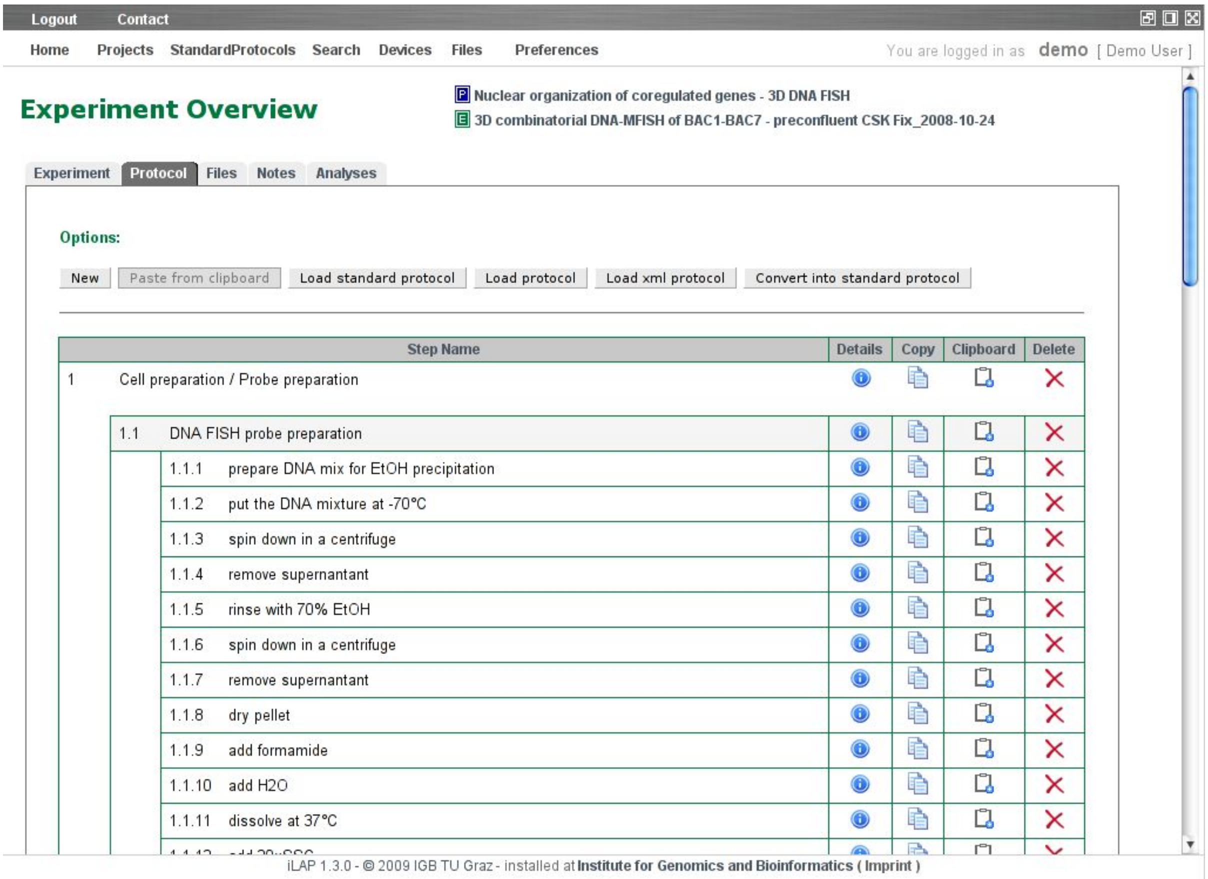

Figure 2.5: experiment: protocol tab showing an already defined CWP

2.2.4.1 Single step creation

To define a CWP from scratch each step can be created by hand. Basically iLAP distinguishes between three different types of steps:

- **step:** symbolizes a simple step which needs to be conducted during an experiment.
- **step group:** steps that are content-related can be grouped into this step type.
- **split step:** sometimes two or more step groups are meant to be carried out simultaneously. Therefore this step type is offered. Notice that only step groups are allowed to be declared in split steps.

Similarly to projects and experiments iLAP provides a CWP step's general information (see figure 2.6). Besides the functionality to edit, delete or navigate through the steps this page also offers options to specify and delete parameters and (if allowed) to add and delete further steps.

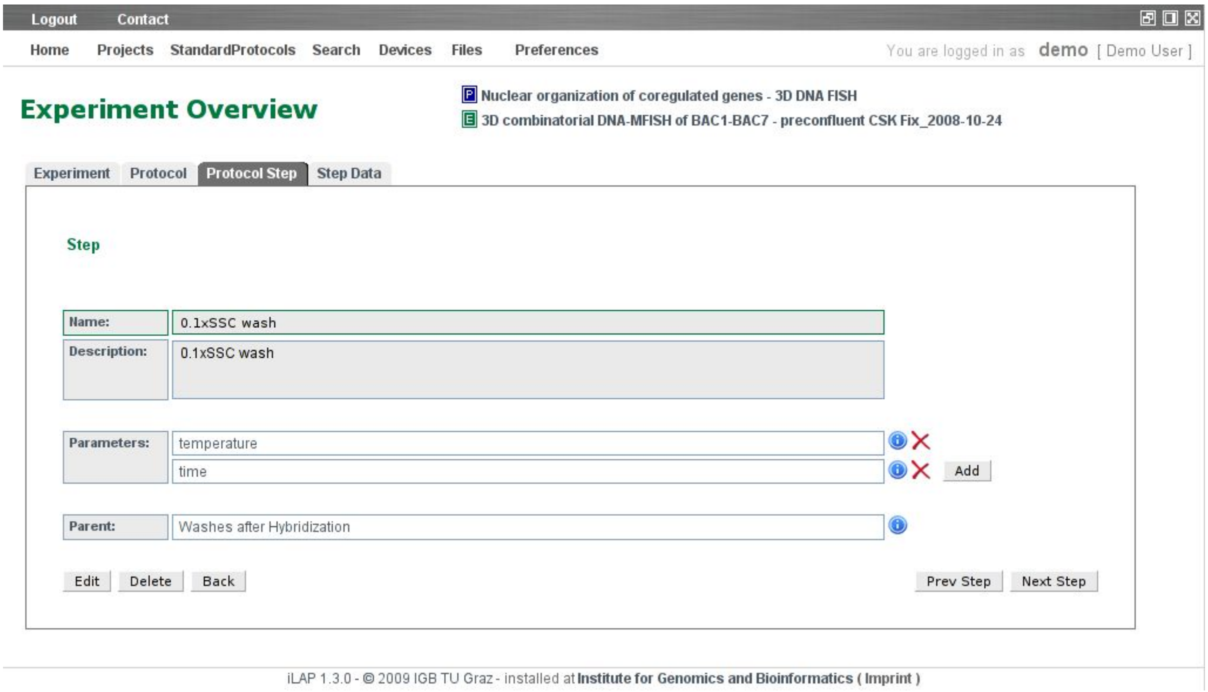

Figure 2.6: general overview of a simple CWP step

2.2.4.2 Parameter

Parameters contain additional information about steps, therefore they must be associated to a particular (CWP or SOP) step. The attributes name and description describe the parameter. Optionally a default value, a parameter's necessity, and user defined units can be specified. During data acquisition the user is forced to enter each required parameter whereas it is left to the user to decide which optional parameter should be declared as well. In General three different types of parameter are supported: numerical, string, and enumeration. Figure 2.7 shows an enumeration parameter's general overview in edit mode.

2.2.4.3 Clipboard

While exploring protocols (CWPs as well as SOPs) the user can copy single steps to a clipboard for later pasting into other protocols. Notice that the clipboard remembers its contents only for one session. After a log out the clipboard is cleared.

2.2.4.4 Standard Protocol import

Owned as well as shared (see section 2.2.6) SOPs can be loaded into CWPs. By using the load standard protocol function the user can specify exactly where (before which step) the chosen SOP should be imported and wether the SOP should be loaded into a separate group.

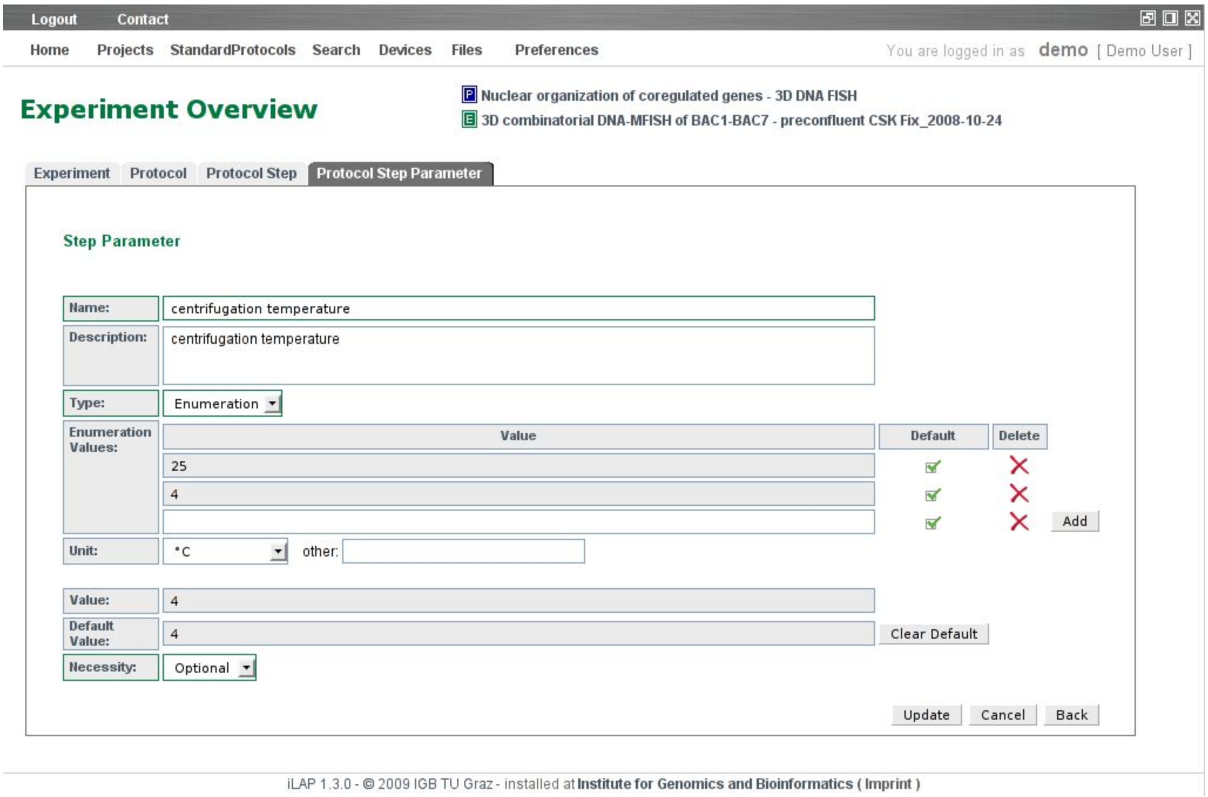

Figure 2.7: overview showing a parameter's details

2.2.4.5 Copy of CWP from existing experiment

Similar to importing SOPs iLAP offers the functionality to load already existing CWPs. Again one must specify which CWP should be loaded at which point. To facilitate finding the right CWP two selection boxes, displaying projects and its experiments are provided.

2.2.4.6 XML import

Besides handling internal collaborations within iLAP via authorizations (see section 2.2.6 for further details) iLAP supports external collaborations by providing an XML import/export function for protocols. The created XML files conform to the common protocol exchange format (.cpe.xml) which has been designed in collaboration with developers of the OMERO.editor .(OMERO [2009])

2.2.4.7 CWP printing

After defining a CWP scientists carry out the experiment in real life. To be independent from computer access in wet labs iLAP supports the export of CWPs to PDF. The exported document shows each step in its correct order including information about a step's parameters and notes. Figure 2.8 gives an example how the printed version of a CWP looks.

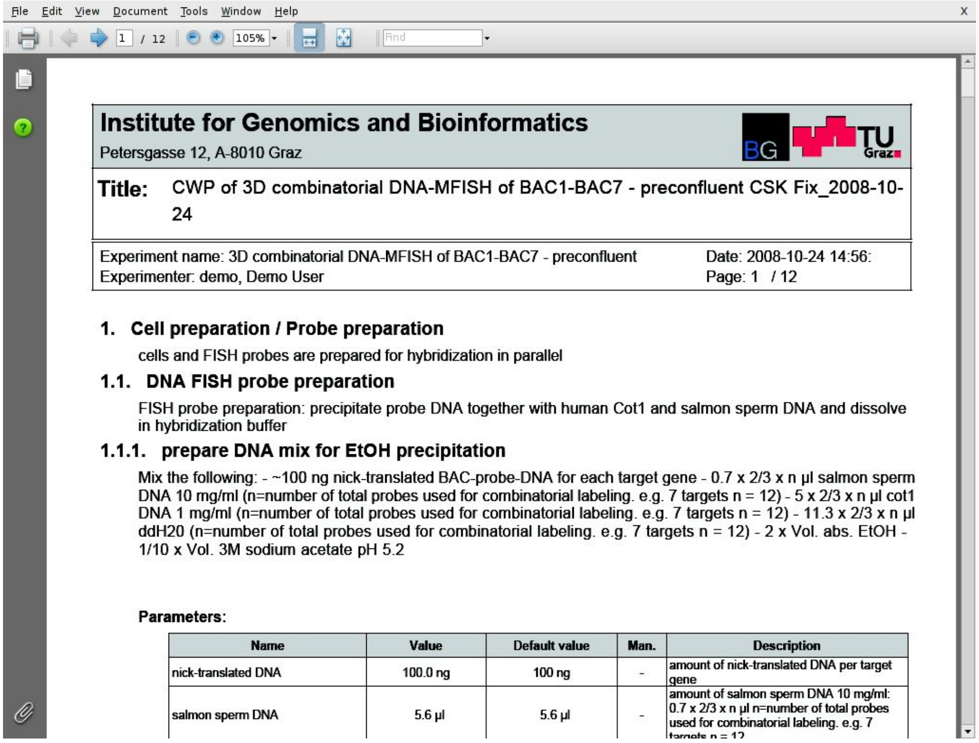

Figure 2.8: generated PDF

2.2.5 Standard Protocol (SOP)

SOPs represent well established protocols which should be used in several experiments. SOPs can be created by either defining them from scratch, importing another SOP from third parties or converting an already defined CWP into a SOP. Since all ways are similar to the creation of CWPs please refere to section 2.2.4 for more details. Figure 2.9 represents an arbitrary SOP overview.

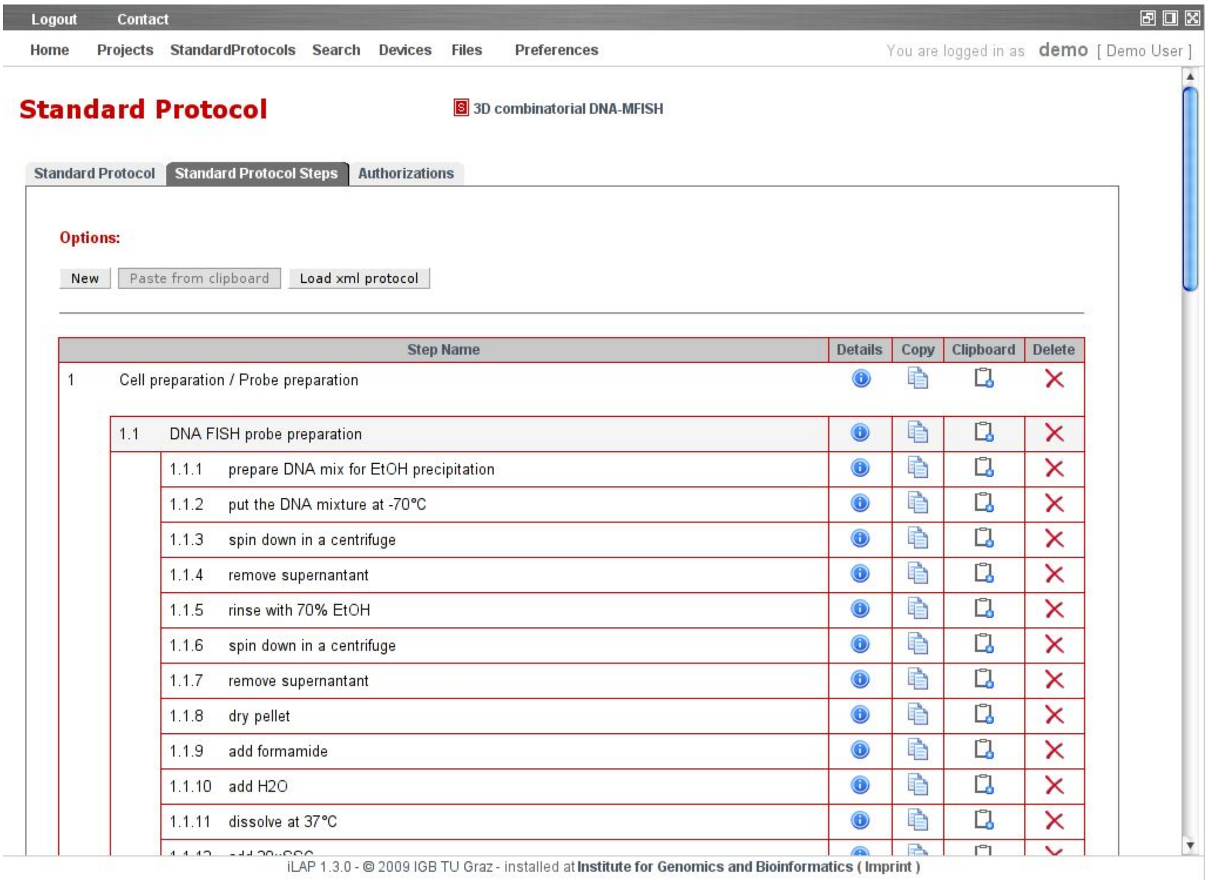

Figure 2.9: SOP overview

2.2.6 Sharing

iLAP allows collaborations on two points: top level projects and SOPs. To grant other users or entire groups access iLAP introduced authorization tabs on project and SOP basis. The protocol's/SOP's owner is provided control (granting or declining) of other user's protocol/SOP access.

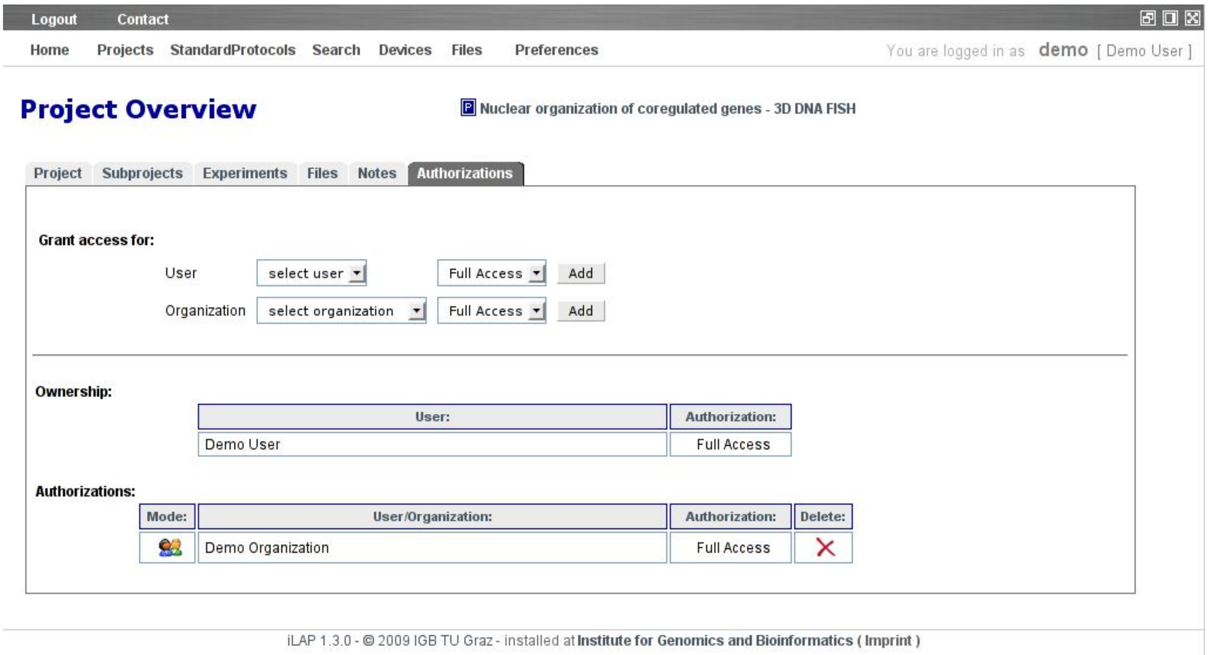

Figure 2.10: page handling a project's authorized users and groups.

2.3 Data acquisition

Data is collected and generated at many stages throughout the course of a project. iLAP handles the storage and organization of different types of data (notes, files, and parameters) in a centralized way. This section describes how to store data within iLAP.

2.3.1 Notes

Observations of interest can be documented as notes at several stages throughout a project. To ensure documentation at any point notes can be assigned to projects, experiments, CWP steps, or analyses depending on the note's content. In general notes can be created and altered by using the entity's note tab to which the note is attached (e.g. figure 2.11). Only notes reporting events regarding CWP steps need to be inserted via the experiment wizard (see section 2.3.4).

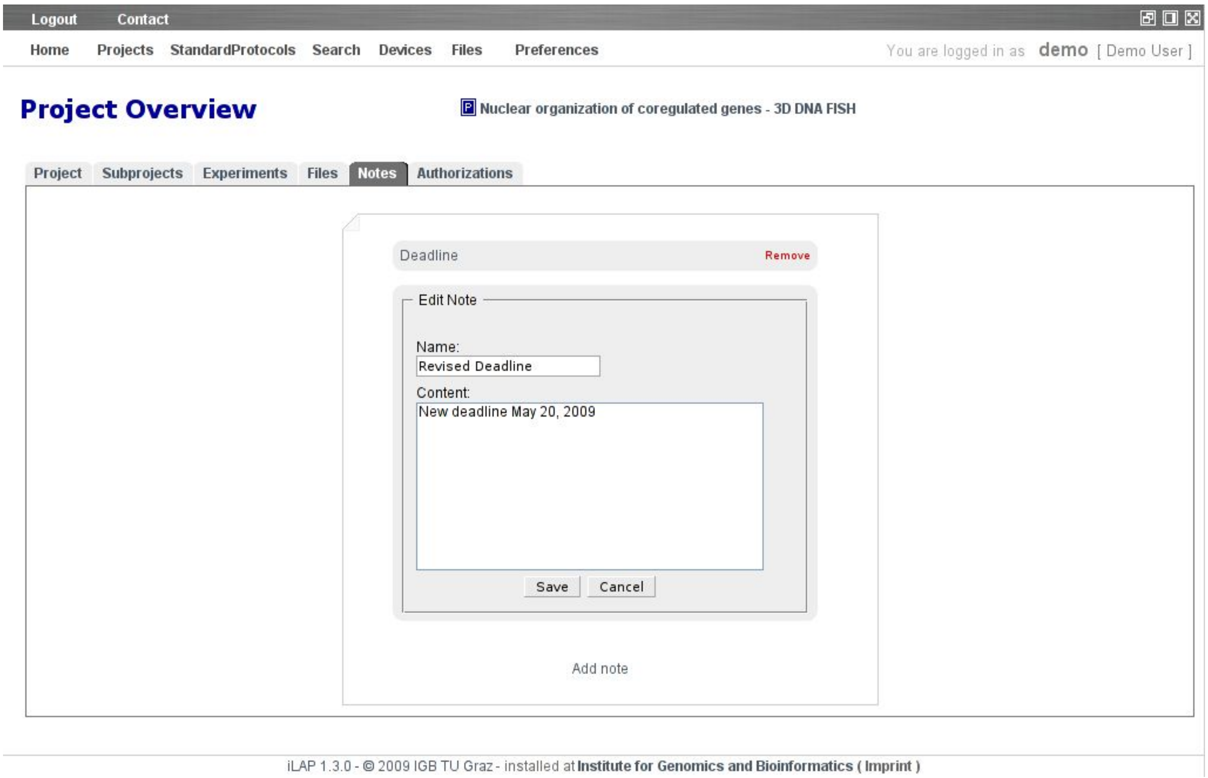

Figure 2.11: project note tab

2.3.2 Single file upload

Besides organizing notes iLAP is capable of storing files in a central repository. The link to a file's associated entity is done automatically by the system. To specify this association the user

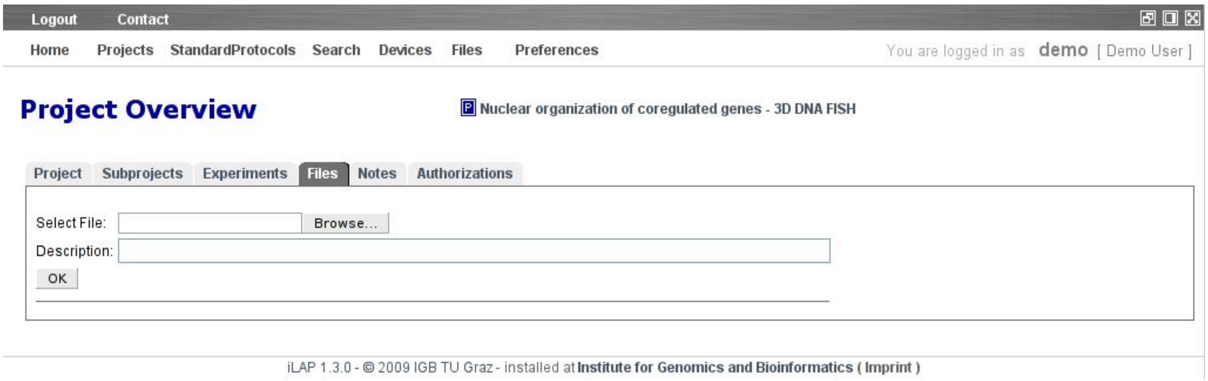

Figure 2.12: project single file upload

needs to navigate to the corresponding file tab. File uploads are available for - and therefore documents are attachable to - projects, experiments, and external analyses. To further characterize imported files an optional document description can be added at each upload. It's understood that files acquired throughout the course of an experiment can be documented as well. Therefore the experiment wizard offers at each CWP step an interface handling data acquisition. Notice that files directly connected to a CWP step are considered as raw files. Once raw files are attached it is no longer possible to delete the entire CWP the document is referring to. This ensures that no raw data will be lost once transferred to the system.

2.3.3 File upload via applet

Often there is the need to upload multiple files at once. This may happen when for example several papers relevant for a project or multiple results of an external analysis should be recorded within iLAP. Since single file upload is not capable of this application, a Java applet for multiple file transfer was integrated into the system.

The applet is available via the link Multiple File Uploader provided in the folder Files on the quick navigation menu. Based on the concept and design of the overview page (described in section 2.1.3) the multiple file uploader displays a user's accessible projects and experiments organized in a tree like manner. As soon as at least one file is attached to a CWP step or an analysis folders to represent this directory are created and shown in the tree as well. Besides file upload multiple file download is supported by providing a download function. A refresh link to update the directory tree ensures that newly created experiments and projects are loaded and displayed in the tree as well.

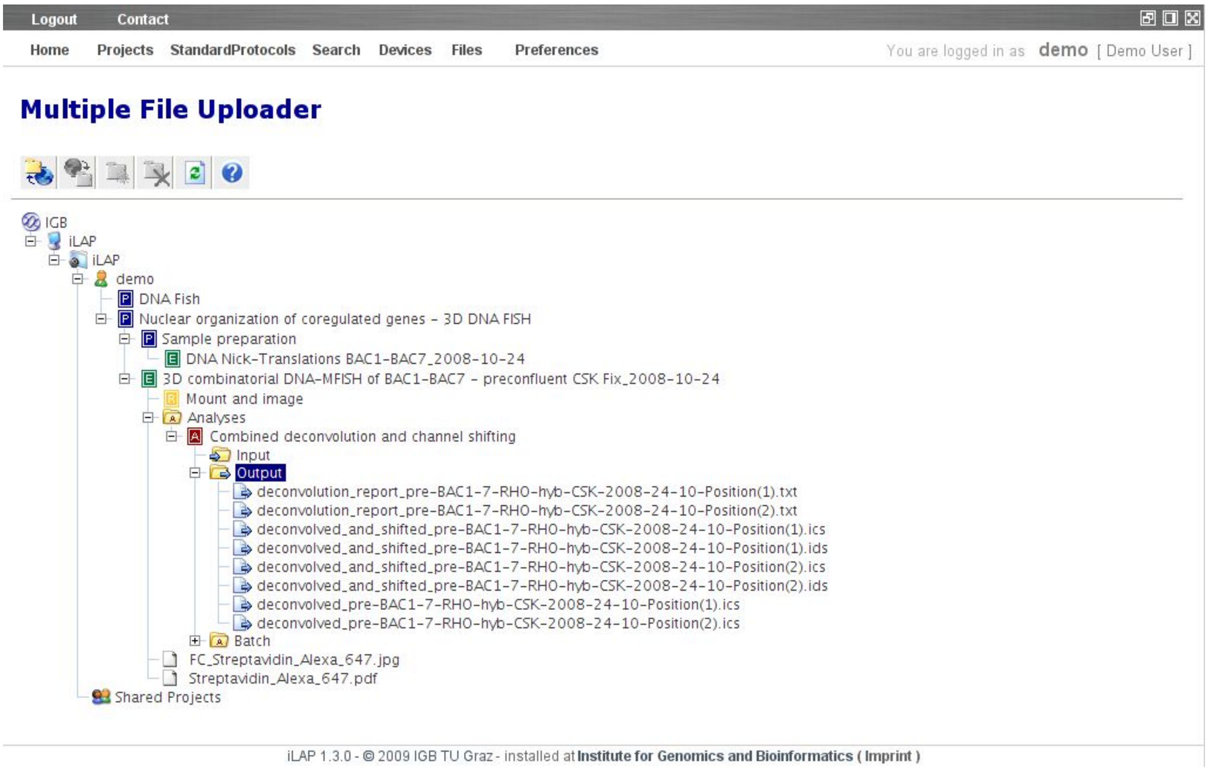

Figure 2.13: Java applet for multiple file transfer

2.3.4 Experiment wizard

As iLAP's purpose is to manage and store all experimental related data the need for a wizard easing data entry is given. A sequence of input masks (shown in figures 2.14 and 2.15) guides through the CWP. Generally the wizard is organized in five sections:

- **navigation panel** provides buttons to navigate to the previous, next, and first step of the CWP as well as functions to save & exit, and exit the wizard
- **overview panel** lists all CWP steps defined and highlights the currently displayed step.
- **step information panel** shows the current step's name, description and attached parameters and offers input fields to specify their corresponding values. If predefined default values exist the wizard will suggest them as the parameter's value.
- **file panel** list all files which are already attached to a CWP step and provides a single file upload area for further file transfers.
- **note panel** handles the creation, deletion and display of notes concerning the current CWP step.

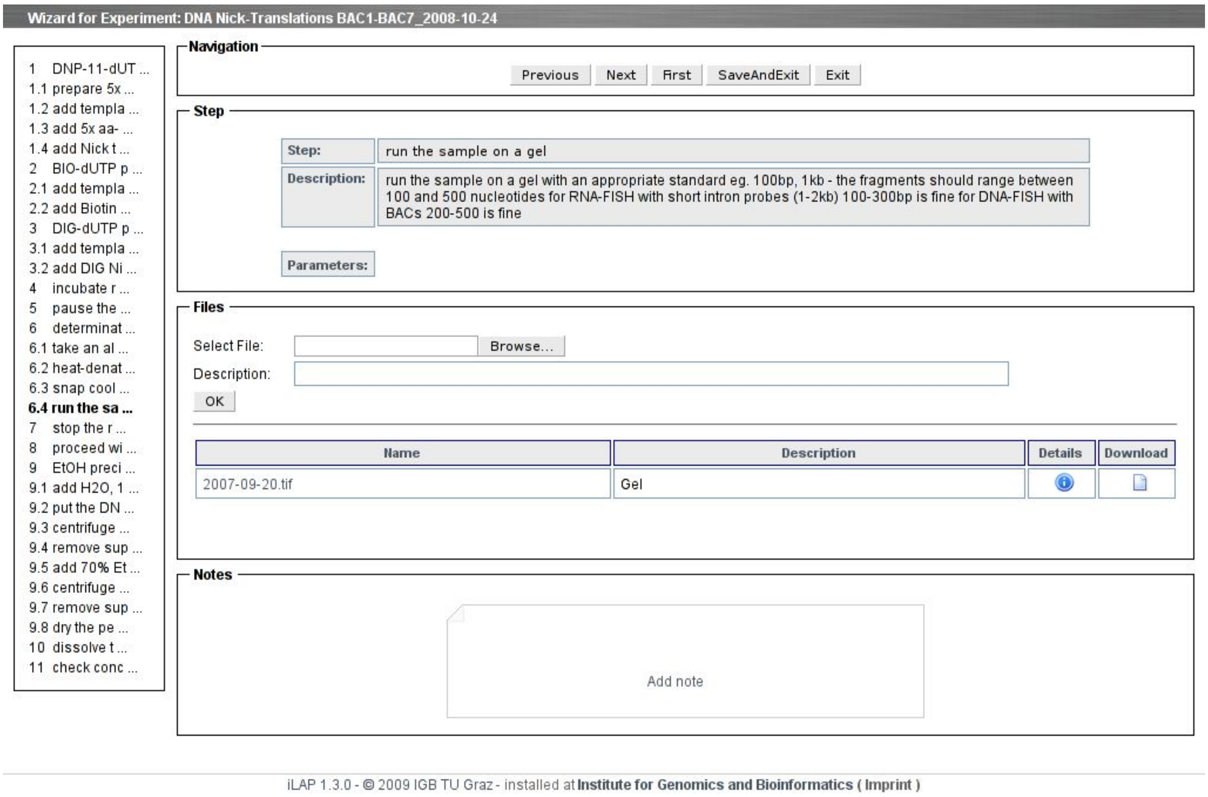

Figure 2.14: experiment wizard

2.3.4.1 Parameter evaluation

To realize the parameter necessities described in section 2.2.4.2 every parameter input is validated when the user is navigating to the next or previous step. The check includes type conformity as well as forcing the input of required parameters. If just one of the given parameter inputs does not pass this check an error message specifying the mistake is shown and all navigation options are disabled until the error is corrected (see figure 2.15 for an example).

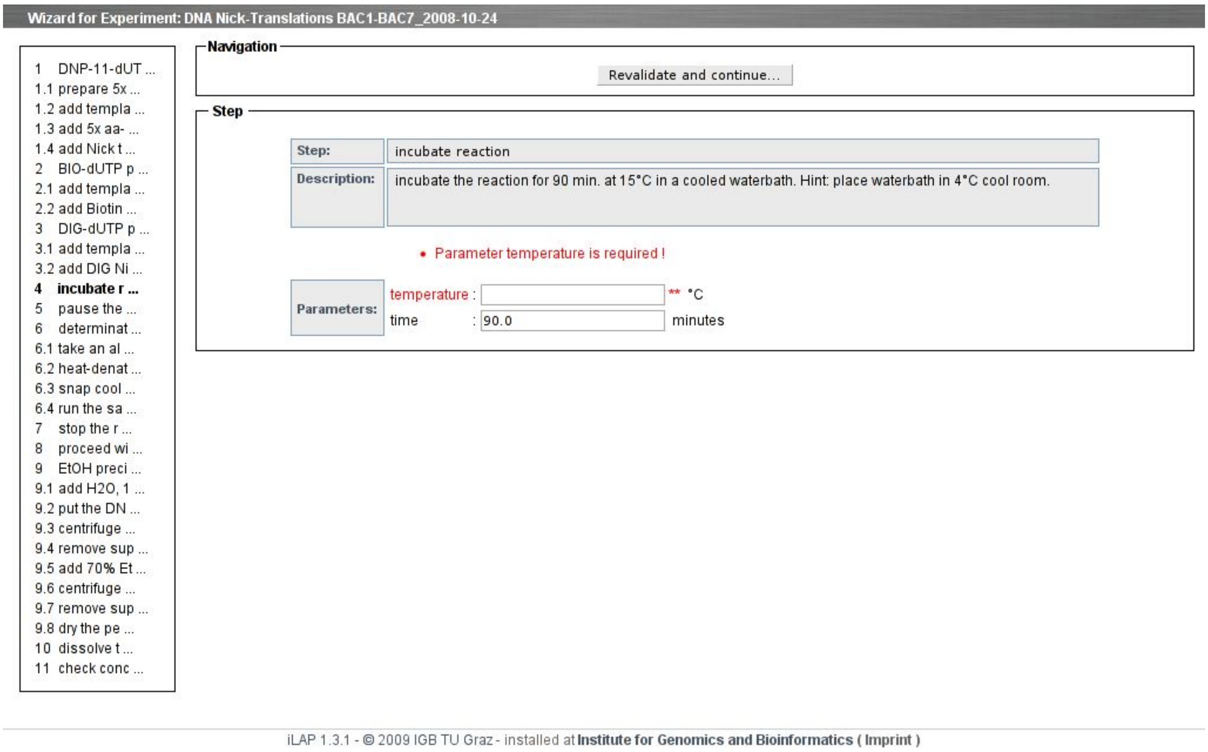

Figure 2.15: experiment wizard - step evaluation failed

2.4 Data analysis

Further analyses and post-processing based on previously generated (raw) result files can be performed and documented within iLAP. This facilitates keeping track of the development of results. In order to integrate results gained by third party softwares iLAP introduced two different types of analyses:

- **internal** describes analyses that are performed within the system and therefore are directly accessible via iLAP.
- **external** incorporates analyses performed by external analysis softwares into the system.

In general analyses are organized in so-called analysis steps which hold information about a single analysis including name, description, required input/output files and their corresponding file types, as well as analysis specific parameters. Since analyses base on result files the link to create a new analysis step is only available once files were attached to experiments or previously performed analyses. To allow files, that resulted themselves from other analyses, to act again as input enables the mapping of whole analyzing cascades. The variety of valid

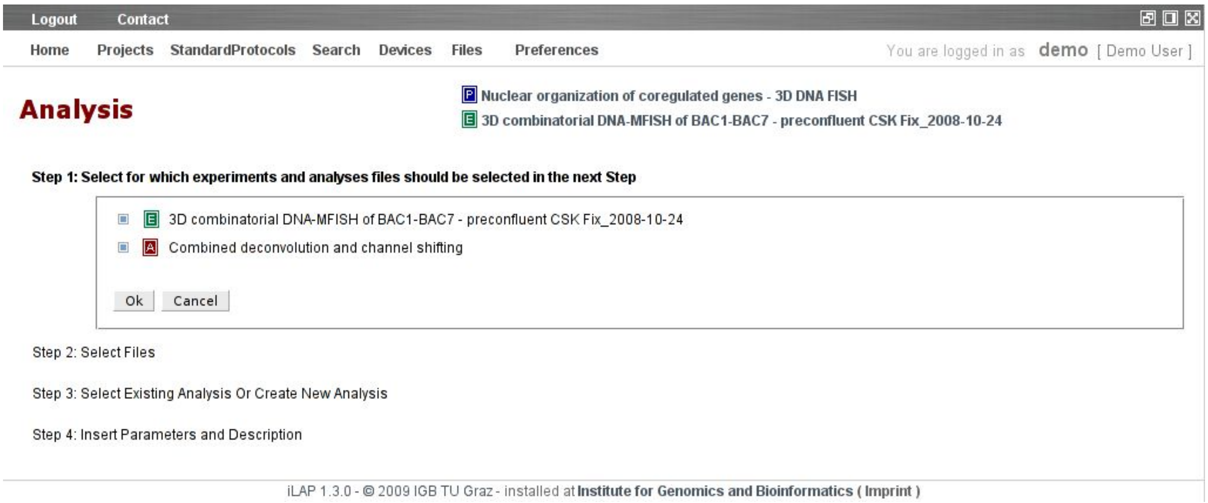

Figure 2.16: analysis step specification - phase 1

inputs will lead into unmanageable overviews if a simple listing of all potential input files is done. Therefore the specification of an analysis step is divide into four stages: contributor selection, input file selection, analysis template selection or creation, and parameter specification. Figure 2.16 shows the start screen for defining a new analysis step. Here the user specifies which experiments or analyses are selected for further investigation. Multiple selection boxes ensure to include input files from different entities. After clicking OK a list showing all chosen contributors's attached files enables the user to select input files for post-processing. Having all input

files defined, the analysis which is actually performed on the data needs to be specified. In this phase the user can either pick one of the already used analysis templates, that are available on institute's and owner basis, or create a new analysis template. The template should include all information to explicitly reconstruct an analysis. If the user decides to reuse already defined analysis templates only the templates matching the types of the given input files are displayed to be selected from. At the last part analysis specific parameters and an additional description providing further information about this particular analysis step are defined. Similar to CWP step parameters iLAP distinguishes between optional and mandatory analysis parameters too. Only when all required parameters are filled in an analysis step can be successfully committed.

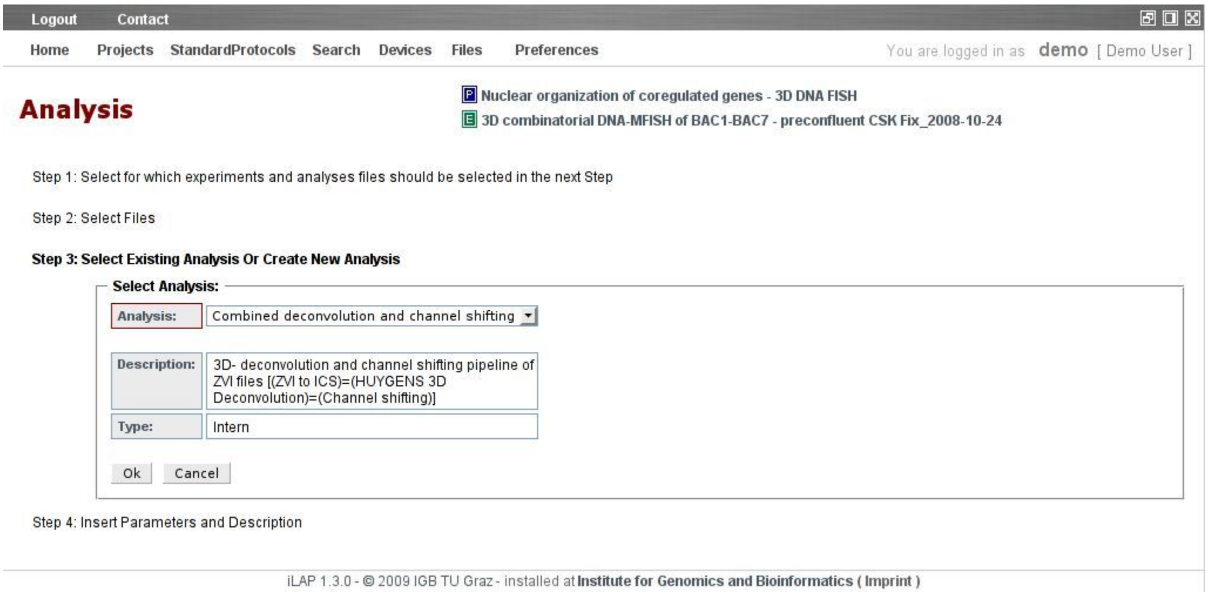

Figure 2.17: analysis step specification - phase 3

2.4.1 External analysis steps

External analysis steps map analyses performed by third party softwares assigning their results to the original raw data files. Parameters used by the analysis software can be documented by forcing the user to specify them within iLAP by creating mandatory analysis step parameters. The data generated at the analysis stage can be easily connected to the raw data by using the already described upload client, which allows connection of the data files with the originating experimental context.

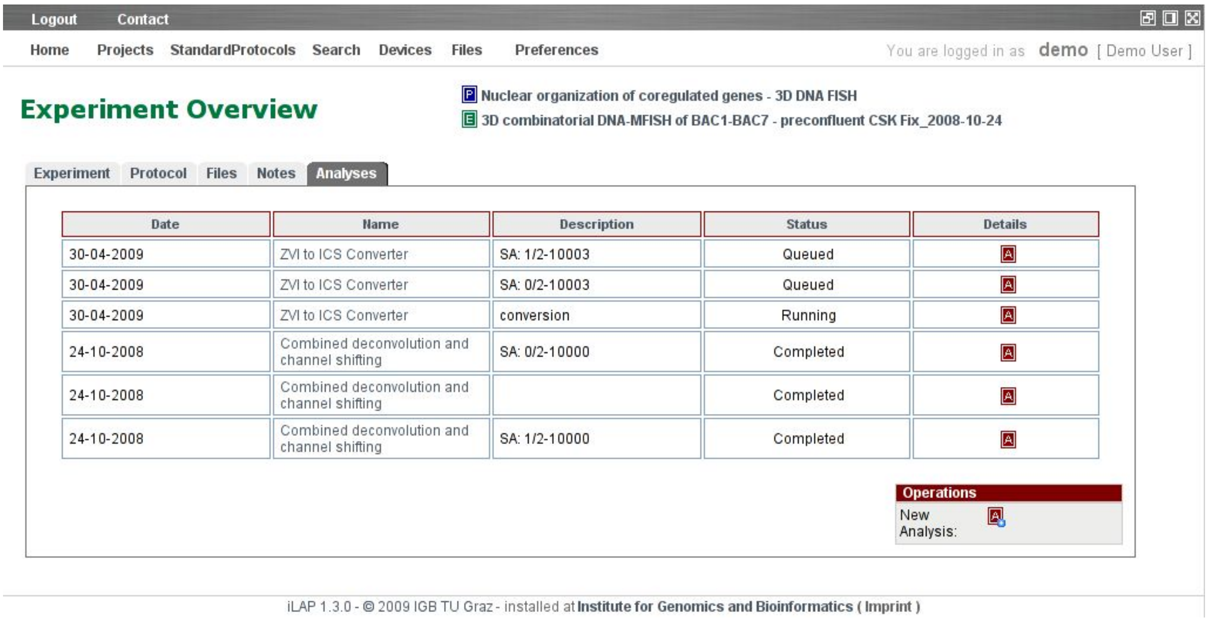

Figure 2.18: experiment analyses list

2.4.2 Internal analysis steps

Internal analysis steps are directly accessible from the server via the Web interface. Once an analysis step is submitted successfully iLAP starts and surveys its execution without any additional user interaction. The current status of the analysis step can be checked at the associated experiment's analysis tab. To enable the integration of additional analyses modules into the system iLAP offers a plug-in system and an application programming interface. As an example, a server analysis deconvolution tool is realized at the demo site (iLAP [2009]). This tool executes the deconvolution of three dimensional stacks on a remote high-performance computing cluster using the JClusterService.

2.4.3 Details

Further information about analysis steps is given on several pages. The experiment's analyses tab (see figure 2.18) shows general information about all analyses performed on the corresponding experiment's data. On this page is listed which and when analyses were performed including the state of each analysis. iLAP distinguishes between queued, starting, running, finalizing, completed, and error states. When clicking on an analysis's name or on its corresponding details link the user is redirected to the experiment's analysis step information page. There the tabs analysis status, analysis definition, input files, output files, and notes contain all data available for a particular analysis step. If the analysis terminates caused by an exception its state is set to error and the error message is displayed. The analysis definition tab provides

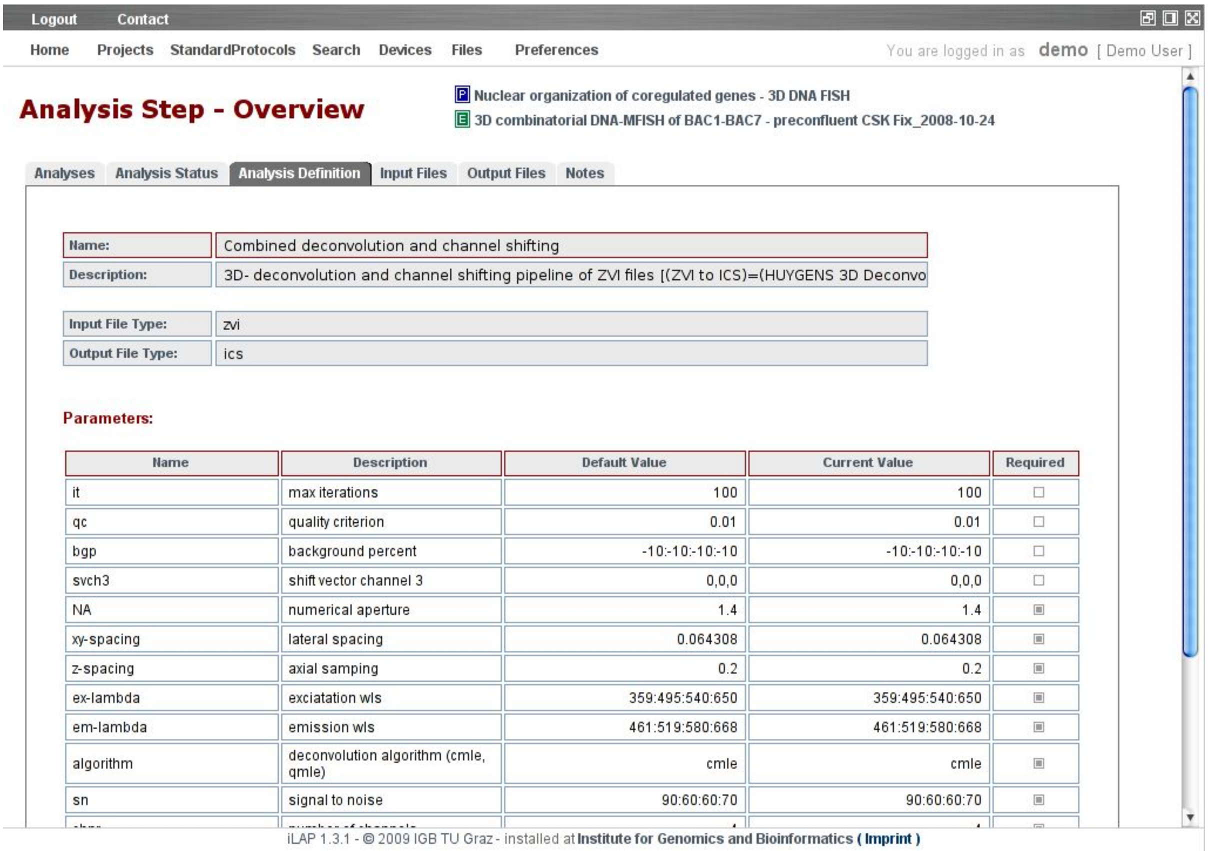

Figure 2.19: analysis step - definition information

general information about the chosen analysis template and lists all specified parameter values. To protect completed analysis step from manipulations all information about status, definition, and input files is read only. A single file upload dialog for output files is only provided for external analysis steps since internal result files are automatically attached by iLAP. Notes can be attached to document comments on analyses regardless of the analysis type.

2.5 Data retrieval

To facilitate finding already inserted data iLAP is providing a search functionality on project, experiment, and note basis. Each search is accessed by selecting the appropriate link on the quick navigation menu. Every query term must consist of at least three characters to avoid exhausting searches. Wildcards are not used as substitutions for any other characters but are interpreted literally.

2.5.1 Project search

The project search allows the user to search for terms used in a project's name, description or aim. It is possible to in- and exclude each field from a search. Projects matching the query are displayed without restriction concerning their hierarchical display level in a list of projects. An arrow on the left side enables a more detailed view of the selected result. By clicking on the P-icon on the right, the detail view of the corresponding project is opened. Figure 2.20 shows a typical search result and its matching projects.

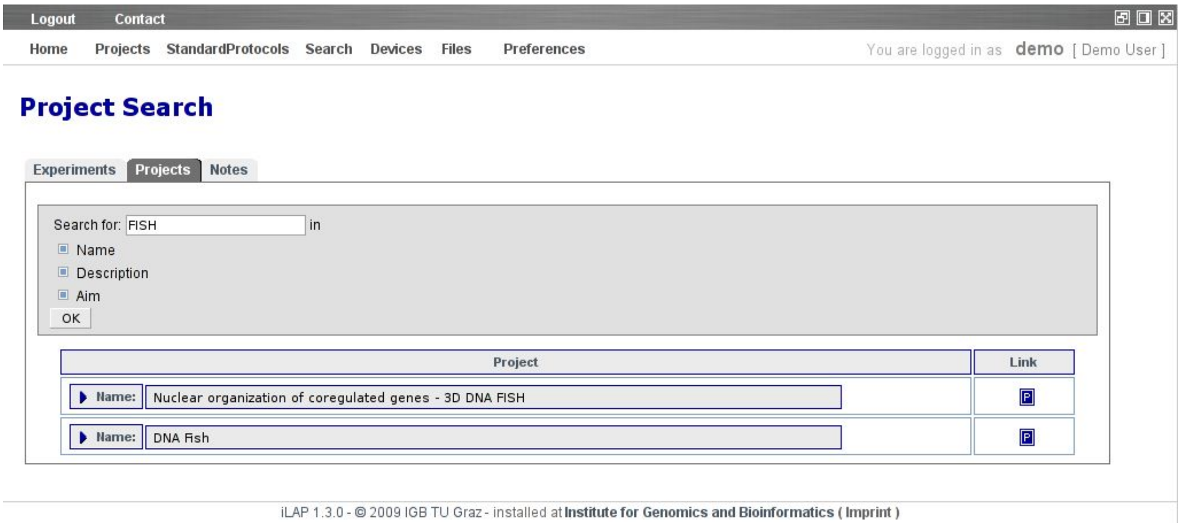

Figure 2.20: search form displaying two matching projects

2.5.2 Experiment search

The experiment search queries an experiment's name, question or description. All search options and result views follow the same logic as the project search (consult section 2.5.1 for further details).

2.5.3 Note search

The note search functionality covers the retrieval of notes attached to projects, analyses, experiments and CWP steps. Specified terms can be found within the note title as well as the note content. The search results are displayed as a list, allowing the user to select a specific result by clicking on its icon. This, however, does not open the note found but the object to which the note is attached (e.g. an experiment or project). A note search results displaying several results in different categories is shown in figure 2.21.

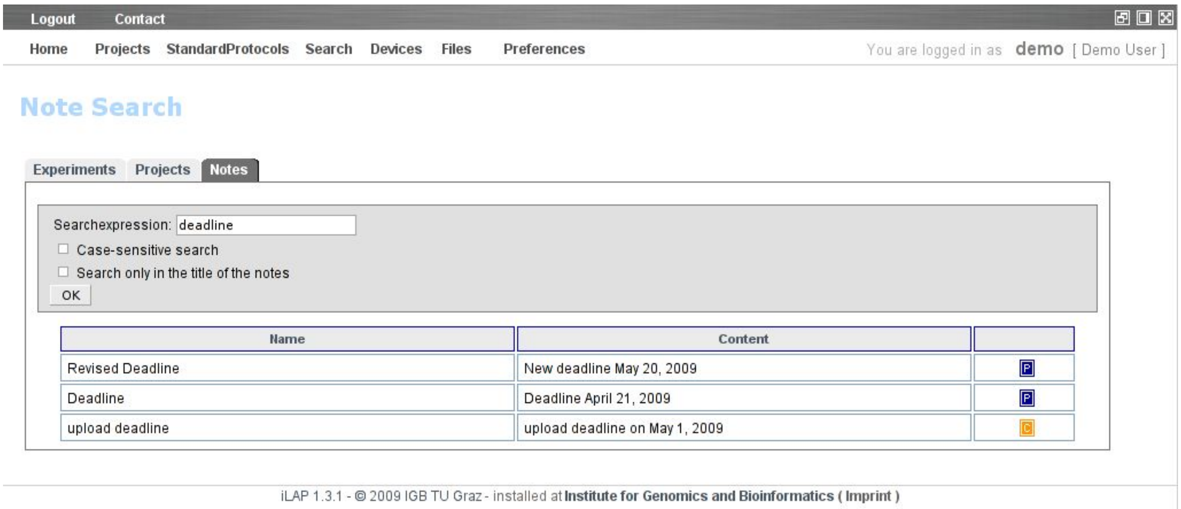

Figure 2.21: note search results displaying several results in different categories

Chapter 3

Software development

3.1 Analysis module development

In order to develop additional anaylsis modules Java developers can simply create a Java class which implements the Java interface AnalysisPlugin.java and place the compiled class together with all dependency classes in the plugin directory of iLAP. iLAP is loading the class dynamically from the plugin directory during the internal analysis execution.

```
/**
 * Description:
 *
 * @author Gernot Stocker, gernot.stocker@tugraz.at
 *
 * Project    : [iLAP]  at.tugraz.genome.smile.analysis.plugin.deconvolution.AnalysisPlugin.java
 * Created on : Feb 28, 2007
 */
package at.tugraz.genome.smile.analysis.plugin;

import java.util.List;

import at.tugraz.genome.smile.service.configuration.ApplicationConfigurationVO;
import at.tugraz.genome.smile.valueobject.analysis.AnalysisDefDetailedVO;
import at.tugraz.genome.smile.valueobject.analysis.AnalysisStepDetailedVO;

/**
 *
 * iLAP's generic PLUGIN-System:
 * ============================
 *
 * This interface defines the methods which are used for lifecycle management of a dynamically loaded/created
 * plugin. The at.tugraz.genome.smile.analysis.plugin.AnalysisPluginFactory is used for instanciating the
 * Plugin in the JMS-remoted MessageDrivenAnalysisManipulatorImpl. The JMS queue starts 20 MessageDrivenAnalysis-
 * Manipulator objects contemporarely for parallel processing of Analysis requests. The Starter performs aferwards
 * the following lifecycle method calls:
 *
 * 1.) boolean canAnalyze(AnalysisStepDetailedVO analysis);  ...
 *     checks, if the analysis can be performed by the instantiated plugin or exits with an error
 *
 * 2.) boolean isBlocking(AnalysisStepDetailedVO analysis) throws PluginExecutionException;
 *     A.) false: non-blocking plugins are started immediately and can return SubAnalysis which are submitted
 *         again via the AnalysisManagement. After having started the AnalysisStep with all it's SubAnalysisSteps
 *         the control is passed over to the AnalysisSupervisor.
 *
 *     B.) true: blocking plugins are started within the same thread
 *         and the Starter waits until the execution is finished.
 *         After that the Starter executes:
 *         1.) boolean isAnalysisDone(AnalysisStepDetailedVO analysis) throws PluginExecutionException;
 *              a.) false: exit with error and setting the error message in the statemessage
 *              b.) true: continue
 *         2.) public File[] getAnalysisResults(AnalysisStepDetailedVO analysis) throws PluginExecutionException;
 *             persists all result files and associates the result file with the current analysis
 *         3.) public void finalizeAnalysis(AnalysisStepDetailedVO analysis) throws PluginExecutionException;
 *
 * 3.) The AnalysisSupervisor checks repeatedly the running non-blocking analysis applications by instantiating
 *     again the plugin using the AnalysisPluginFactory. Than boolean isAnalysisDone-method is called. If it returns
 *     false the AnalysisSupervisor continues with the next running analysis. If it returns true the Supervisor
 *     continues with 2.B.2.) and 2.B.3.)
 *
 * For default implementations of some methods use AbstractAnalysisPlugin.
 *
 * AnalysisSupervisor:
 * ==================
 * The AnalysisSupervisor is responsable for finalizing non-blocking analysis steps. It checks an analysis step for
 * being finished. This is done by using the Plugin internal public boolean isAnalysisDone method. If the analysis
 * depends on subanalysis steps the supervisor checks for the status of the subanalysis first. If one of the
 * subanalyses is finished it calls the finalizeAnalysis of the subanalysis. If all of the subanalyses are either
 * completed or in error state the supervisor calls the finalizeAnalysis method of the parent.
```

```
 *
 */
public interface AnalysisPlugin
{

        /**
         * returns the analysis definition provided by the plugin
         *
         * @param analysis
         * @return
         * @throws PluginExecutionException
         */
         public AnalysisDefDetailedVO getPluginDefinition();

        /**
         * Starts the analysis process internally
         *
         * @param analysis
         * @return
         * @throws PluginExecutionException
         */
        public List<AnalysisStepDetailedVO> startAnalysis(AnalysisStepDetailedVO analysis) throws PluginExecutionException;

        /**
         * Updates the Analysis Status of the analysis according the status of the analysis
         *
         * @param analysis
         * @return
         * @throws PluginExecutionException
         */
        public boolean isAnalysisDone(AnalysisStepDetailedVO analysis) throws PluginExecutionException;

        /**
         * Updates the Analysis Status of the analysis according the status of the analysis
         *
         * @param analysis
         * @return
         * @throws PluginExecutionException
         */
        public void updateAnalysisStatus(AnalysisStepDetailedVO analysis) throws PluginExecutionException;

        /**
         * Returns all result files of an analysis
         *
         * @param analysis
         * @return
         * @throws PluginExecutionException
         */
        public List<ResultDataObjectDetailedVO> getAnalysisResults(AnalysisStepDetailedVO analysis)
                throws PluginExecutionException;

        /**
         * Cleans up all analysis internal intermediate data/files/leftovers ;-)
         *
         * @param analysis
         * @throws PluginExecutionException
         */
        public void finalizeAnalysis(AnalysisStepDetailedVO analysis) throws PluginExecutionException;

        /**
         * Returns wether the plugin blocks execution of the current thread till end of plugin execution
         * or not. Depending on this the plugin life cycle is taking a different path.
         *
         * @param analysis
         * @return
         * @throws PluginExecutionException
         */
        public boolean isBlocking(AnalysisStepDetailedVO analysis) throws PluginExecutionException;

        /**
```

```
         * Returns true only if the analysis step can be handled by this plugin
         *
         * @param analysis
         * @return
         */
        public boolean canAnalyze(AnalysisStepDetailedVO analysis) throws PluginExecutionException;


        /**
         * This is used for internal application specific configuration passed via ApplicationConfigurationVO
         */
        public void setApplicationConfiguration(ApplicationConfigurationVO configuration);

}
```

Additionally the module must be defined in the database like an external analysis module extended by the class name. The so defined analysis step should appear immediately in the analysis selection box of internal applications.

3.2 SOAP interface for external access

By using the SOAP based transfer classes following the ConnectionManagerInterface.java Interface one can eassily transfer data between the local machine to the iLAP server back and forth.

```
/**
 * Description:
 *
 * @author Gernot Stocker, gernot.stocker@tugraz.at
 *
 * Project     : [MultipleFileUploader]  at.tugraz.genome.mfu.connection.ConnectionManagerInterface.java
 * Created on : Jun 4, 2005
 */
package at.tugraz.genome.mfu.connection;


import java.io.File;

import at.tugraz.genome.mfu.config.ServerDefinition;
import at.tugraz.genome.mfu.exception.ConnectionManagerException;
import at.tugraz.genome.mfu.transfer.CustomTreeNode;
import at.tugraz.genome.mfu.transfer.soap.NodeDTO;

public interface ConnectionManagerInterface {

        // Global UserData
        public void setGlobalUserData(String username, String password);
        public boolean isGlobalUserDataInitialized();

        // Managing ServerConnections
        public void deleteServerConnectionDefinition(ServerDefinition server) throws ConnectionManagerException;
        public void changeServerConnectionDefinition(ServerDefinition server) throws ConnectionManagerException;
        public void addNewServerConnectionDefinition(ServerDefinition server) throws ConnectionManagerException;

        // Managing node on the Server
        public boolean deleteNodeAndAllSubNodes(CustomTreeNode node_todelete) throws ConnectionManagerException;
        public NodeDTO createNewFileContainerNode(CustomTreeNode parent, CustomTreeNode new_node)
                throws ConnectionManagerException;
        public NodeDTO uploadFile(CustomTreeNode target, String displayname, String description, File uploadfile)
                throws ConnectionManagerException;
        public boolean downloadFile(CustomTreeNode  source, File destinationfile)
                throws ConnectionManagerException;
        public NodeDTO[] getNodeChildren(CustomTreeNode parent) throws ConnectionManagerException;
```

}

A good example for such a transferclient is already implemented as a ImageJ plugin which can be seen in the folling screenshot:

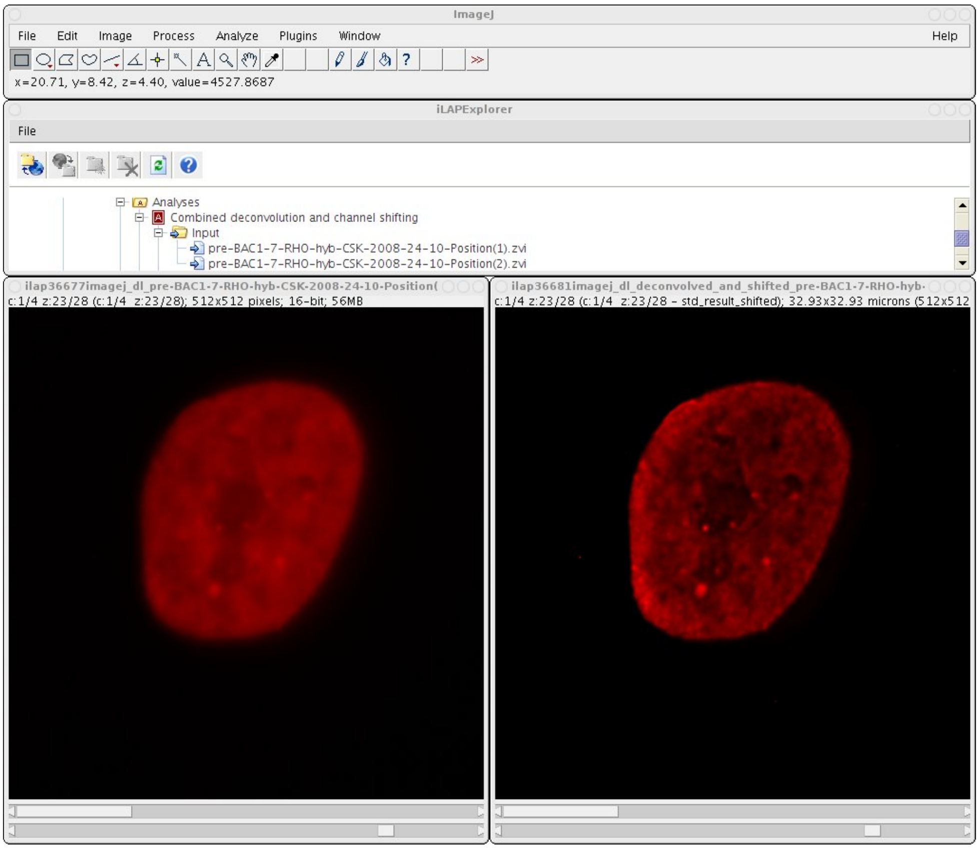

Appendix A

Bibliography

Article references

[Abramoff, 2004] M.D. Abramoff. Image Processing with ImageJ. *Biophotonics International*, 11:36–42, 2004.

[Deutsch et al., 2008] Eric W Deutsch, Catherine A Ball, Jules J Berman, G. Steven Bova, Alvis Brazma, Roger E Bumgarner, David Campbell, Helen C Causton, Jeffrey H Christiansen, Fabrice Daian, Delphine Dauga, Duncan R Davidson, Gregory Gimenez, Young Ah Goo, Sean Grimmond, Thorsten Henrich, Bernhard G Herrmann, Michael H Johnson, Martin Korb, Jason C Mills, Asa J Oudes, Helen E Parkinson, Laura E Pascal, Nicolas Pollet, John Quackenbush, Mirana Ramialison, Martin Ringwald, David Salgado, Susanna-Assunta Sansone, Gavin Sherlock, Christian J Stoeckert, Jason Swedlow, Ronald C Taylor, Laura Walashek, Anthony Warford, David G Wilkinson, Yi Zhou, Leonard I Zon, Alvin Y Liu, and Lawrence D True. Minimum information specification for in situ hybridization and immunohistochemistry experiments (MISFISHIE). *Nat Biotechnol*, 26(3):305–312, Mar 2008. doi: 10.1038/nbt1391. URL http://dx.doi.org/10.1038/nbt1391.

[McNally et al., 1999] J. G. McNally, T. Karpova, J. Cooper, and J. A. Conchello. Three-dimensional imaging by deconvolution microscopy. *Methods*, 19:373–385, 1999.

Web link references

[iLAP, 2008] iLAP. Last visited on 01/05/2009, 2008. URL http://genome.tugraz.at/iLAP.

[iLAP, 2009] iLAPdemo. Last visited on 01/05/2009, 2009. URL http://ilapdemo.genome.tugraz.at/iLAP.

[OMERO, 2009] OMERO.editor. Last visited on 01/05/2009, 2009. URL http://trac.openmicroscopy.org.uk/shoola/wiki/OmeroeditorBetafour.

List of Figures

2.1 iLAP header and quick navigation . . . 7
2.2 overview page showing a selected project's information . . . 8
2.3 general project overview . . . 11
2.4 experiment: protocol tab showing an empty CWP . . . 12
2.5 experiment: protocol tab showing an already defined CWP . . . 13
2.6 general overview of a simple CWP step . . . 14
2.7 overview showing a parameter's details . . . 15
2.8 generated PDF . . . 16
2.9 SOP overview . . . 17
2.10 page handling a project's authorized users and groups. . . . 18
2.11 project note tab . . . 19
2.12 project single file upload . . . 20
2.13 Java applet for multiple file transfer . . . 21
2.14 experiment wizard . . . 22
2.15 experiment wizard - step evaluation failed . . . 23
2.16 analysis step specification - phase 1 . . . 24
2.17 analysis step specification - phase 3 . . . 25
2.18 experiment analyses list . . . 26
2.19 analysis step - definition information . . . 27
2.20 search form displaying two matching projects . . . 28
2.21 note search results displaying several results in different categories . . . 29

Appendix B

Glossary

AAS	Authentication and Authorization System
ACEGI	Acegi Security framework (1st,3rd,5th,7th,9th character in the alphabet)
ACIS	Atomicity, Consistency, Isolation and Durability
ACL	Access Control Lists
AOP	Aspect Oriented Programming
API	Application Programming Interface
Axis	Apache eXtensible Interaction System
CAS	Common Authentication Service
CASE	Computer-Aided Software Engineering
CIMP	Computational Independent Model
CRUD	Creating, Reading, Deleting and Updating
CWP	Current Working Protocol
DAO	Data Access Object
DC	Dependency Injection
DCL	Data Control Language
DDL	Data Definition Language
DML	Data Manipulation Language
EIS	Enterprise Information System
EJB	Enterprise Java Beans
ERP	Enterprise Resource Planning systems
FISH	Fluorescence In-Situ Hybridization
FLIP	Fluorescence Loss in Photobleaching
FRAP	Fluorescence Recovery After Photobleaching
FRET	Fluorescence Resonance Energy Transfer
GFP	Green Fluorescence Protein

GID	Global Image Database
GUI	Graphical User Interface
HQL	Hibernate Query Language
HTTP	Hypertext Transport Protocol
iLAP	information managament system for Laboratorydata Analysis and Protocoldevlopment
IDE	Integrated Development Environment
IoC	Inversion of Control
IT	Information Technology
J2EE	Java 2 Enterprise Edition
JAAS	Java Authentication and Authorization API
JAXB	Java Architecture for XML Binding
JAXP	Java API for XML processing
JAXR	Java API for XML Registries
JAXRPC	Java API for Remote Procedure Calls
JDBC	Java Database Connectivity
JDO	Java Data Objects
JMS	Java Messaging Service
JMX	Java Management Extension
JNDI	Java Naming and Directory Interface
JSP	Java Server Pages
JTA	Java Transaction API
LAM	Local Area Multicomputer Messa Passing Interface
LDAP	Light Weight Application Protocol
MDA	Model Driven Architecture
MDP	Message Driven POJO
MDR	Metadata Repository
MIME	Multipurpose Internet Mail Extensions
MOF	Meta Object Facility
MOM	Java Message Oriented Middleware
MPI	Message Passing Interface
MVC	Model View Controller
ODBC	Open DataBase Connectivity
OGNL	Object Graph Navigation Languag
OME	Open Microscopy Environment
ORM	Object Relational Mapping
PDF	Portable Document Format

PIM	Platform Independent Model
POJO	Plain Old Java Object
POM	Project Object Model
PSM	Platform Specific Model
PVM	Parallel Virtual Machine
QBC	Query By Criteria
QBE	Query By Example
RDBMS	Relational Database Management System
RMI	Remote Method Invocation
RPC	Remote Procedure Call
SAAJ	SOAP with Attachments API for Java
SFSB	StateFull Session Bean
SLSB	StateLess Session Bean
SOAP	Simple Object Access Protocol
SQL	Structured Query Language
UDDI	Universal Description, Discovery and Integration
UML	Unified Modelling Language
WML	Wireless Markup Language
WSDL	Web Service Description Language
XMI	XML Metadata Interchange

Appendix C

Acknowledgments

This work was supported by GENAU: BIN, Bioinformatics Integration Network.
