# Supplementary material for: iLAP: a workflow-driven software for experimental protocol development, data acquisition and analysis
Source: BMC Bioinformatics. 2009 Nov 26;10:390. doi: 10.1186/1471-2105-10-390 (PMC2789074; doi:10.1186/1471-2105-10-390)
Supplement: Additional file 2 — iLAP installation and administration manual. The iLAP installation and administration manual contains a detailed description of the installation process for all supported platforms including screen shots. [file 1471-2105-10-390-S2.PDF]

LABORATORY DATA MANAGEMENT SYSTEM, ANALYSIS- AND PROTOCOL DEVELOPMENT

INSTALLATION AND ADMINISTRATION MANUAL

(Version 1.3.2)

Authors:

Gernot Stocker, gernot.stocker@tugraz.at
Maria Fischer, maria.fischer@tugraz.at
Simon Kainz, simon.kainz@tugraz.at

Graz University of Technology
Institute for Genomics and Bioinformatics
Petersgasse 14, 8010 Graz, Austria

Contents

1 Installation . . . 1

1.1 Installation overview . . . 1
1.2 Quick installation instructions . . . 2
1.3 Installation preparations . . . 3
1.3.1 Prerequisites and planning of the installation . . . 3
1.3.2 Preparation of the database users and the necessary databases . . . 3
1.3.3 Optional creation of a local system user . . . 5
1.4 Wizard installation . . . 6
1.5 Starting iLAP . . . 13

2 Usermanagement administration . . . 14

2.1 Genome Usermanagement . . . 14
2.1.1 Login into the usermanagement . . . 14
2.1.2 Creation of an institute within the Genome Usermanagement . . . 15
2.1.3 Creation of a user account for iLAP within the Genome Usermanagement 16
2.2 Simple Usermanagement . . . 17

3 Testing iLAP without local installation . . . 18

3.1 iLAP demo site . . . 18
3.2 Virtual image for quick iLAP testing . . . 18

4 FAQs . . . 19

4.1 How to change the application data path of iLAP manually? . . . 19
4.2 How to change the layout of iLAP manually? . . . 19
4.3 How to uninstall iLAP? . . . 19
4.4 Under which license is iLAP available? . . . 20

Chapter 1

Installation

1.1 Installation overview

iLAP is a web-based JEE application which consists of three different components. **iLAP-Core** is the web-application which enables you to use the web based functionality of iLAP and is delivered in a bundled Apache Tomcat web container. This Web application accesses an **RDBMS** (relational database management system e.g. PostgreSQL) which stores all the iLAP specific data in its tables. Only the files imported in iLAP are stored separately in a dedicated Application-Data-directory which can be specified during the installation process. Authentication and authorization within iLAP is handled externally by dedicated **Usermanagement Systems**. At the moment iLAP supports the in-house developed Genome-AAS or the XML-File based Simple-AAS.
The installation itself is performed in 3 main stages which should not take more than 15 minutes by an experienced system administrator with a PostgreSQL instance:

- Installation preparations:
  - Prerequisites and planning of the installation
  - Creation of a database user and of two databases which can be accessed by this user
  - Optional creation of a local system user under which iLAP and its services should be installed and run
- Installation using the graphical installation wizard
- Initialization and user creation:
  - Starting and stopping the services
  - Depending on the used usermanagement back-end iLAP users must be created and different roles in iLAP must be assigned

1.2 Quick installation instructions

This sections provides the shortest way to get iLAP running at your site. If these instructions are not working at your site please continue reading the subsequent chapters to get a deeper understanding of the installation procedure.

```
 1.) Download a PostgreSQL database from http://www.postgresql.org/download/
     and install it locally. If it is already installed use the local one.
 2.) Create PostgreSQL user via psql:
       CREATE ROLE ilap WITH LOGIN NOSUPERUSER INHERIT CREATEDB
       NOCREATEROLE PASSWORD 'yourpassword';
       CREATE DATABASE "ilap" WITH OWNER = "ilap" ENCODING = 'UTF8';
       CREATE DATABASE "genomeaas" WITH OWNER = "ilap" ENCODING = 'UTF8';
 3.) Create a local system user under which iLAP should be installed
       Linux  : useradd -c "iLAP JEE user" -d /home/ilap -s /bin/bash ilap
                passwd ilap
       Windows: use your working accunt unless you want to install it as
                service
 4.) Switch to the ilap user either by logging in as ilap or by su - ilap
 5.) Download JDK (Version 1.6 update 6) http://java.sun.com, install it
     and set the JAVA_HOME variable
       Linux  : sh ./jdk-6u13-linux-i586.bin
                export JAVA_HOME=/home/ilap/jdk1.6.0_013
                export PATH=$JAVA_HOME/bin:$PATH
       Windows:
               jdk-6u13-windows-i586-p.exe
 6.) Download iLAP from http://genome.tugraz.at/iLAP/iLAPdownload.php
 7.) Start Wizard under a graphical interface (Windows or local/ssh-redi-
     rected X):
       Linux console: java -jar iLAP-1.3.2-default-install.jar
       Windows      : double click on iLAP-1.3.2-default-install.exe
 8.) Only change the database settings in the wizard
 9.) Start genomeaas and ilap via shortcuts/shell e.g.
       Linux  : iLAP/AAS/Genome-AAS/bin/run-usermanagement.sh
                iLAP/iLAPCore/bin/startup.sh
10.) Connect to http://localhost:8080/iLAP
11.) Login with the username and password you have specified at the wizard
     screen
```

1.3 Installation preparations

1.3.1 Prerequisites and planning of the installation

Before you start with the iLAP installation you must have installed and started a **PostgreSQL database instance** which has its TCP/IP connectivity enabled. It must be reachable by the host which is running iLAP. For more details about installation and configuration of a PostgreSQL database have a look at the following website http://www.postgresql.org.
Under Linux/Unix systems the configuration file of the database instance postgresql.conf must contain at least the instruction:

```
listen_addresses = 'localhost' # what IP address(es) to listen on;
                               # comma-separated list of addresses;
                               # defaults to 'localhost', '*' = all
port = 5432
```

Additionally to the running database there must be installed a **Sun Java Development Kit of version 1.5 or higher**. Under Linux/Unix systems you can test your JDK installation with the following lines in the command line:

```
#
# determine the version of your Java installation in your environment
#
[ilap@obeline ~]$ java -version
java version "1.6.0_13"
Java(TM) SE Runtime Environment (build 1.6.0_13-b03)
Java HotSpot(TM) Client VM (build 11.3-b02, mixed mode, sharing)
```

Finally you must provide enough disk space for storing the imported data associated within iLAP projects, protocols and analysis steps. The **adequately dimensioned disk system** must be accessible as a local directory path which by default is set to a subdirectory of the iLAP installation path. Have in mind that microscopy data can grow rapidly but don't panic because the ApplicationData path can be changed after the installation easily in a property file and the migration process consists in simply copying the content of the ApplicationData to the new destination.

1.3.2 Preparation of the database users and the necessary databases

There are several ways to create database users in PostgreSQL. The following two sections are describing the steps necessary to perform using different methods.

1.3.2.1 Using system commands

Create a database user e.g. ilap on the PostgreSQL database server and set a password using the following command under Linux/Unix.
For PostgreSQL 7.x

```
createuser -W -D -A ilap
> Password:
> CREATE ROLE
createdb -U ilap -h localhost -p 5432 -O ilap -E UTF8 ilap
> CREATE DATABASE
createdb -U ilap -h localhost -p 5432 -O ilap -E UTF8 genomeaas
> CREATE DATABASE
```

For PostgreSQL 8.x

```
createuser --no-superuser --no-createrole --createdb -W ilap
> Password:
> CREATE ROLE
createdb -U ilap -h localhost -p 5432 --owner ilap --encoding UTF8 ilap
> CREATE DATABASE
createdb -U ilap -h localhost -p 5432 --owner ilap --encoding UTF8 genomeaas
> CREATE DATABASE
```

The user must be able to access the database instance from the machine where iLAP is installed.

1.3.2.2 Using SQL statements as PostgreSQL administrator

Connect as database administrator with your preferred postgresql client to your database server and execute the following SQL statements. Make sure that you have replaced the 'yourpassword' with a save password.

```
--
-- Create the PostgreSQL user for installation using SQL
--
CREATE ROLE ilap WITH LOGIN NOSUPERUSER INHERIT CREATEDB NOCREATEROLE
PASSWORD 'yourpassword';
--
-- Create the databases used during installation
--
CREATE DATABASE "ilap" WITH OWNER = "ilap" ENCODING = 'UTF8';
CREATE DATABASE "genomeaas" WITH OWNER = "ilap" ENCODING = 'UTF8';
```

1.3.3 Optional creation of a local system user

If you want to run iLAP under a distinct local user and not under your own user create a new user in your system. This can be done under Linux/Unix system by the following command:

```
useradd -c "iLAP JEE user" iLAP
```

For more information see “man useradd”. If you have decided to go with a distinct iLAP user run all subsequent commands as the newly created user.

1.4 Wizard installation

Download the newest version of iLAP from http://genome.tugraz.at/iLAP/iLAPdownload.php and store it in a local directory. After that start the graphical installer of iLAP and fill out the required parameters:

`For Windows-based systems use double click on:`

`iLAP-1.3.2-default-install.exe`

`On Linux system run:`

```
java -jar iLAP-1.3.2-default-install.jar
```

`On MacOSX open the installation image and use double click on the installer app:`

`iLAP-1.3.2-default-install.dmg`

Attention: the installer has a graphical user interface and should therefore also be started in a graphical environment which is either Windows or the X environment under Linux/Unix systems. In order to avoid installing X on a server you can also do X- forwarding using SSH to get the user interface redirected to your local machine.
The graphical interface of the installer is starting:

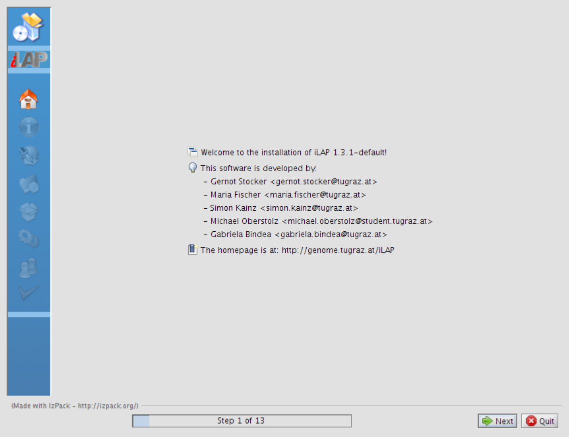

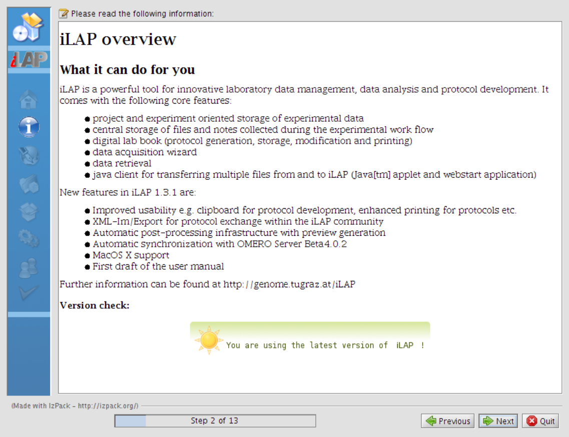

The first two screens show you general informations about iLAP, its version and the major changes between the versions.
After the license agreement you have to specify the directory path, where iLAP should be installed. It is recommended to install it in a single central place preferably under the user home directory of the newly generated system user(see section “Optional creation of a local system user”). All data including the iLAP-handled user files is stored there. It is highly recommended to include this directory into your general backup environment in order to avoid the loss of your precious data.

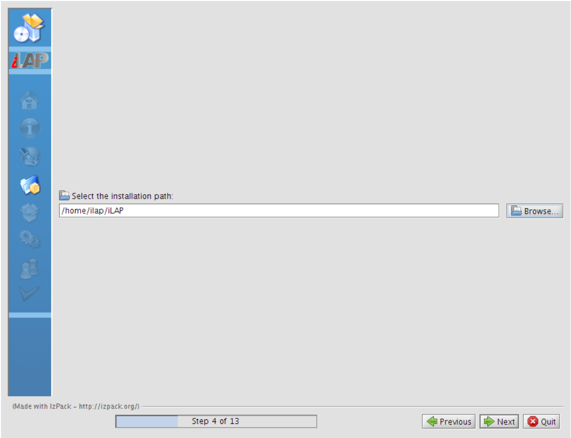

The next wizard screen shows you the package selection dialog. Those packages which are absolutely necessary for an installation can not be deselected. One very important decision which must be taken at this screen is the decision about the usermanagement you intend to use.

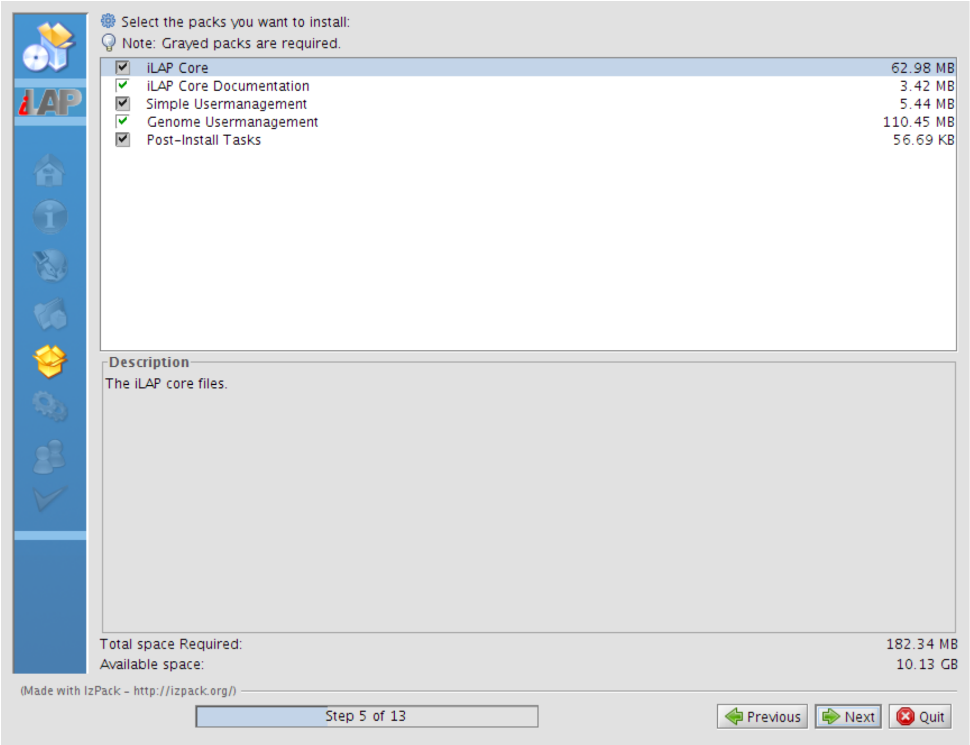

At the moment iLAP supports two different ways for handling users:

- Simple Usermanagement: this usermanagement provides a simple xml-file based usermanagement which should be used only if you have limited amount of users and fluctuation of people is very low. It supports all features which are necessary for running iLAP but it comes only with a command line based administration tool and does not support advanced features like Web Single-Sign-On.
- Genome Usermanagement: The GenomeAAS is a full featured usermanagement and is the recommended usermanagement. It is a J2EE application by it's own and gives

you a versatile web interface for all tasks regarding the user administration. Besides the web-interface it provides Single-Sign-On functionality for web-applications as well as Samba/Linux system user authentication, LDAP-support etc. These advanced features are not necessary for a basic iLAP installation and go far beyond the scope of this document. iLAP relevant features are described in the section "Account creation".

The wizard screen 6 gives you the possibility to specify the TCP/IP port settings under which iLAP should be reachable after the installation and the database connection parameters where iLAP should store your data. If you intend to run only iLAP on your server simply leave the default port settings. If you have already a JEE environment with web-container running on your server change the ports so that you have no conflicts but set them above the reserved ports in the range from 1024 to 65000.

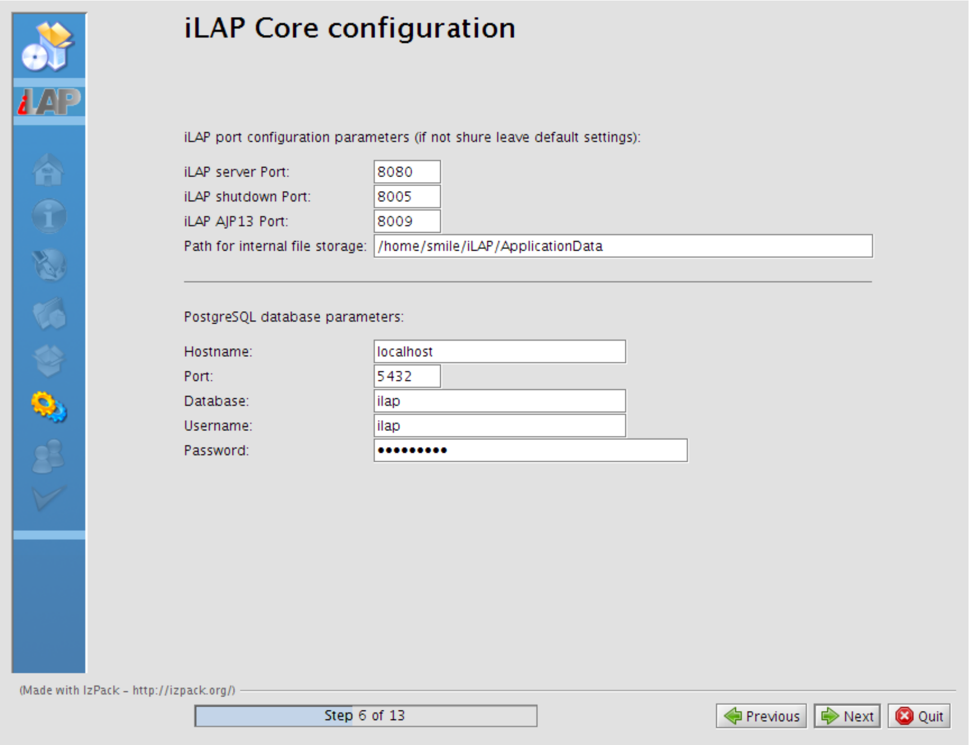

As a practical rule of thumb leave the ports of one single JEE container in the end the same and change only the leading thousand digits e.g. from 8080, 8005, 8009 to 10080, 10005, 10009.

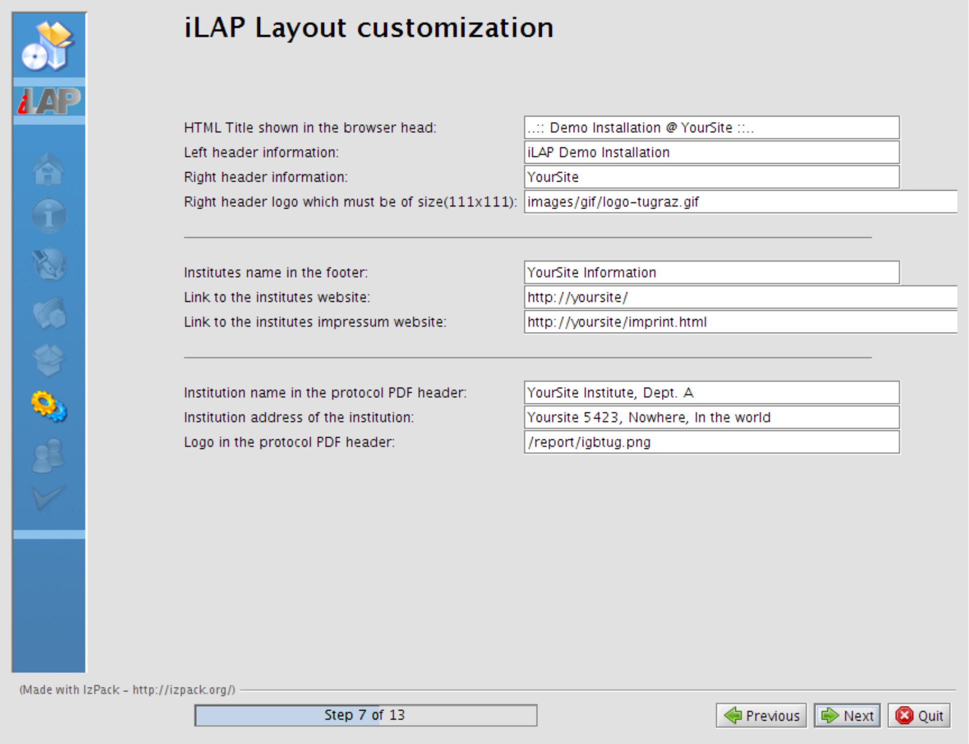

In order to make local customization for the look and feel of your installation you can change the input fields in the wizard screen 7.

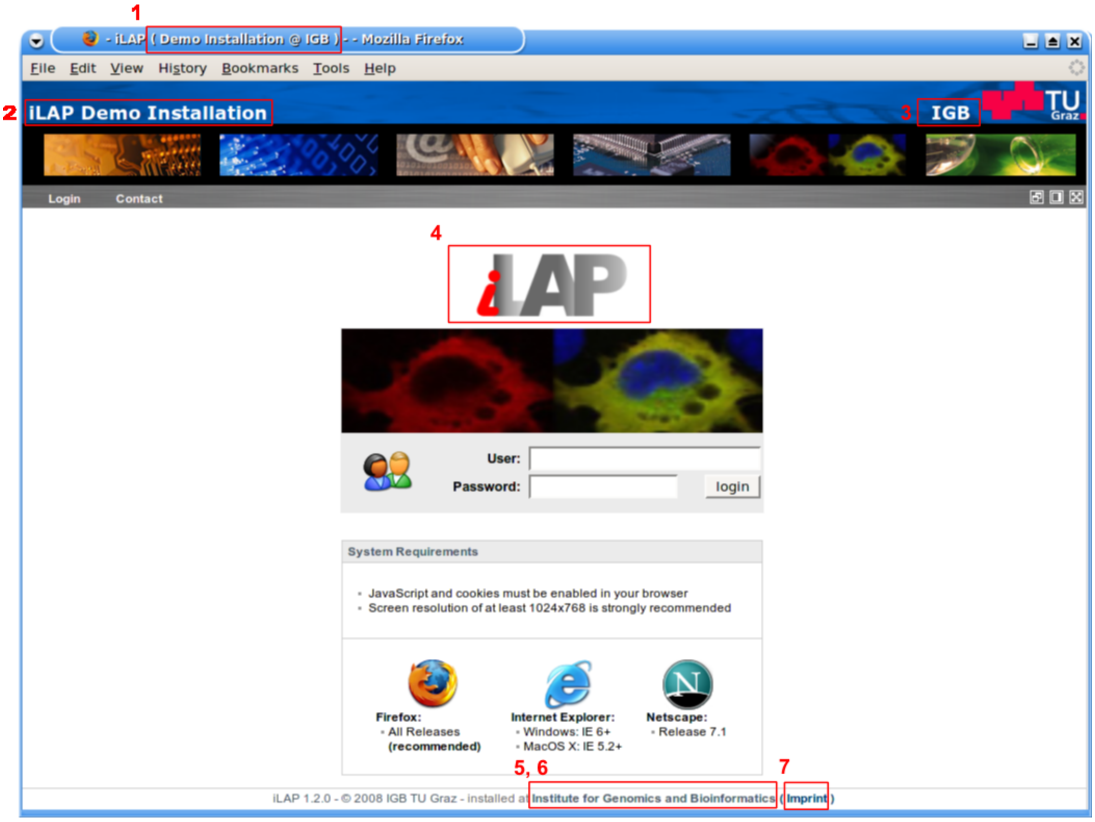

The customization can be best seen in the login page above where the affected areas are highlighted in red:

1. HTML Title shown in the browser head
2. Left header information
3. Right header information and the logo
4. Logo in the center
5. Institutes name in the footer
6. Link behind the institutes name
7. Link behind the Impressum

The third part of the wizard screen 7 affects the pdf generation of the experiment protocol. There you change the header of each page permanently.
At the next screen you can specify the user specific data of the first ilap user. The second half of this screen appears only if the Genome Usermanagement is selected during the package selection.
For the Genome Usermanagement you simply have to specify the name of the database to which the iLAP database user is allowed to connect and the port range under which the Genome Usermanagement is started. This is for simplicity reasons because the JBoss application server behind the Genome Usermanagement uses several TCP/IP ports. Finally you specify the administrator password of the "admin" user with which you can create more users.

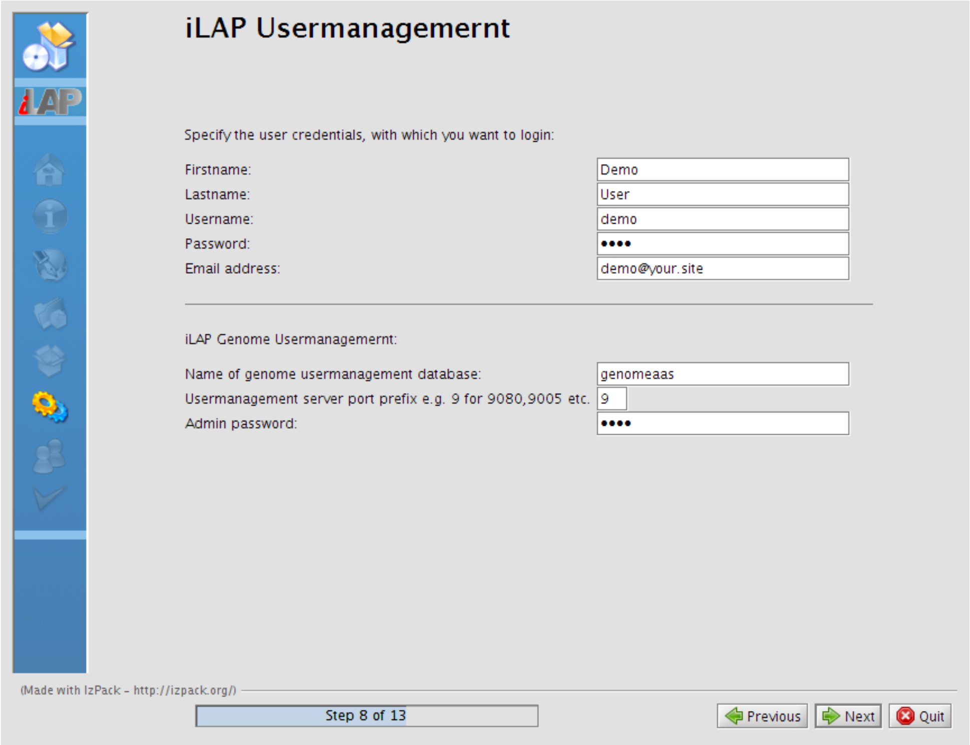

After this customizations you get a summary of the selected installation and with Next you initiate the installation process.

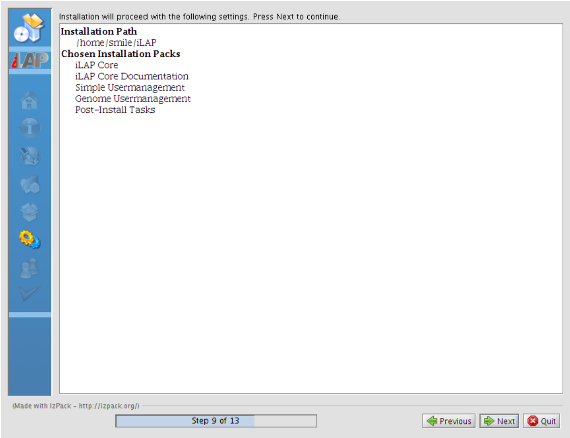

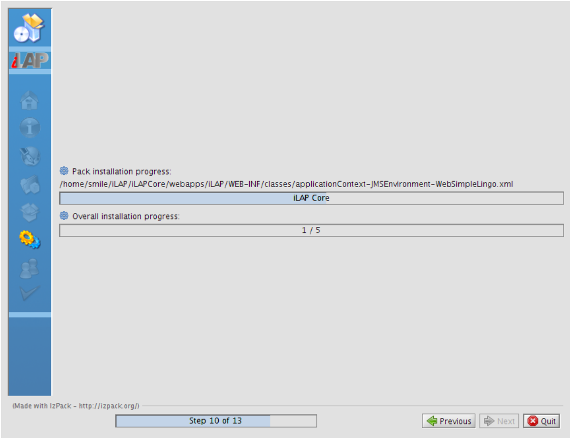

The following “Processing” screen initializes the configuration according to the wizard input and performs the database schema initialization. If you want to perform additional changes after the installation you have to perform the changes manually using an editor and changing the appropriate properties files(see in the FAQ section).

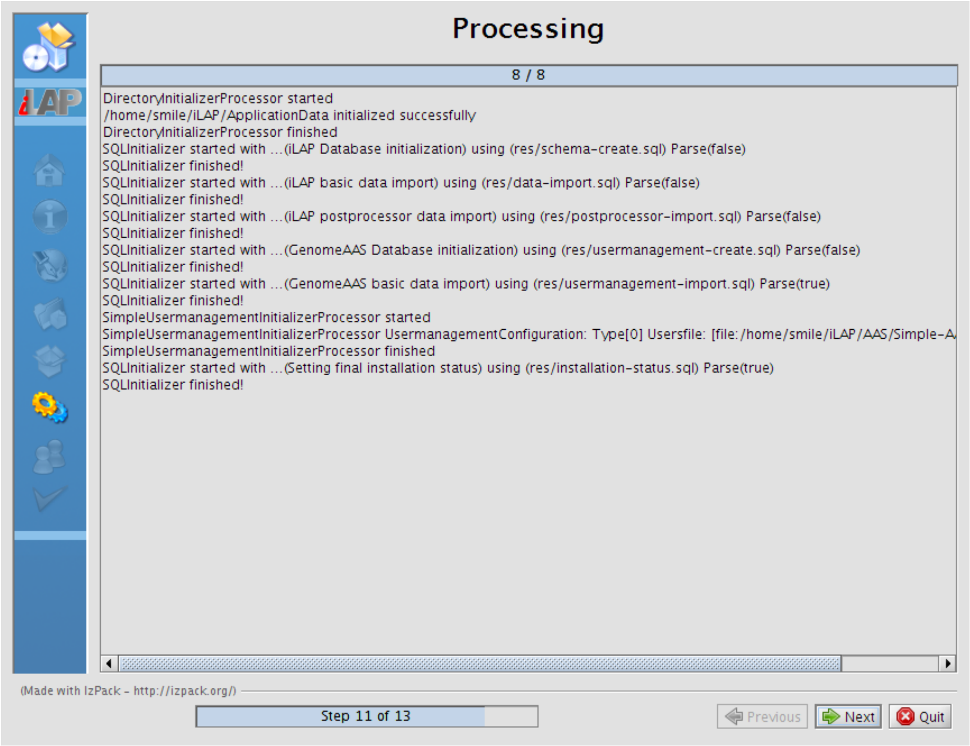

If the installation and initialization process is completed local shortcuts for starting and stopping iLAP and the Genome Usermanagement can be generated using the next screen. This detects the platform specific settings of Linux and Windows automatically.

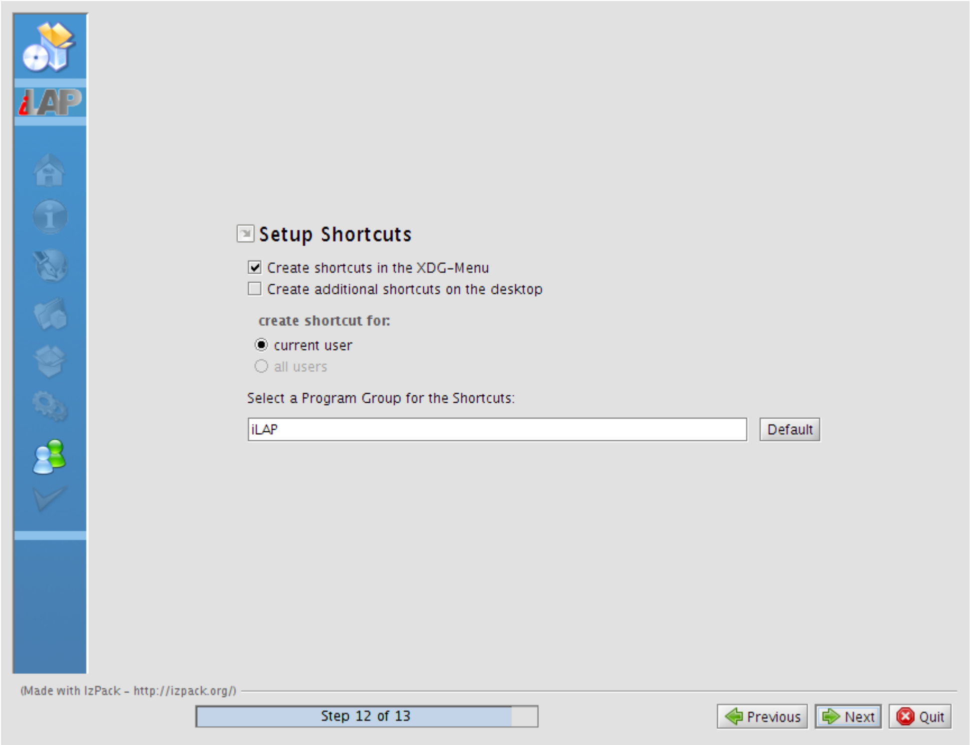

At the final wizard screen you can generate a configuration file for automatic- or text-only installation without demanding user interaction(see section “FAQ: How to perform a text-console-only installation”)

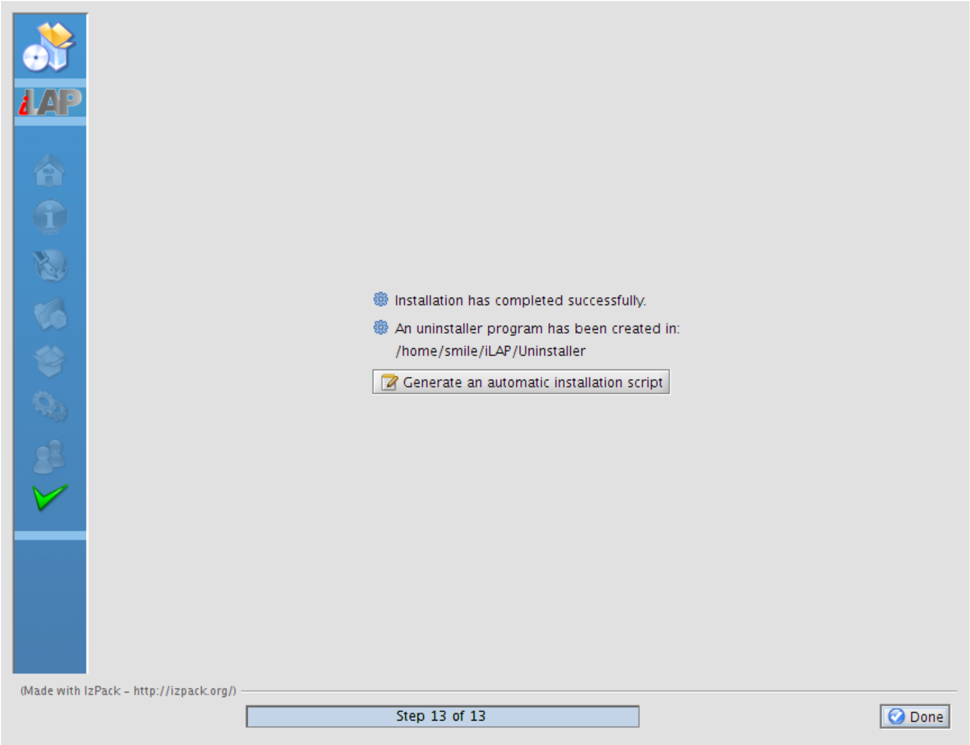

Attention: In general the default values of the installation wizard should be fine and should lead to a working installation of iLAP. If you don’t understand the name of the configuration

settings simply leave them as they are.

1.5 Starting iLAP

If you have chosen to install the shortcuts you can start the necessary services for iLAP by clicking at the Shortcuts “GenomeAAS start” and “iLAP start” generated in your system menus. If you want to do it automatically at system startup you have to integrate the following scripts in the boot configuration.

```
Linux/Unix:
    <iLAP Installation Path>/AAS/Genome-AAS/bin/run-usermanagement.sh
    <iLAP Installation Path>/iLAPCore/bin/catalina.sh start
```

If all applications are successfully started the web applications for Genome Usermanagement and iLAP should be accessable under specified ports at localhost. For the default settings the URLs are:

```
http://localhost:8080/iLAP/
http://localhost:9080/UsermanagementWeb/
```

Chapter 2

Usermanagement administration

2.1 Genome Usermanagement

The Genome Usermanagement is a stand-alone web application and uses it's own features for it's administration. The web-application can be accessed under:

`http://localhost:9080/UsermanagementWeb/`

2.1.1 Login into the usermanagement

In order to administer the users of iLAP login as administrator with:

```
Username: admin
Password: can be specified during the wizard installation
```

Attention: As soon as you are logged in please change the default password of the administrator so that no unauthorized persons can administer your accounts!
In order to allow your users to login into iLAP you have to perform 4 steps:

1. Create once the organizational unit namely "institute" to which your users are belonging to. Most of the time it is the name of your own organization but if you want to subdivide your users in several working groups create them separately.
2. Create the users who should be registered in the usermanagement and assign them to the previously generated institutes.
3. Add the users to the iLAP application
4. Add the users to the group which is allowed to login in iLAP

2.1.2 Creation of an institute within the Genome Usermanagement

An institute can be simply created by clicking first on the institutes menu where create (marked with 1) appears as sub-menu. There you have to fill in the necessary attributes (marked with 2) of your institute and than simply submit the form. After this action in the user creation masks the newly created institute can be selected.

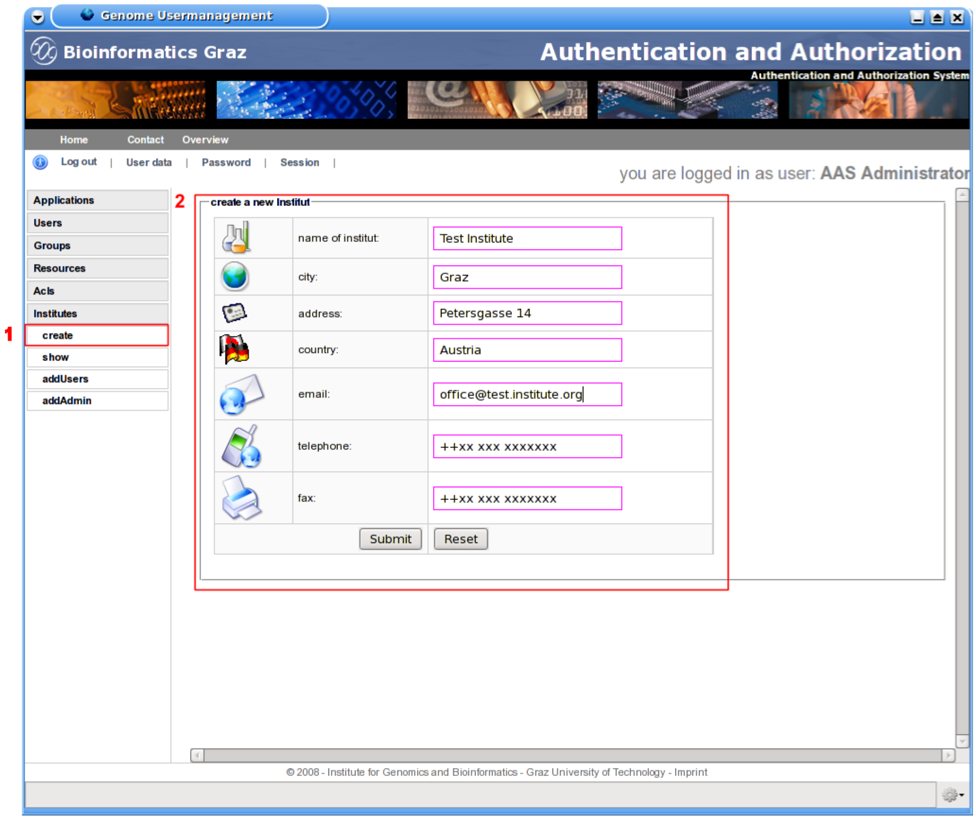

2.1.3 Creation of a user account for iLAP within the Genome Usermanagement

The user can be created on the left-hand menu under “Users” and sub-menu “Create” (marked with 1) where you have to fill in the personal data (marked with 2) as well as select the institute the user is belonging to. This influences also the sharing functionality of projects on user and institutes level. The creation is executed by pressing “Create”. In this way the user is known to the usermanagement and can login there for changing the password etc.

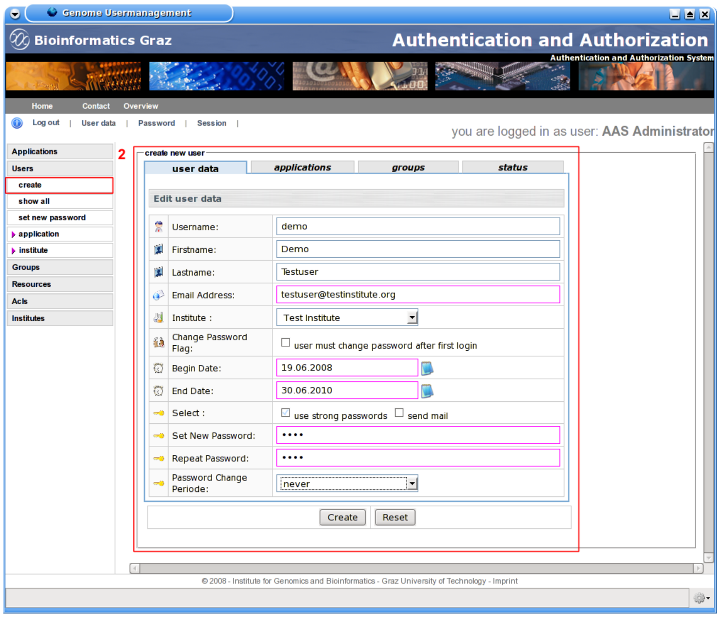

Because with the Genome Usermanagement multiple web applications but also Linux/Windows system authentication and authorization can be managed it is necessary to add the new user to the iLAP application so that the user is also known to iLAP.

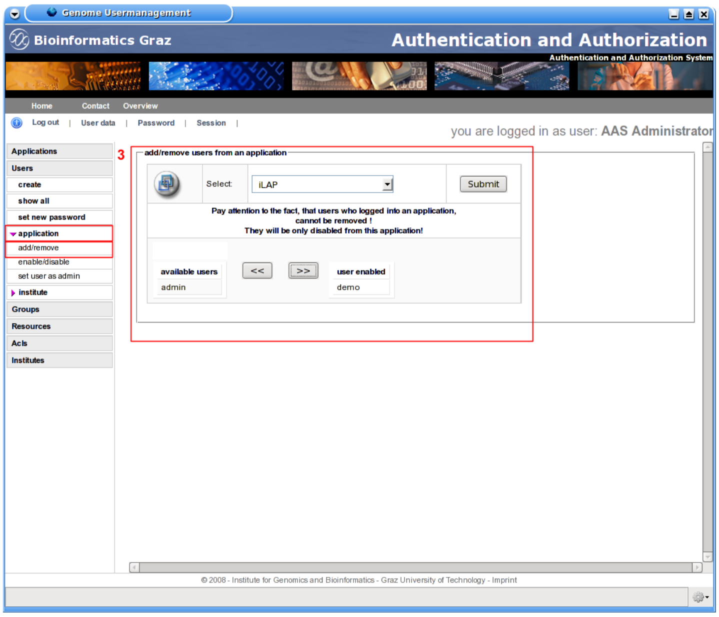

This is depicted in the screen shot above where the user is assigned using the left-hand-menu “Users”, sub-menu “Applications” subsub-menu “add/remove”. With the there appearing selec-

tion list iLAP can be selected and the users can be assigned to the application. Last but not least the user must get the authorizations within iLAP which is assigned by adding the user to the appropriate ROLE group. The basic role a iLAP user must posses is the ILAP_ROLE_USER.

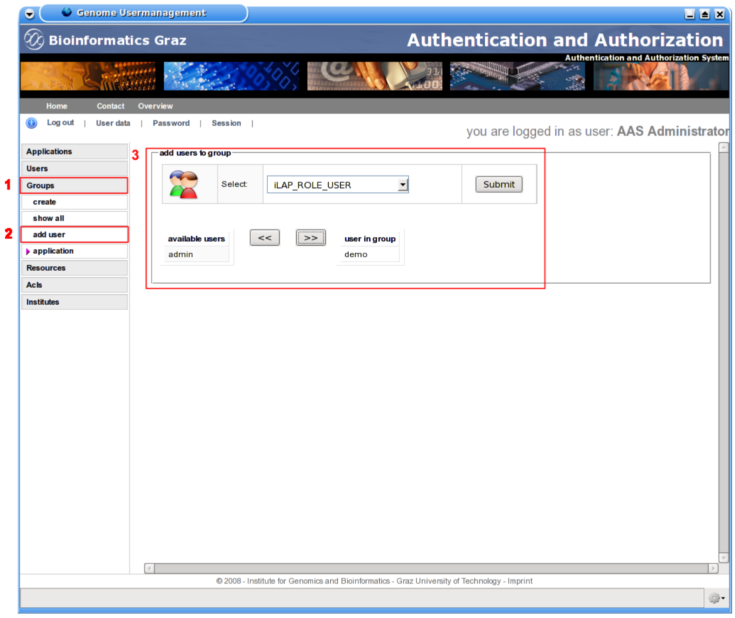

After this 4 steps your users should be able to login and work within iLAP.

2.2 Simple Usermanagement

The Simple Usermanagement is using a simple xml file which can be manipulated either with an interactive command line tool started with:

```
Linux/Unix:
      <iLAP Installation Path>/AAS/Simple/bin/sum_manager.sh
```

or by manually editing the xml file:

```
      <iLAP Installation Path>/AAS/Simple-AAS/etc/users.xml
```

Chapter 3

Testing iLAP without local installation

3.1 iLAP demo site

If you simply want to get a basic impression about the capabilities of iLAP please visit our demo site under:

`https://ilapdemo.genome.tugraz.at`

It will be frequently updated with the latest development version so that you can get an impression in which direction development is going.

3.2 Virtual image for quick iLAP testing

If you want to do some quick test of iLAP in your local environment or if you have a powerful Virtual Server environment than you can simply download the iLAP OVF-image from:

`http://genome.tugraz.at/iLAP/iLAPdownload.shtml`

It contains a CentOS 5.3 Linux installation with the complete installation of iLAP including the Genome Usermanagement and it's integration into the Apache mod_proxy front end environment. You simply can connect to the remote ip address using

`http://your.dynamic.vmwareip.address/iLAP/`

`http://your.dynamic.vmwareip.address/UsermanagementWeb/`

The login data for the image are:

```
System Users:
   root     igb-vmware5
Genome Usermanagement:
   admin    igb-vmware5
   demo     demo
```

Please be patient during boot time because it takes some time to start the web applications.

Chapter 4

FAQs

4.1 How to change the application data path of iLAP manually?

Use the editor of you choice and edit the property data_root_path in the properties file ilap-configuration.properties under the directory:

```
<iLAP Installation Path>/iLAPCore/webapps/iLAP/WEB-INF/classes
```

Attention: Under Windows operating systems the file might look strange. This is because your editor does not support Unix-documents. There the line break characters of the document are different than under windows. Make sure that your editor supports Unix document file format e.g. wordpad.

4.2 How to change the layout of iLAP manually?

Use the editor of you choice and edit the properties file iLAPWebTier.properties under:

```
<iLAP Installation Path>/iLAPCore/webapps/iLAP/WEB-INF
```

Attention: Under Windows operating systems the file might look strange. This is because your editor does not support Unix-documents. There the line break characters of the document are different than under Windows. Make sure that your editor supports Unix document file format e.g. wordpad.

4.3 How to uninstall iLAP?

If you really want to uninstall iLAP you can do this by simply executing the uninstaller.jar file under the <Path to the iLAP installation>/Uninstaller directory.

```
Linux/Unix  : java -jar Uninstaller/uninstaller.jar
Windows     : double click on the uninstaller.jar
```

This removes all files generated during the installations.
Attention: If you force the removal of the base installation directory also all the data files are gone.

4.4 Under which license is iLAP available?

GNU AFFERO GENERAL PUBLIC LICENSE
Version 3, 19 November 2007

Copyright (C) 2007 Free Software Foundation, Inc. <http://fsf.org/>
Everyone is permitted to copy and distribute verbatim copies
of this license document, but changing it is not allowed.

Preamble

The GNU Affero General Public License is a free, copyleft license for software and other kinds of works, specifically designed to ensure cooperation with the community in the case of network server software.

The licenses for most software and other practical works are designed to take away your freedom to share and change the works. By contrast, our General Public Licenses are intended to guarantee your freedom to share and change all versions of a program--to make sure it remains free software for all its users.

When we speak of free software, we are referring to freedom, not price. Our General Public Licenses are designed to make sure that you have the freedom to distribute copies of free software (and charge for them if you wish), that you receive source code or can get it if you want it, that you can change the software or use pieces of it in new free programs, and that you know you can do these things.

Developers that use our General Public Licenses protect your rights with two steps: (1) assert copyright on the software, and (2) offer you this License which gives you legal permission to copy, distribute and/or modify the software.

A secondary benefit of defending all users' freedom is that improvements made in alternate versions of the program, if they receive widespread use, become available for other developers to incorporate. Many developers of free software are heartened and encouraged by the resulting cooperation. However, in the case of software used on network servers, this result may fail to come about. The GNU General Public License permits making a modified version and letting the public access it on a server without ever releasing its source code to the public.

The GNU Affero General Public License is designed specifically to ensure that, in such cases, the modified source code becomes available to the community. It requires the operator of a network server to provide the source code of the modified version running there to the users of that server. Therefore, public use of a modified version, on a publicly accessible server, gives the public access to the source code of the modified version.

An older license, called the Affero General Public License and published by Affero, was designed to accomplish similar goals. This is a different license, not a version of the Affero GPL, but Affero has released a new version of the Affero GPL which permits relicensing under this license.

The precise terms and conditions for copying, distribution and modification follow.

TERMS AND CONDITIONS

0. Definitions.

"This License" refers to version 3 of the GNU Affero General Public License.

"Copyright" also means copyright-like laws that apply to other kinds of works, such as semiconductor masks.

"The Program" refers to any copyrightable work licensed under this License. Each licensee is addressed as "you". "Licensees" and "recipients" may be individuals or organizations.

To "modify" a work means to copy from or adapt all or part of the work in a fashion requiring copyright permission, other than the making of an exact copy. The resulting work is called a "modified version" of the earlier work or a work "based on" the earlier work.

A "covered work" means either the unmodified Program or a work based on the Program.

To "propagate" a work means to do anything with it that, without permission, would make you directly or secondarily liable for infringement under applicable copyright law, except executing it on a computer or modifying a private copy. Propagation includes copying, distribution (with or without modification), making available to the public, and in some countries other activities as well.

To "convey" a work means any kind of propagation that enables other parties to make or receive copies. Mere interaction with a user through a computer network, with no transfer of a copy, is not conveying.

An interactive user interface displays "Appropriate Legal Notices" to the extent that it includes a convenient and prominently visible feature that (1) displays an appropriate copyright notice, and (2) tells the user that there is no warranty for the work (except to the extent that warranties are provided), that licensees may convey the work under this License, and how to view a copy of this License. If the interface presents a list of user commands or options, such as a menu, a prominent item in the list meets this criterion.

1. Source Code.

The "source code" for a work means the preferred form of the work for making modifications to it. "Object code" means any non-source

form of a work.

A "Standard Interface" means an interface that either is an official standard defined by a recognized standards body, or, in the case of interfaces specified for a particular programming language, one that is widely used among developers working in that language.

The "System Libraries" of an executable work include anything, other than the work as a whole, that (a) is included in the normal form of packaging a Major Component, but which is not part of that Major Component, and (b) serves only to enable use of the work with that Major Component, or to implement a Standard Interface for which an implementation is available to the public in source code form. A "Major Component", in this context, means a major essential component (kernel, window system, and so on) of the specific operating system (if any) on which the executable work runs, or a compiler used to produce the work, or an object code interpreter used to run it.

The "Corresponding Source" for a work in object code form means all the source code needed to generate, install, and (for an executable work) run the object code and to modify the work, including scripts to control those activities. However, it does not include the work's System Libraries, or general-purpose tools or generally available free programs which are used unmodified in performing those activities but which are not part of the work. For example, Corresponding Source includes interface definition files associated with source files for the work, and the source code for shared libraries and dynamically linked subprograms that the work is specifically designed to require, such as by intimate data communication or control flow between those subprograms and other parts of the work.

The Corresponding Source need not include anything that users can regenerate automatically from other parts of the Corresponding Source.

The Corresponding Source for a work in source code form is that

same work.

2. Basic Permissions.

All rights granted under this License are granted for the term of copyright on the Program, and are irrevocable provided the stated conditions are met. This License explicitly affirms your unlimited permission to run the unmodified Program. The output from running a covered work is covered by this License only if the output, given its content, constitutes a covered work. This License acknowledges your rights of fair use or other equivalent, as provided by copyright law.

You may make, run and propagate covered works that you do not convey, without conditions so long as your license otherwise remains in force. You may convey covered works to others for the sole purpose of having them make modifications exclusively for you, or provide you with facilities for running those works, provided that you comply with the terms of this License in conveying all material for which you do not control copyright. Those thus making or running the covered works for you must do so exclusively on your behalf, under your direction and control, on terms that prohibit them from making any copies of your copyrighted material outside their relationship with you.

Conveying under any other circumstances is permitted solely under the conditions stated below. Sublicensing is not allowed; section 10 makes it unnecessary.

3. Protecting Users' Legal Rights From Anti-Circumvention Law.

No covered work shall be deemed part of an effective technological measure under any applicable law fulfilling obligations under article 11 of the WIPO copyright treaty adopted on 20 December 1996, or similar laws prohibiting or restricting circumvention of such measures.

When you convey a covered work, you waive any legal power to forbid

```
circumvention of technological measures to the extent such circumvention
is effected by exercising rights under this License with respect to
the covered work, and you disclaim any intention to limit operation or
modification of the work as a means of enforcing, against the work's
users, your or third parties' legal rights to forbid circumvention of
technological measures.

  4. Conveying Verbatim Copies.

  You may convey verbatim copies of the Program's source code as you
receive it, in any medium, provided that you conspicuously and
appropriately publish on each copy an appropriate copyright notice;
keep intact all notices stating that this License and any
non-permissive terms added in accord with section 7 apply to the code;
keep intact all notices of the absence of any warranty; and give all
recipients a copy of this License along with the Program.

  You may charge any price or no price for each copy that you convey,
and you may offer support or warranty protection for a fee.

  5. Conveying Modified Source Versions.

  You may convey a work based on the Program, or the modifications to
produce it from the Program, in the form of source code under the
terms of section 4, provided that you also meet all of these conditions:

    a) The work must carry prominent notices stating that you modified
    it, and giving a relevant date.

    b) The work must carry prominent notices stating that it is
    released under this License and any conditions added under section
    7.  This requirement modifies the requirement in section 4 to
    "keep intact all notices".

    c) You must license the entire work, as a whole, under this
    License to anyone who comes into possession of a copy.  This
```

```
    License will therefore apply, along with any applicable section 7
    additional terms, to the whole of the work, and all its parts,
    regardless of how they are packaged.  This License gives no
    permission to license the work in any other way, but it does not
    invalidate such permission if you have separately received it.

    d) If the work has interactive user interfaces, each must display
    Appropriate Legal Notices; however, if the Program has interactive
    interfaces that do not display Appropriate Legal Notices, your
    work need not make them do so.

  A compilation of a covered work with other separate and independent
works, which are not by their nature extensions of the covered work,
and which are not combined with it such as to form a larger program,
in or on a volume of a storage or distribution medium, is called an
"aggregate" if the compilation and its resulting copyright are not
used to limit the access or legal rights of the compilation's users
beyond what the individual works permit.  Inclusion of a covered work
in an aggregate does not cause this License to apply to the other
parts of the aggregate.

  6. Conveying Non-Source Forms.

  You may convey a covered work in object code form under the terms
of sections 4 and 5, provided that you also convey the
machine-readable Corresponding Source under the terms of this License,
in one of these ways:

    a) Convey the object code in, or embodied in, a physical product
    (including a physical distribution medium), accompanied by the
    Corresponding Source fixed on a durable physical medium
    customarily used for software interchange.

    b) Convey the object code in, or embodied in, a physical product
    (including a physical distribution medium), accompanied by a
    written offer, valid for at least three years and valid for as
```

long as you offer spare parts or customer support for that product model, to give anyone who possesses the object code either (1) a copy of the Corresponding Source for all the software in the product that is covered by this License, on a durable physical medium customarily used for software interchange, for a price no more than your reasonable cost of physically performing this conveying of source, or (2) access to copy the Corresponding Source from a network server at no charge.

c) Convey individual copies of the object code with a copy of the written offer to provide the Corresponding Source. This alternative is allowed only occasionally and noncommercially, and only if you received the object code with such an offer, in accord with subsection 6b.

d) Convey the object code by offering access from a designated place (gratis or for a charge), and offer equivalent access to the Corresponding Source in the same way through the same place at no further charge. You need not require recipients to copy the Corresponding Source along with the object code. If the place to copy the object code is a network server, the Corresponding Source may be on a different server (operated by you or a third party) that supports equivalent copying facilities, provided you maintain clear directions next to the object code saying where to find the Corresponding Source. Regardless of what server hosts the Corresponding Source, you remain obligated to ensure that it is available for as long as needed to satisfy these requirements.

e) Convey the object code using peer-to-peer transmission, provided you inform other peers where the object code and Corresponding Source of the work are being offered to the general public at no charge under subsection 6d.

A separable portion of the object code, whose source code is excluded from the Corresponding Source as a System Library, need not be included in conveying the object code work.

A "User Product" is either (1) a "consumer product", which means any tangible personal property which is normally used for personal, family, or household purposes, or (2) anything designed or sold for incorporation into a dwelling. In determining whether a product is a consumer product, doubtful cases shall be resolved in favor of coverage. For a particular product received by a particular user, "normally used" refers to a typical or common use of that class of product, regardless of the status of the particular user or of the way in which the particular user actually uses, or expects or is expected to use, the product. A product is a consumer product regardless of whether the product has substantial commercial, industrial or non-consumer uses, unless such uses represent the only significant mode of use of the product.

"Installation Information" for a User Product means any methods, procedures, authorization keys, or other information required to install and execute modified versions of a covered work in that User Product from a modified version of its Corresponding Source. The information must suffice to ensure that the continued functioning of the modified object code is in no case prevented or interfered with solely because modification has been made.

If you convey an object code work under this section in, or with, or specifically for use in, a User Product, and the conveying occurs as part of a transaction in which the right of possession and use of the User Product is transferred to the recipient in perpetuity or for a fixed term (regardless of how the transaction is characterized), the Corresponding Source conveyed under this section must be accompanied by the Installation Information. But this requirement does not apply if neither you nor any third party retains the ability to install modified object code on the User Product (for example, the work has been installed in ROM).

The requirement to provide Installation Information does not include a requirement to continue to provide support service, warranty, or updates for a work that has been modified or installed by the recipient, or for

the User Product in which it has been modified or installed. Access to a network may be denied when the modification itself materially and adversely affects the operation of the network or violates the rules and protocols for communication across the network.

Corresponding Source conveyed, and Installation Information provided, in accord with this section must be in a format that is publicly documented (and with an implementation available to the public in source code form), and must require no special password or key for unpacking, reading or copying.

7. Additional Terms.

"Additional permissions" are terms that supplement the terms of this License by making exceptions from one or more of its conditions. Additional permissions that are applicable to the entire Program shall be treated as though they were included in this License, to the extent that they are valid under applicable law. If additional permissions apply only to part of the Program, that part may be used separately under those permissions, but the entire Program remains governed by this License without regard to the additional permissions.

When you convey a copy of a covered work, you may at your option remove any additional permissions from that copy, or from any part of it. (Additional permissions may be written to require their own removal in certain cases when you modify the work.) You may place additional permissions on material, added by you to a covered work, for which you have or can give appropriate copyright permission.

Notwithstanding any other provision of this License, for material you add to a covered work, you may (if authorized by the copyright holders of that material) supplement the terms of this License with terms:

a) Disclaiming warranty or limiting liability differently from the terms of sections 15 and 16 of this License; or

b) Requiring preservation of specified reasonable legal notices or author attributions in that material or in the Appropriate Legal Notices displayed by works containing it; or

c) Prohibiting misrepresentation of the origin of that material, or requiring that modified versions of such material be marked in reasonable ways as different from the original version; or

d) Limiting the use for publicity purposes of names of licensors or authors of the material; or

e) Declining to grant rights under trademark law for use of some trade names, trademarks, or service marks; or

f) Requiring indemnification of licensors and authors of that material by anyone who conveys the material (or modified versions of it) with contractual assumptions of liability to the recipient, for any liability that these contractual assumptions directly impose on those licensors and authors.

All other non-permissive additional terms are considered "further restrictions" within the meaning of section 10. If the Program as you received it, or any part of it, contains a notice stating that it is governed by this License along with a term that is a further restriction, you may remove that term. If a license document contains a further restriction but permits relicensing or conveying under this License, you may add to a covered work material governed by the terms of that license document, provided that the further restriction does not survive such relicensing or conveying.

If you add terms to a covered work in accord with this section, you must place, in the relevant source files, a statement of the additional terms that apply to those files, or a notice indicating where to find the applicable terms.

Additional terms, permissive or non-permissive, may be stated in the

form of a separately written license, or stated as exceptions;
the above requirements apply either way.

8. Termination.

You may not propagate or modify a covered work except as expressly provided under this License. Any attempt otherwise to propagate or modify it is void, and will automatically terminate your rights under this License (including any patent licenses granted under the third paragraph of section 11).

However, if you cease all violation of this License, then your license from a particular copyright holder is reinstated (a) provisionally, unless and until the copyright holder explicitly and finally terminates your license, and (b) permanently, if the copyright holder fails to notify you of the violation by some reasonable means prior to 60 days after the cessation.

Moreover, your license from a particular copyright holder is reinstated permanently if the copyright holder notifies you of the violation by some reasonable means, this is the first time you have received notice of violation of this License (for any work) from that copyright holder, and you cure the violation prior to 30 days after your receipt of the notice.

Termination of your rights under this section does not terminate the licenses of parties who have received copies or rights from you under this License. If your rights have been terminated and not permanently reinstated, you do not qualify to receive new licenses for the same material under section 10.

9. Acceptance Not Required for Having Copies.

You are not required to accept this License in order to receive or run a copy of the Program. Ancillary propagation of a covered work occurring solely as a consequence of using peer-to-peer transmission

to receive a copy likewise does not require acceptance. However, nothing other than this License grants you permission to propagate or modify any covered work. These actions infringe copyright if you do not accept this License. Therefore, by modifying or propagating a covered work, you indicate your acceptance of this License to do so.

10. Automatic Licensing of Downstream Recipients.

Each time you convey a covered work, the recipient automatically receives a license from the original licensors, to run, modify and propagate that work, subject to this License. You are not responsible for enforcing compliance by third parties with this License.

An "entity transaction" is a transaction transferring control of an organization, or substantially all assets of one, or subdividing an organization, or merging organizations. If propagation of a covered work results from an entity transaction, each party to that transaction who receives a copy of the work also receives whatever licenses to the work the party's predecessor in interest had or could give under the previous paragraph, plus a right to possession of the Corresponding Source of the work from the predecessor in interest, if the predecessor has it or can get it with reasonable efforts.

You may not impose any further restrictions on the exercise of the rights granted or affirmed under this License. For example, you may not impose a license fee, royalty, or other charge for exercise of rights granted under this License, and you may not initiate litigation (including a cross-claim or counterclaim in a lawsuit) alleging that any patent claim is infringed by making, using, selling, offering for sale, or importing the Program or any portion of it.

11. Patents.

A "contributor" is a copyright holder who authorizes use under this License of the Program or a work on which the Program is based. The work thus licensed is called the contributor's "contributor version".

A contributor's "essential patent claims" are all patent claims owned or controlled by the contributor, whether already acquired or hereafter acquired, that would be infringed by some manner, permitted by this License, of making, using, or selling its contributor version, but do not include claims that would be infringed only as a consequence of further modification of the contributor version. For purposes of this definition, "control" includes the right to grant patent sublicenses in a manner consistent with the requirements of this License.

Each contributor grants you a non-exclusive, worldwide, royalty-free patent license under the contributor's essential patent claims, to make, use, sell, offer for sale, import and otherwise run, modify and propagate the contents of its contributor version.

In the following three paragraphs, a "patent license" is any express agreement or commitment, however denominated, not to enforce a patent (such as an express permission to practice a patent or covenant not to sue for patent infringement). To "grant" such a patent license to a party means to make such an agreement or commitment not to enforce a patent against the party.

If you convey a covered work, knowingly relying on a patent license, and the Corresponding Source of the work is not available for anyone to copy, free of charge and under the terms of this License, through a publicly available network server or other readily accessible means, then you must either (1) cause the Corresponding Source to be so available, or (2) arrange to deprive yourself of the benefit of the patent license for this particular work, or (3) arrange, in a manner consistent with the requirements of this License, to extend the patent license to downstream recipients. "Knowingly relying" means you have actual knowledge that, but for the patent license, your conveying the covered work in a country, or your recipient's use of the covered work in a country, would infringe one or more identifiable patents in that country that you have reason to believe are valid.

If, pursuant to or in connection with a single transaction or arrangement, you convey, or propagate by procuring conveyance of, a covered work, and grant a patent license to some of the parties receiving the covered work authorizing them to use, propagate, modify or convey a specific copy of the covered work, then the patent license you grant is automatically extended to all recipients of the covered work and works based on it.

A patent license is "discriminatory" if it does not include within the scope of its coverage, prohibits the exercise of, or is conditioned on the non-exercise of one or more of the rights that are specifically granted under this License. You may not convey a covered work if you are a party to an arrangement with a third party that is in the business of distributing software, under which you make payment to the third party based on the extent of your activity of conveying the work, and under which the third party grants, to any of the parties who would receive the covered work from you, a discriminatory patent license (a) in connection with copies of the covered work conveyed by you (or copies made from those copies), or (b) primarily for and in connection with specific products or compilations that contain the covered work, unless you entered into that arrangement, or that patent license was granted, prior to 28 March 2007.

Nothing in this License shall be construed as excluding or limiting any implied license or other defenses to infringement that may otherwise be available to you under applicable patent law.

12. No Surrender of Others' Freedom.

If conditions are imposed on you (whether by court order, agreement or otherwise) that contradict the conditions of this License, they do not excuse you from the conditions of this License. If you cannot convey a covered work so as to satisfy simultaneously your obligations under this License and any other pertinent obligations, then as a consequence you may not convey it at all. For example, if you agree to terms that obligate you

to collect a royalty for further conveying from those to whom you convey the Program, the only way you could satisfy both those terms and this License would be to refrain entirely from conveying the Program.

13. Remote Network Interaction; Use with the GNU General Public License.

Notwithstanding any other provision of this License, if you modify the Program, your modified version must prominently offer all users interacting with it remotely through a computer network (if your version supports such interaction) an opportunity to receive the Corresponding Source of your version by providing access to the Corresponding Source from a network server at no charge, through some standard or customary means of facilitating copying of software. This Corresponding Source shall include the Corresponding Source for any work covered by version 3 of the GNU General Public License that is incorporated pursuant to the following paragraph.

Notwithstanding any other provision of this License, you have permission to link or combine any covered work with a work licensed under version 3 of the GNU General Public License into a single combined work, and to convey the resulting work. The terms of this License will continue to apply to the part which is the covered work, but the work with which it is combined will remain governed by version 3 of the GNU General Public License.

14. Revised Versions of this License.

The Free Software Foundation may publish revised and/or new versions of the GNU Affero General Public License from time to time. Such new versions will be similar in spirit to the present version, but may differ in detail to address new problems or concerns.

Each version is given a distinguishing version number. If the Program specifies that a certain numbered version of the GNU Affero General Public License "or any later version" applies to it, you have the option of following the terms and conditions either of that numbered

```
version or of any later version published by the Free Software
Foundation.  If the Program does not specify a version number of the
GNU Affero General Public License, you may choose any version ever published
by the Free Software Foundation.

  If the Program specifies that a proxy can decide which future
versions of the GNU Affero General Public License can be used, that proxy's
public statement of acceptance of a version permanently authorizes you
to choose that version for the Program.

  Later license versions may give you additional or different
permissions.  However, no additional obligations are imposed on any
author or copyright holder as a result of your choosing to follow a
later version.

  15. Disclaimer of Warranty.

  THERE IS NO WARRANTY FOR THE PROGRAM, TO THE EXTENT PERMITTED BY
APPLICABLE LAW.  EXCEPT WHEN OTHERWISE STATED IN WRITING THE COPYRIGHT
HOLDERS AND/OR OTHER PARTIES PROVIDE THE PROGRAM "AS IS" WITHOUT WARRANTY
OF ANY KIND, EITHER EXPRESSED OR IMPLIED, INCLUDING, BUT NOT LIMITED TO,
THE IMPLIED WARRANTIES OF MERCHANTABILITY AND FITNESS FOR A PARTICULAR
PURPOSE.  THE ENTIRE RISK AS TO THE QUALITY AND PERFORMANCE OF THE PROGRAM
IS WITH YOU.  SHOULD THE PROGRAM PROVE DEFECTIVE, YOU ASSUME THE COST OF
ALL NECESSARY SERVICING, REPAIR OR CORRECTION.

  16. Limitation of Liability.

  IN NO EVENT UNLESS REQUIRED BY APPLICABLE LAW OR AGREED TO IN WRITING
WILL ANY COPYRIGHT HOLDER, OR ANY OTHER PARTY WHO MODIFIES AND/OR CONVEYS
THE PROGRAM AS PERMITTED ABOVE, BE LIABLE TO YOU FOR DAMAGES, INCLUDING ANY
GENERAL, SPECIAL, INCIDENTAL OR CONSEQUENTIAL DAMAGES ARISING OUT OF THE
USE OR INABILITY TO USE THE PROGRAM (INCLUDING BUT NOT LIMITED TO LOSS OF
DATA OR DATA BEING RENDERED INACCURATE OR LOSSES SUSTAINED BY YOU OR THIRD
PARTIES OR A FAILURE OF THE PROGRAM TO OPERATE WITH ANY OTHER PROGRAMS),
EVEN IF SUCH HOLDER OR OTHER PARTY HAS BEEN ADVISED OF THE POSSIBILITY OF
```

```
SUCH DAMAGES.

  17. Interpretation of Sections 15 and 16.

  If the disclaimer of warranty and limitation of liability provided
above cannot be given local legal effect according to their terms,
reviewing courts shall apply local law that most closely approximates
an absolute waiver of all civil liability in connection with the
Program, unless a warranty or assumption of liability accompanies a
copy of the Program in return for a fee.

                     END OF TERMS AND CONDITIONS

            How to Apply These Terms to Your New Programs

  If you develop a new program, and you want it to be of the greatest
possible use to the public, the best way to achieve this is to make it
free software which everyone can redistribute and change under these terms.

  To do so, attach the following notices to the program.  It is safest
to attach them to the start of each source file to most effectively
state the exclusion of warranty; and each file should have at least
the "copyright" line and a pointer to where the full notice is found.

    <one line to give the program's name and a brief idea of what it does.>
    Copyright (C) <year>  <name of author>

    This program is free software: you can redistribute it and/or modify
    it under the terms of the GNU Affero General Public License as published by
    the Free Software Foundation, either version 3 of the License, or
    (at your option) any later version.

    This program is distributed in the hope that it will be useful,
    but WITHOUT ANY WARRANTY; without even the implied warranty of
    MERCHANTABILITY or FITNESS FOR A PARTICULAR PURPOSE.  See the
    GNU Affero General Public License for more details.
```

You should have received a copy of the GNU Affero General Public License along with this program. If not, see <http://www.gnu.org/licenses/>.

Also add information on how to contact you by electronic and paper mail.

If your software can interact with users remotely through a computer network, you should also make sure that it provides a way for users to get its source. For example, if your program is a web application, its interface could display a "Source" link that leads users to an archive of the code. There are many ways you could offer source, and different solutions will be better for different programs; see section 13 for the specific requirements.

You should also get your employer (if you work as a programmer) or school, if any, to sign a "copyright disclaimer" for the program, if necessary. For more information on this, and how to apply and follow the GNU AGPL, see <http://www.gnu.org/licenses/>.
