# Supplementary material for: iLAP: a workflow-driven software for experimental protocol development, data acquisition and analysis
Source: BMC Bioinformatics. 2009 Nov 26;10:390. doi: 10.1186/1471-2105-10-390 (PMC2789074; doi:10.1186/1471-2105-10-390)
Supplement: Additional file 3 — m-FISH protocol in PDF format. This file contains the combinatorial multiple fluorescence in situ hybridization (m-FISH) protocol in PDF format used in the case study section. [file 1471-2105-10-390-S3.PDF]

Title: CWP of 3D combinatorial DNA-MFISH of BAC1-BAC7 - preconfluent CSK Fix_2008-10-24

1. Cell preparation / Probe preparation

cells and FISH probes are prepared for hybridization in parallel

1.1. DNA FISH probe preparation

FISH probe preparation: precipitate probe DNA together with human Cot1 and salmon sperm DNA and dissolve in hybridization buffer

1.1.1. prepare DNA mix for EtOH precipitation

Mix the following: - ~100 ng nick-translated BAC-probe-DNA for each target gene - 0.7 x 2/3 x n µl salmon sperm DNA 10 mg/ml (n=number of total probes used for combinatorial labeling. e.g. 7 targets n = 12) - 5 x 2/3 x n µl cot1 DNA 1 mg/ml (n=number of total probes used for combinatorial labeling. e.g. 7 targets n = 12) - 11.3 x 2/3 x n µl ddH20 (n=number of total probes used for combinatorial labeling. e.g. 7 targets n = 12) - 2 x Vol. abs. EtOH - 1/10 x Vol. 3M sodium acetate pH 5.2

Parameters:

Name	Value	Default value	Man.	Description
nick-translated DNA	100.0 ng	100 ng	-	amount of nick-translated DNA per target gene
salmon sperm DNA	5.6 µl	5.6 µl	-	amount of salmon sperm DNA 10 mg/ml: 0.7 x 2/3 x n µl n=number of total probes used for combinatorial labeling. e.g. 7 targets n = 12
human cot1 DNA	40.0 µl	40 µl	-	amount of human cot1 DNA 1 mg/ml 5 x 2/3 x n µl n=number of total probes used for combinatorial labeling. e.g. 7 targets n = 12
H2O	90.4 µl	90.4 µl	-	volume of H2O 11.3 x 2/3 x n n=number of total probes used for combinatorial labeling. e.g. 7 targets n = 12
sodium acteate	17.0 µl	17 µl	-	volume of sodium acteate 1/10 x Vol.
abs. EtOH	340.0 µl	340 µl	-	volume of abs. EtOH 2 x Vol.

1.1.2. put the DNA mixture at -70°C

put the DNA mixture for 30 min. at -70°C

Parameters:

Name	Value	Default value	Man.	Description
temperature	-70.0 °C	-70 °C	-	temperature
time	30.0 minutes	30 minutes	-	time

1.1.3. spin down in a centrifuge

spin the precipitated DNA down in a centrifuge

Parameters:

Name	Value	Default value	Man.	Description
centrifugation speed	13000.0 rpm	13000 rpm	-	centrifugation speed
centrifugation temperature	4 °C	4 °C	-	centrifugation temperature
centrifugation time	15.0 minutes	15 minutes	-	centrifugation time

1.1.4. remove supernantant

remove supernantant by pipetting carefully to not disturb the pellet.

1.1.5. rinse with 70% EtOH

rinse pellet with 70% EtOH to remove salts: - add 80 µl 70% EtOH - centrifuge for 5 minutes

1.1.6. spin down in a centrifuge

spin the precipitated DNA down in a centrifuge

Parameters:

Name	Value	Default value	Man.	Description
centrifugation speed	13000.0 rpm	13000 rpm	-	centrifugation speed
centrifugation temperature	4 °C	4 °C	-	centrifugation temperature
centrifugation time	5.0 minutes	5 minutes	-	centrifugation time

1.1.7. remove supernantant

remove supernantant by pipetting carefully to not disturb the pellet.

1.1.8. dry pellet

dry pellet at room temperature

Parameters:

Name	Value	Default value	Man.	Description
temperature	25.0 °C	25 °C	-	drying temperature
time	20.0 minutes	20 minutes	-	drying time

1.1.9. add formamide

add formamide to a final concentration of 50%

Parameters:

Name	Value	Default value	Man.	Description
volume	2.5 µl	2.5 µl	-	volume of formamide to add to the dried pellet

1.1.10. add H2O

add 1 µl H2O

Parameters:

Name	Value	Default value	Man.	Description
volume	1.0 µl	1 µl	-	volume of H20 to add

1.1.11. dissolve at 37°C

dissolve at 37°C for 20 minutes and vortex in between.

Parameters:

Name	Value	Default value	Man.	Description
temperature	37.0 °C	37 °C	-	dissolving temperature
time	20.0 minutes	20 minutes	-	dissolving time

1.1.12. add 20xSSC

add 20xSSC to a final concentration of 2xSSC

Parameters:

Name	Value	Default value	Man.	Description
volume	0.5 µl	0.5 µl	-	volume of 20xSSC to add

1.1.13. add dextrane sulfate

add 25% dextrane sulfate to a final concentration of 5% mix well before use

Parameters:

Name	Value	Default value	Man.	Description
volume	1.0 µl	1 µl	-	volume of 25% dextrane sulfate to add

1.1.14. Denaturation of the DNA probe

denaturate the hybridization mix (probe) - begin with denaturation while the cells are being treated with 2xSSC (see cell preparation)

Parameters:

Name	Value	Default value	Man.	Description
temperature	85.0 °C	85 °C	-	denaturation temperature

Parameters:

Name	Value	Default value	Man.	Description
time	10.0 minutes	10 minutes	-	denaturation time

1.1.15. prehybridize the probe

prehybridize the probe

Parameters:

Name	Value	Default value	Man.	Description
time	20.0 minutes	20 minutes	-	prehybridization time
temperature	37.0 °C	37 °C	-	prehybridization temperature

1.2. Cell preparation

cell are prepared for hybridization. - permeabilized - fixed - RNAse treated

1.2.1. cell permeabilization / extraction

permeabilize / extract cells in CSK Buffer CSK BUffer: 100 mM NaCl 300 mM Sucrose 10 mM PIPES pH 6.8 3 mM MgCl2 0.5 % Triton-X 100 0.5 % DEPC (not really needed for DNA-FISH) 2 mM VRC (Vanadyl Ribonucleoside Complexes) (not really needed for DNA-FISH)

Parameters:

Name	Value	Default value	Man.	Description
temperature	4.0 °C	4 °C	-	extraction temperature
time	1.0 minutes	1 minutes	-	extraction time

1.2.2. cell fixation

fix the cells after permeabilization / extraction in 3.5% PFA/PBS--

Parameters:

Name	Value	Default value	Man.	Description
time	25.0 minutes	25 minutes	-	fixation time
temperature	25.0 °C	25 °C	-	fixation temperature

1.2.3. wash in PBS--

wash the fixed cells in PBS-- after fixation, to remove the remaining PFA

Parameters:

Name	Value	Default value	Man.	Description
time	5.0 minutes	5 minutes	-	washing time
repeats	2.0	2	-	washing step repeats
temperature	25.0 °C	25 °C	-	washing temperature

1.2.4. store cells in 70% EtOH

cells are in 70% EtOH for future use in FISH-experiment. The cells should then be rehydrated for 10 min. in PBS--.

Parameters:

Name	Value	Default value	Man.	Description
temperature	4.0 °C	4 °C	-	storing temperature
time	2.0 days	2 days	-	time that the cells were stored in EtOH

1.2.5. rehydrate the stored cells

rehydrate the EtOH stored cells in PBS--.

Parameters:

Name	Value	Default value	Man.	Description
time	10.0 minutes	10 minutes	-	rehydration time
temperature	25.0 °C	25 °C	-	rehydration temperature

1.2.6. cut the coverslip into 1/4s

cut the coverslip into 1/4s in order to save some reagents.

1.2.7. RNAse treatment

treat the fixed cells with RNAse-A (25 µg/ml in PBS--) in order to get rid of the RNA (we want to detect DNA only)

Parameters:

Name	Value	Default value	Man.	Description
concentration	25.0 µg/ml	25 µg/ml	-	RNAse-A concentration in PBS--
time	20.0 minutes	20 minutes	-	RNAse treatment time
temperature	25.0 °C	25 °C	-	RNAse treatment temperature

1.2.8. wash in PBS--

wash the RNAse treated cells in PBS-- in order to remove the excessive RNAse

Parameters:

Name	Value	Default value	Man.	Description
time	10.0 minutes	5 minutes	-	washing time
temperature	25.0 °C	25 °C	-	washing temperature
repeats	3.0	3	-	washing step repeats

1.2.9. 2xSSC treatment

treat the cells with 2xSSC (start denaturating the probe while treating the cells with 2xSSC)

Parameters:

Name	Value	Default value	Man.	Description
time	3.0 minutes	10 minutes	-	2xSSC treatment time

1.2.10. 2xSSC / 70% Formamide treatment

treat the cells with 2xSSC / 70% Formamide. Use deionized formamide!

Parameters:

Name	Value	Default value	Man.	Description
time	3.0 minutes	3 minutes	-	2xSSC / 70% Formamide treatment time

1.2.11. heat denaturate the chromatin

heat denaturate the chromatin in order to make the DNA accessible for probe hybridization. This step is performed on a heating plate (Grant Boekel). Place a microscope slide onto the plate and apply a drop of 70% Formamide / 2xSSC (ca. 200µl) onto it. Let it preheat to the denaturation temperature and then put the coverslip (1/4) upside down (cells down) onto it.

Parameters:

Name	Value	Default value	Man.	Description
time	4.5 minutes	4.5 minutes	-	denaturation time
temperature	85.0 °C	85 °C	-	denaturation temperature

1.2.12. dehydrate the cells

dehydrate the cells in a EtOH series.

1.2.12.1. wash with 70% EtOH

wash with 70% EtOH

Parameters:

Name	Value	Default value	Man.	Description
time	5.0 minutes	5 minutes	-	washing time
temperature	4.0 °C	4 °C	-	washing temperature

1.2.12.2. wash with 90% EtOH

wash with 90% EtOH

Parameters:

Name	Value	Default value	Man.	Description
time	5.0 minutes	5 minutes	-	washing time
temperature	4.0 °C	4 °C	-	washing temperature

1.2.12.3. wash with 100% EtOH

wash with 100% EtOH

Parameters:

Name	Value	Default value	Man.	Description
time	5.0 minutes	5 minutes	-	washing time
temperature	4.0 °C	4 °C	-	washing temperature

1.2.12.4. dry the cells at RT

dry the cells at room temperature fo 5 min.

Parameters:

Name	Value	Default value	Man.	Description
time	5.0 minutes	5 minutes	-	drying time
temperature	25.0 °C	25 °C	-	drying temperature

2. Hybridization

Hybridization over night at 37°C in humidified chamber

2.1. apply the probe to the dehydrated cells

apply the probe to the dehydrated cells, by pipetting the 5 µl onto a glass slide and putting the coverslip upside down onto the probe drop. Avoid bubbles!

2.2. seal with rubber cement

seal with rubber cement

2.3. hybridize over 2 nights

hybridize over 2 nights at 37° C in a humidified chamber.

Parameters:

Name	Value	Default value	Man.	Description
time	24.0 hours	24 hours	-	hybridization time
temperature	37.0 °C	37 °C	-	hybridization temperature

3. Washes after Hybridization

Stringency washes after hybridization

3.1. 50% Formamide / 2xSSC washes

50% Formamide / 2xSSC washes preheat the 50% Formamide / 2xSSC solution to 45°C

Parameters:

Name	Value	Default value	Man.	Description
time	15.0 minutes	15 minutes	-	washing time
temperature	45.0 °C	45 °C	-	washing temperature (preheat the solution to this temp.)
repeats	2.0	2	-	washing step repeat

3.2. 0.1xSSC wash

0.1xSSC wash

Parameters:

Name	Value	Default value	Man.	Description
time	10.0 minutes	10 minutes	-	washing time
temperature	25.0 °C	25 °C	-	washing temperature

3.3. 2xSSC wash

2xSSC wash

Parameters:

Name	Value	Default value	Man.	Description
time	10.0 minutes	10 minutes	-	washing time
temperature	25.0 °C	25 °C	-	washing temperature

3.4. 4xSSC wash

4xSSC wash

Parameters:

Name	Value	Default value	Man.	Description
time	5.0 minutes	5 minutes	-	washing time
temperature	25.0 °C	25 °C	-	washing temperature

4. Probe detection

Detection of the hapten labelled probes using fluorescent antibodies.

4.1. Biotin Probe detection

detect hapten labeled probes by fluorescent antibodies

4.1.1. dilute streptavidin antibody

dilute streptavidin antibody 1:200 in 4xSSC/0.1% BSA/0.01% Tween 20

Parameters:

Name	Value	Default value	Man.	Description
dilution	1:200	1:200	-	antibody dilution
antibody	streptavidin, Alexa Fluor® 647 conjugate 2 mg/mL	streptavidin, Alexa Fluor® 647 conjugate 2 mg/mL	-	type of antibody

4.1.2. apply the antibody solution to the cells

apply the antibody solution to the cells by pipetting ~15 µl onto a glass slide and putting the coverslip upside down onto the antibody solution. Avoid air bubbles!

4.1.3. Incubate

incubate for 1 hour at 37°C in a dark humidified chamber.

Parameters:

Name	Value	Default value	Man.	Description
time	1.0 hours	1 hours	-	incubation time
temperature	37.0 °C	37 °C	-	incubation temperature

4.2. Washes after Biotin-probe detection

Washes after Biotin-probe detection

4.2.1. 4xSSC/0.1% TWEEN20 wash

4xSSC/0.1% TWEEN20 wash

Parameters:

Name	Value	Default value	Man.	Description
time	10.0 minutes	10 minutes	-	washing time
temperature	25.0 °C	25 °C	-	washing temperature

4.2.2. 4xSSC wash

4xSSC wash

Parameters:

Name	Value	Default value	Man.	Description
time	10.0 minutes	10 minutes	-	washing time
temperature	25.0 °C	25 °C	-	washing temperature
repeats	2.0	2	-	washing step repeats

4.2.3. 2xSSC wash

2xSSC wash

Parameters:

Name	Value	Default value	Man.	Description
time	10.0 minutes	10 minutes	-	washing time
temperature	25.0 °C	25 °C	-	washing temperature

4.2.4. PBS-- wash

PBS-- wash

Parameters:

Name	Value	Default value	Man.	Description
time	5.0 minutes	5 minutes	-	washing time
temperature	25.0 °C	25 °C	-	washing temperature

4.3. DNP/DIG Probe detection

detect hapten labeled probes by fluorescent antibodies

4.3.1. dilute the DNP and DIG antibodies

dilute DNP and DIG antibodies 1:200 in 4%BSA/0.1% Tween 20/PBS--

Parameters:

Name	Value	Default value	Man.	Description
dilution	1:200	1:200	-	antibody dilution
DNP antibody	anti-dinitrophenyl-KLH, rabbit IgG fraction, Alexa Fluor® 488 conjugate 2 mg/mL	anti-dinitrophenyl-KLH, rabbit IgG fraction, Alexa Fluor® 488 conjugate 2 mg/mL	-	type of antibody
DIG antibody	Anti-Digoxigenin-Rhodamine, Fab fragments (ROCHE)	Anti-Digoxigenin-Rhodamine, Fab fragments (ROCHE)	-	DIG antibody

4.3.2. apply the antibody solution to the cells

apply the antibody solution to the cells by pipetting ~15 µl onto a glass slide and putting the coverslip upside down onto the antibody solution. Avoid air bubbles!

4.3.3. Incubate

incubate for 1 hour at room temperature in a dark humidified chamber.

Parameters:

Name	Value	Default value	Man.	Description
time	1.0 hours	1 hours	-	incubation time
temperature	25.0 °C	25 °C	-	incubation temperature

4.4. Washes after DNP/DIG-probe detection

Washes after DNP/DIG-probe detection

4.4.1. PBS-- wash

PBS-- wash

Parameters:

Name	Value	Default value	Man.	Description
time	5.0 minutes	5 minutes	-	washing time
temperature	25.0 °C	25 °C	-	washing temperature
repeats	3.0	3	-	washing step repeats

5. DAPI conterstain

DAPI conterstain

Parameters:

Name	Value	Default value	Man.	Description
time	5.0 minutes	5 minutes	-	staining time
temperature	25.0 °C	25 °C	-	staining temperaure
concentration	0.05 µg/ml	0.05 µg/ml	-	DAPI concentration in PBS--

6. Mount and image

Mount with an antifade mounting solution (Slow Fade Gold, Invitrogen) and seal with nail polish. Image on Epifluorescence microscope

Notes:

upload deadline
upload deadline on May 1, 2009

Files:

pre-BAC1-7-RHO-hyb-CSK-2008-24-10-Position(1).zvi
pre-BAC1-7-RHO-hyb-CSK-2008-24-10-Position(2).zvi
